# Inference of Transposable Element Ancestry

**DOI:** 10.1371/journal.pgen.1004482

**Published:** 2014-08-14

**Authors:** Aaron C. Wacholder, Corey Cox, Thomas J. Meyer, Robert P. Ruggiero, Vijetha Vemulapalli, Annette Damert, Lucia Carbone, David D. Pollock

**Affiliations:** 1 Department of Biochemistry & Molecular Genetics, University of Colorado School of Medicine, Aurora, Colorado, United States of America; 2 Department of Behavioural Neuroscience, Oregon Health Sciences University, Portland, Oregon, United States of America; 3 Division of Neuroscience, Oregon National Primate Research Center, Beaverton, Oregon, United States of America; 4 Molecular Biology Centre, Institute for Interdisciplinary Research in Bio-Nano Sciences, Babes-Bolyai-University, Cluj-Napoca, Romania; Fred Hutchinson Cancer Research Center, United States of America

## Abstract

Most common methods for inferring transposable element (TE) evolutionary relationships are based on dividing TEs into subfamilies using shared diagnostic nucleotides. Although originally justified based on the “master gene” model of TE evolution, computational and experimental work indicates that many of the subfamilies generated by these methods contain multiple source elements. This implies that subfamily-based methods give an incomplete picture of TE relationships. Studies on selection, functional exaptation, and predictions of horizontal transfer may all be affected. Here, we develop a Bayesian method for inferring TE ancestry that gives the probability that each sequence was replicative, its frequency of replication, and the probability that each extant TE sequence came from each possible ancestral sequence. Applying our method to 986 members of the newly-discovered LAVA family of TEs, we show that there were far more source elements in the history of LAVA expansion than subfamilies identified using the CoSeg subfamily-classification program. We also identify multiple replicative elements in the *Alu*Sc subfamily in humans. Our results strongly indicate that a reassessment of subfamily structures is necessary to obtain accurate estimates of mutation processes, phylogenetic relationships and historical times of activity.

## Author Summary

The most common entities in vertebrate genomes are transposable elements (TEs), DNA sequences that have been repeatedly copied and inserted into new locations throughout the genome. Some TEs have been replicated hundreds of thousands of times, and their ecology and evolutionary history within a genome is thus critical to understanding how genome structure evolves. It was once thought that only a few “master gene” copies could replicate, while the rest were inactive (dead on arrival), but recent computational and laboratory studies have indicated that this is not the case. However, previous methods for reconstructing TE evolutionary history were not designed to solve the problem of determining the ancestral source sequence for large numbers of elements. Here, we present a new method that is. Our method surveys all likely TE ancestors and determines the probability that each modern element arose from each of its plausible ancestors. We applied our method to the gibbon-derived LAVA TE family and to the human *Alu*Sc subfamily and inferred many more source elements than indicated by previous methods. This new method will help us better understand TE evolution, including both the impact of sequence on replication and the substitution process after replication.

## Introduction

Repetitive elements may comprise two-thirds or more of most vertebrate genomes [1], and most repeat sequence is derived from transposable elements (TEs). To obtain an accurate picture of the structure and evolutionary history of vertebrate genomes, it is therefore necessary to have a good understanding of the origins and expansion histories of TEs. Early studies attempted to reconstruct the relationships among TEs by dividing extant TE sequences into subfamilies on the basis of shared high-frequency diagnostic nucleotide variants [2]–[7]. Many of these early studies, particularly in primates, were interpreted as supporting a “master gene model”, in which one or a few source elements produce large numbers of inert copy elements that are incapable of replication [8], [9]. According to this model, each subfamily represents the descendants of a particular master gene, the sequence of which is assumed to be the subfamily consensus. Later studies found evidence for multiple source elements within subfamilies [10]–[12], however, and recent empirical studies have shown that up to hundreds of elements are capable of replication when placed in a laboratory system [13]. This research suggests that subfamily classification based on diagnostic nucleotides provides only a coarse picture of what may be an intricate web of familial relationships among the TEs in the genome. However, no previously established method can accurately reconstruct relationships among thousands of TE sequences.

Our group is particularly interested in utilizing TEs to understand the genomic mutation process. In theory, TEs are extremely useful for this purpose, as mutations that accumulate after a duplication occurs should typically be almost entirely neutral, and therefore serve as an accurate reflection of the mutation process unfiltered by selection [14]. However, in the course of using TEs to investigate evolutionary processes, we discovered inconsistencies that suggested that subfamily consensus sequences produced by CoSeg, a popular program for TE subfamily classification, are not reliable for use as ancestral sequences. The main problem is that at many positions in TE alignments, far more sequences than expected differ from the subfamily consensus sequence. This leads to high apparent mutation rates at these positions if the subfamily consensus is assumed to be the ancestor of all elements in the subfamily. Instead, we inferred that many of the elements in the subfamily were produced by source elements that already differed from the subfamily consensus at one or more sites but were not identified by CoSeg. An additional limitation of CoSeg and all other current subfamily-classification methods is that they assign elements to subfamilies deterministically, without accounting for inference uncertainty. This is especially problematic for TE evolutionary studies because similarities between ancestral TEs may make it impossible to precisely determine the ancestry of any given element. These problems limit the utility of TEs for investigating evolutionary processes, and thus strongly motivate the development of a new approach.

Here, we propose a novel Bayesian Markov chain Monte Carlo (MCMC) method that predicts which sequences replicated during a TE family's evolutionary history, and reconstructs the ancestral relationships among replicating and non-replicative sequences. The method returns the posterior probability that each TE sequence was replicated from each of a set of plausible ancestral sequences, as well as the probability that each candidate ancestral sequence replicated at all. To our knowledge, the only other method specifically designed to reconstruct ancestral TE relationships that is not based on heuristic subfamily classification is that of Cordaux and colleagues [10]. These authors build a median joining network [15] of the extant elements, a maximum-parsimony based method. Although this method was an important contribution, it is deterministic, only applicable to a small number of sequences, and shares the general problems [16] of maximum-parsimony phylogenetic methods. Some authors apply phylogenetic techniques designed for inferring species relationships, such as neighbor joining methods, to reconstruct TE relationships [17], [18]. These methods implausibly assume bifurcating trees, though a single source TE may replicate itself many times.

We applied our approach to two TE families: the gibbon-specific LAVA TEs [19] and the Sc subfamily of *Alu*. The gibbon LAVAs are a novel class of element found exclusively in gibbon (Hylobatidae) species, and are composed of portions of other TEs usually found in primate genomes: L1ME5, *Alu*Sz6, and SVA_A [19]. The LAVA elements are an attractive system for understanding the evolution of TEs because their recent origin (sometime after the Gibbon divergence from other hominids 15–18 million years ago) and limited diversification [19] make the analysis of their relationships more tractable. In contrast, the *Alu*Sc family is an older inactive *Alu* subfamily (estimated to be at least 35 million years old [20]). Using our new method, we evaluated whether the likely number of replicating ancestral sequences in each TE family or subfamily differed from the number of subfamilies returned by CoSeg, whether the subfamilies previously identified are compatible with predicted ancestral relationships, and whether our method solved the problem of unrealistically high implied mutation rates at some sites. Finally, we suggest new subfamily designations in the gibbon LAVA TE family based on their probable relationships.

## Results

### Identification of CoSeg subfamilies and the problem of excess mutations

Most methods to characterize TE relationships first divide a TE family into subfamilies. Subfamily-classification methods group sequences on the basis of their nucleotide identity at “diagnostic” sites [21], [22], for example by recursively splitting subfamilies that fail a test of homogeneity [22]. By far the most popular automated subfamily classification method is CoSeg [23], a wrapper for the AluCode program [24] that is integrated with the widely-used RepeatMasker TE identification program [25]. The CoSeg/AluCode method tends to identify more subfamily structure than previous approaches, so we decided to compare results from our new program exclusively to CoSeg results. The AluCode algorithm used by CoSeg iteratively identifies sequences in a family or proposed subfamily that contains pairs of sites with nucleotide variants that co-occur more frequently than would be expected by random mutation from the subfamily consensus sequence. This pair of sites is then used to divide sequences into two new subfamilies, which may be further split by the same criteria, and so on to completion. The observation of overrepresented nucleotides at a pair of sites suggests that some sequences currently assigned to a subfamily were produced by a progenitor sequence that diverged at these sites prior to replicating. This justifies introducing a new subfamily to contain the descendants of that progenitor. After generating subfamilies, CoSeg links them using a minimum spanning tree of the subfamily consensus sequences, which is intended to represent the subfamily phylogeny.

The CoSeg algorithm was applied to 986 aligned LAVA elements (401 bp) to obtain 14 subfamilies. We noticed that some sites showed higher levels of divergence from the CoSeg-defined subfamily consensus sequences than might be expected due to mutation alone. In earlier work on human *Alu* and opossum *SINE1* TEs, we had observed similarly aberrant sites [26]. These sites suggest the existence of undiscovered replicative sequences that carry the divergent variant, so we hypothesized that CoSeg subfamily classification might be too conservative about adding new subfamilies to give a realistic picture of ancestral replicative sequence structure in *LAVA*. CoSeg implements a number of conservative measures that guide the splitting. For example, it only allows each site to be used once to split subfamilies. Additionally, split decisions are only made on the basis of a strict significance test, which means that subfamilies with high support for existing may still be rejected.

To determine the plausibility that the CoSeg subfamily consensus sequences represent all of the ancestral sequences of the TEs in the data, we developed a re-sampling test. Null expectations were obtained by resampling substitutions from the consensus sequence of each subfamily, accounting for variation in mutation rates among sites and mutation types. The substitution resampling process was replicated 1000 times to get a predicted distribution of each nucleotide at each site for each subfamily under the assumption that all differences between ancestors and descendants are due to mutation. The expected sums of deviations from these expectations were compared to the observed deviations from expectation among the real by-site nucleotide distributions in each CoSeg-inferred subfamily.

**Figure 1:**
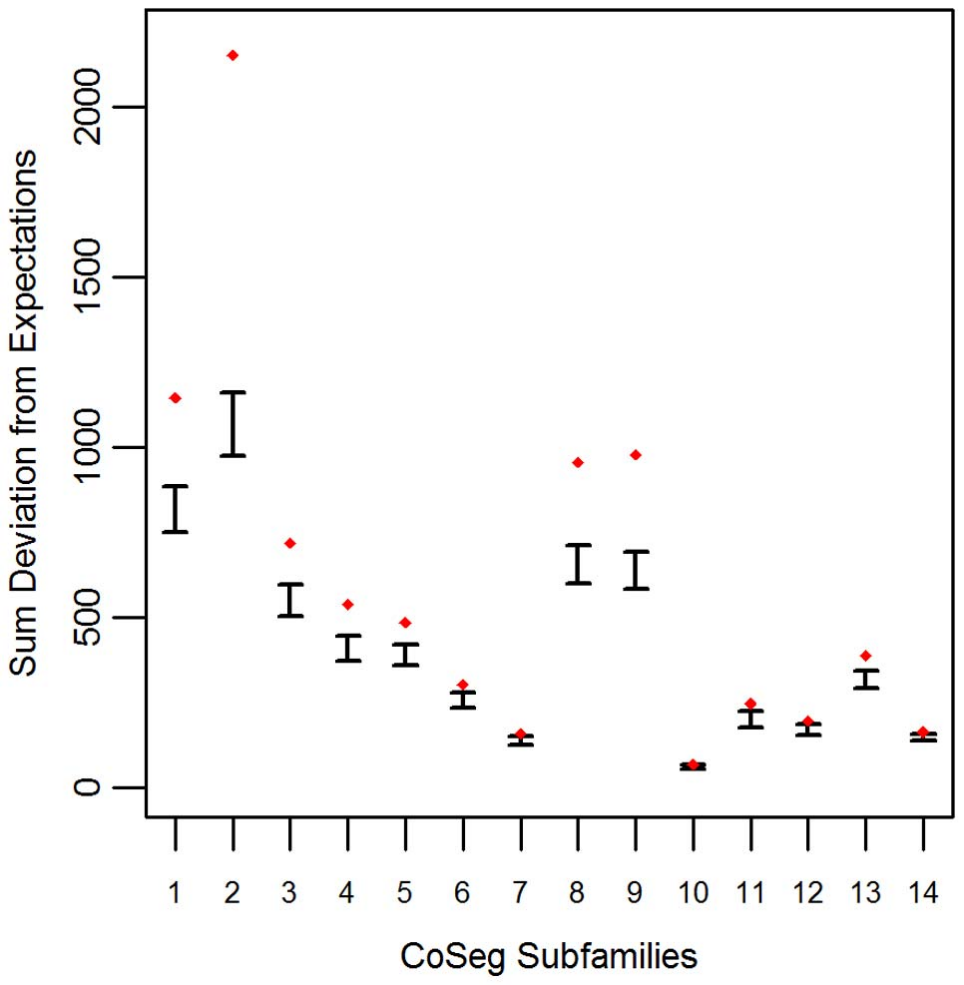
Deviation from expectation in randomly sampled CoSeg subfamilies. For each CoSeg subfamily, the 99% confidence interval is given for the deviation from expectations among 1000 substitution redraws under the hypothesis that all differences between subfamily members and the subfamily consensus are due to mutation, rather than replication. Diamonds indicate the deviation from expectation in the observed substitution data.

Applying this test to the LAVA CoSeg subfamilies, we found that, in 12 of the 14 CoSeg subfamilies, deviation from expectations exceeded the deviation among any of the 1000 resampling replicates (Figure 1). Thus, we can reject the hypothesis that the sequence data can be explained solely by substitutions from the subfamily consensuses, and infer that there are likely to be many more ancestrally-replicative sequences than identified by CoSeg.

### A Bayesian approach to TE ancestral reconstruction

To better understand the evolution of TEs, what is needed is a method that directly addresses which sequences were historically replicative and which sequences descended from each replicative element. To achieve this, we developed a novel Bayesian Markov chain Monte Carlo (MCMC) approach that jointly considers the evidence for replication of all plausible ancestral TE sequences in a family. We will refer to this method as AnTE. The first step in this method is to identify plausible discriminatory sites that separate ancestral replicative elements. We call them “discriminatory” sites to distinguish them from “diagnostic” sites that are used to deterministically classify sequences in subfamily-based methods. Discriminatory sites will tend to vary more than other sites, because replicative sequences that differ from the consensus at such sites will increase the frequency of the variant as they proliferate. Initially, the plausible discriminatory sites were identified as those sites with variant frequencies more than three standard deviations greater than the mean frequency of that variant among all sites with the same consensus base (see Methods for a full description of discriminatory site identification).

The next step of the AnTE algorithm is to construct a pool of candidate replicating ancestors; the probability that each candidate is a true ancestor can then be evaluated using the MCMC. By definition, ancestors differ from the consensus only at discriminatory sites, so only the discriminatory site sequence needs to be considered. Initially, the set of candidate ancestor discriminatory site sequences was constructed to be the set of all discriminatory site sequences observed in the sequence data. During the burn-in period of the chain, discriminatory site combinations outside the initial set of candidate ancestors were added if their inclusion improved the likelihood of the model.

The MCMC estimates posterior distributions for three sets of parameters: the relative rates of replication for each candidate (a rate of 0 indicates that the candidate is not ancestral), the times at which each candidate with non-zero replicative rate was actively replicating, and rate parameters for a nucleotide substitution rate matrix that determines the probability of transitioning between any pair of nucleotides over a time period. For any step in the MCMC procedure, the likelihood of generating the sequence data was calculated based on the inferred ancestors (i.e., sequences with non-zero replicative frequency), their replicative frequencies and times of activity at that step, and the substitution rate matrix. A prior was set on the total number of replicative sequences by giving a likelihood penalty for each candidate with non-zero replicative rate. The likelihood of each sequence observed in the data or inferred by the model was calculated based on summing the probability (see Methods, Equation 2) that it was produced by mutation from each inferred ancestral sequence, weighted by the replicative frequency of that candidate ancestor. The posterior probability distributions of the replicative frequency for each candidate sequence, whether it replicated at all, and which ancestors it was derived from, were then calculated by averaging these probabilities over all steps in the post convergence portion of the MCMC.

### Support for a large number of replicative LAVA sequences

**Figure 2:**
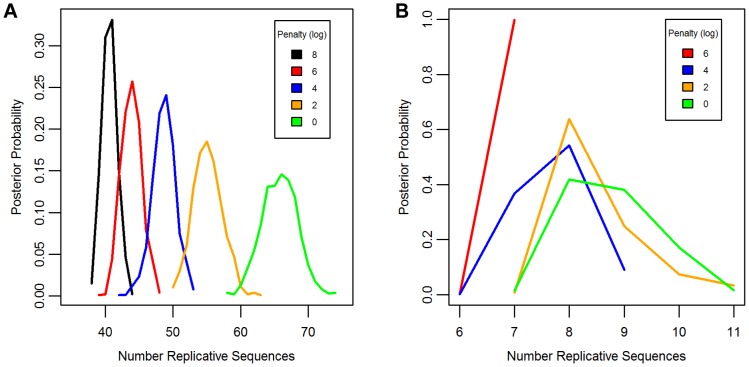
Posterior distribution of the number of replicative sequences. The Posterior distribution of the number of replicative sequences in A)LAVA and B)*Alu*Sc is given for MCMC runs with different penalties applied to each additional replicative sequence. Higher penalties indicate a prior distribution favoring fewer replicative sequences. Each distribution is an average over 10 replicates.

Separate chains were run on the LAVA and *Alu*Sc datasets for five different prior distributions of the total number of replicative sequences, set by applying a penalty on each additional ancestor inferred by the model. These penalties consisted of 0, 2, 4, 6, or 8 log points per ancestor. In LAVA, 38–43 (99% credible region) replicative sequences were inferred even under the harsh 8 log penalty, many more than the 14 subfamilies identified by the CoSeg program (Table 1 and Figure 2a). More replicative sequences were also identified for *Alu*Sc than the three subfamilies given by CoSeg, though the total number was much less than for LAVA, with 6–11 replicative sequences inferred among all priors considered (Figure 2b).

**Table 1:** Number of replicative sequences identified for different prior penalties in LAVA and *Alu*Sc.

Prior penalty (log)	Number replicative LAVA sequences (99% credible region)	Mutation-only hypothesis p-value	Number replicative *Alu*Sc Sequences (99% credible region)
0	60–72	.090	8–11
2	50–60	.064	8–11
4	44–52	<.001	7–9
6	41–47	.004	7
8	38–43	<.001	7

The same substitution resampling method applied to the CoSeg subfamilies earlier was applied to the results from each AnTE run, testing whether mutation alone can explain the differences between inferred ancestral sequences and their descendants (Table 1). Based on this analysis, we reject the mutation-only hypothesis for the LAVA runs with 8 (p<0.001), 6 (p = 0.004), or 4 (p<0.001) log penalty, inferring that these runs fail to identify some true ancestral sequences. We fail to reject the mutation-only hypothesis for the 2 log penalty run (p = .064) and the 0 log penalty run (p = .090). Thus, we select the results from the 2 log penalty chain as a conservative estimate of the number of replicative sequences in the history of LAVA, and use it in all further analyses of LAVA. Results for this chain are given in Supplementary Tables 1 and 2. The 99% credible region for the number of replicative elements in the 2 log penalty run is 50–60, suggesting 50 as a reasonable lower bound for the total number of replicative sequences. For *Alu*Sc, mutation appears to be a sufficient explanation for the differences from the inferred ancestors for all priors considered (Table 1). Since the number of sequences identified in *Alu*Sc was relatively insensitive to the prior, we present results for the same 2 log penalty as used for LAVA to facilitate comparison (Supplementary Table 3).

### A bushy network of related ancestral sequences

**Figure 3:**
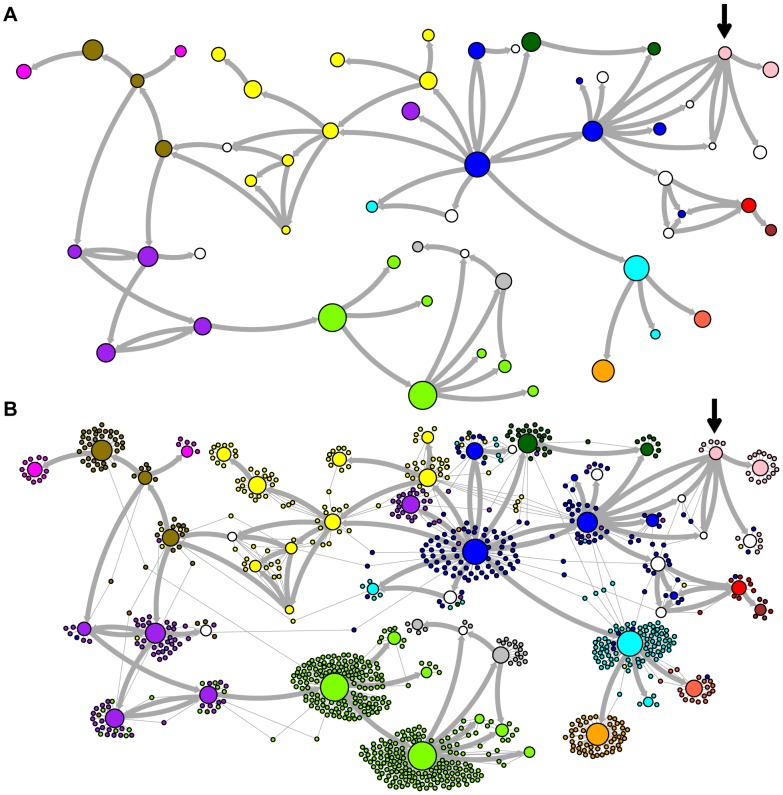
Ancestral relationships among LAVA elements. The predicted network of LAVA ancestral relationships is shown. A) All sequences that replicated with probability >30% are represented as nodes in the network. Arrows are drawn between sequences if there was at least 5% probability that an ancestral relationship existed between those sequences, with the direction of the ancestor-descendant relationships indicated by the arrows. Sequences are colored based on their CoSeg subfamily assignments (Table S2). Sequences colored white do not exist in the data, but are inferred to have existed ancestrally. B) The network in A is modified by the addition of all extant TEs in the data added to the network as nodes represented by small dots. Edges are drawn between an element and an ancestral sequence if there was at least 5% probability the element descended from the ancestral sequence. Nodes are colored based on CoSeg subfamily assignment.

**Figure 4:**
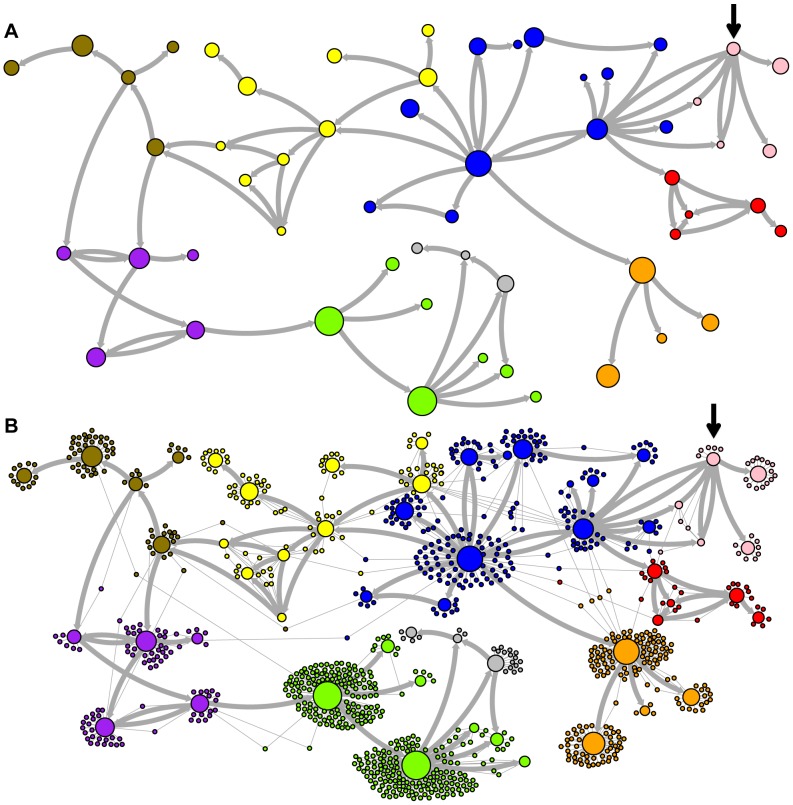
New AnTE subfamily assignments for LAVA elements. The predicted network of LAVA TE ancestral relationships is shown, as in Figure 3. A) All sequences that replicated with probability >30% are represented as nodes in the network, exactly as in Figure 3A except that nodes are colored based on their new AnTE-based subfamily assignments. B) As in Figure 4A, all TEs in the data are added to the network as nodes, represented by small dots, and using the coloring scheme of the new AnTE-based subfamily assignments.

**Figure 5:**
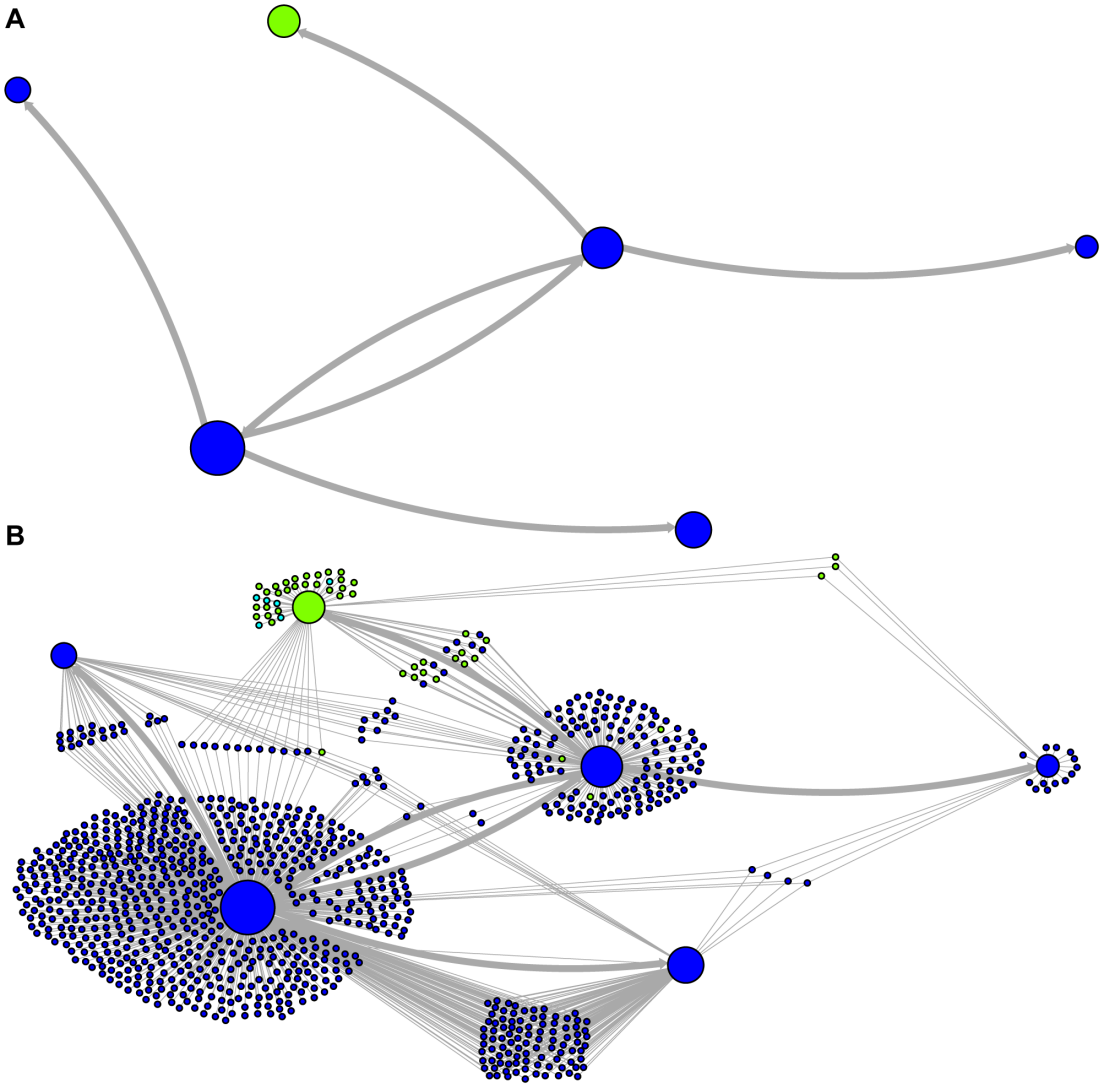
Ancestry networks of *Alu*Sc sequences. The predicted network of *Alu*Sc ancestral relationships is shown. A) All sequences that replicated with probability >30% are represented as nodes in the network. Arrows are drawn between sequences if there was at least 5% probability that an ancestral relationship existed between those sequences, with the direction of the ancestor-descendant relationships indicated by the arrows. Sequences are colored based on their CoSeg subfamily assignments. B) The network in A is modified by the addition of all extant TEs in the data added to the network as nodes represented by small dots. Edges are drawn between an element and an ancestral sequence if there was at least 5% probability the element descended from the ancestral sequence. Nodes are colored based on CoSeg subfamily assignment.

Network representations of the relationships among the elements of the LAVA and *Alu*Sc families are shown in Figures 3–5. These networks show the predicted ancestral relationships among all sequences with more than 50% probability of being replicative (shown most clearly in Figure 3a and 5a). The arrows on the graph indicate the predicted original source of each replicative sequence, with cycles representing uncertainty about the direction of original descendancy. Note that later copies of that sequence may have arisen from other ancestors, including possible back mutation from one of its descendants. Each node in the graph represents a particular sequence, with the diameter of the node proportional to its estimated frequency of replication.

There are four sequences inferred to have at least a 5% probability of being the LAVA root according to the AnTE algorithm. We compared these sequences to the segment of the human genome homologous to the 3′ end of LAVA [19]. One of these four plausible root sequences (Figure 3 and 4, marked with an arrow) has only 2 differences from the human sequence among 73 discriminatory sites; among all other candidates with >50% probability of being replicative, there are 4–28 differences (mean 12.1). Thus, the marked sequence is the probable ancestral LAVA, and the inferred root from AnTE is consistent with the homology data.

### Revised LAVA subfamilies

The assignment of CoSeg subfamilies to nodes in the ancestry networks of LAVA (Figure 3) and *Alu*Sc (Figure 5) indicates that most CoSeg subfamilies are represented by multiple ancestral replicative sequences. Although CoSeg subfamilies tend to cluster together in the network, replicative sequences from three LAVA subfamilies (colored in, purple, magenta and light blue in the graph) are disjointed, with intervening replicative sequences from other subfamilies (or that are not assigned to a subfamily at all). Additional discrepancies can be found when considering the CoSeg subfamily assignments of all sequences, not just replicative sequences (Figure 3b). Among descendants of all ancestors with CoSeg subfamily assignment, 57 LAVA sequences (6.5%) and 19 *Alu* sequences (2%) are assigned to different subfamilies than their most probable ancestor.

Based on this result, and considering the ancestral relationships inferred by the AnTE MCMC, we propose a subfamily organization for LAVA with 9 new subfamilies (Figure 4; see Figure S1 for legend). This subfamily scheme was designed based on the desiderata of a) relatively few subfamilies; 2) matching the CoSeg subfamilies where possible, to facilitate comparison; and 3) minimizing the number of sequences with uncertain subfamily assignment. The low mixing of colors in Figure 4b indicates that we have largely achieved our goal, although there is unavoidable uncertainty at most boundaries between subfamily groups. We want to emphasize here that the utility of the subfamilies is entirely organizational and aesthetic. We recommend that any analytical inference be carried out on the full ancestral probability network, and that it should sum over all ancestral uncertainty rather than arbitrarily assigning uncertain sequences to one ancestor or another and subsequently treating the assignment as though it were data.

### Many discriminatory sites are used multiple times in LAVA

We estimated the number and rate of substitutions between ancestral and descendent sequences at each site. This analysis indicates that, contrary to the assumption of the CoSeg algorithm, substitutions at individual sites repeatedly discriminate among replicative sequences. In LAVA, there are multiple substitutions among replicative sequences at between 38%–45% (95% credible region) of the discriminatory sites.

The CoSeg algorithm does not allow sites to discriminate between subfamilies more than once; this is intended to prevent the creation of new subfamilies from elements formed by recombination between sequences from separate subfamilies. However, it is reasonable to expect that substitutions that create new replicative sequences may occur multiple times. From a mechanistic perspective, discriminatory sites may be less likely to affect replicative function, whereas non-discriminatory sites may be more likely to affect replicative function. If there are only a limited number of sites that don't affect function, all of the mutations among replicative sequences will be focused on those sites. To test whether all sites are equally likely to be discriminatory, we considered a null model in which the probability of substitution between ancestral replicative sequences is proportional to the probability of substitution to extant sequences at that site. We randomly re-sampled all substitutions on the tree of replicative LAVA sequences to obtain a null distribution for the number of substitutions per site. Although 33–42 sites (MCMC 95% credible region) had exactly one substitution among the actual replicative sequences, 51–93 sites had a single substitution in 1000 draws of the null model. Thus, there is an excess of sites with multiple substitutions among ancestors in the observed data compared to the null hypothesis of no constraint. We conclude that, as expected, some variants are not neutral with regard to replication.

To further explore this question, we created a simple model of constraint on replicative elements that allows for two types of sites: constrained sites, which eliminate replicative capacity entirely if mutated, and unconstrained sites, which have no effect on replicative capacity. We tested this model for different *m*, the number of constrained sites among the 330 sites analyzed (microsatellites, CpG sites, and large insertions were removed prior to MCMC analysis and therefore were not considered). As before, substitutions were drawn to match the number among replicative sequences, but no substitution was allowed at *m* random sites separately selected for each draw. Taking the upper bound inference of 42 sites with single substitutions, the lowest *m* for which at least 5% of 1000 draws had 42 or fewer sites with one substitutions was 163, leaving 167 sites unconstrained. This analysis suggests that only around half the tested sites are effectively neutral to replicative function.

### Analysis of 5′ region of LAVA

The LAVA sequence is divided by a VNTR (variable number of tandem repeats) region of up to 2000 bp. Our main analysis focused on the region 3′ from the VNTR, as many LAVA loci lack all or part of the VNTR and 5′ region. The full-length 5′ region is around 350 bp, and we found 337 loci with intact 5′ regions. Analysis of these sequences revealed three separate clusters defined by presence or absence of two large interior segments of around 100 bp each. We used AnTE to reconstruct the ancestral relationships separately within each of these three clusters. These ancestral networks largely agree with the analysis of the 3′ region: the first cluster consists mostly of sequences from the adjacent green, purple, and brown subfamilies from Figure 4 (Figure S2A); the second cluster consists mostly of green and grey subfamilies (Figure S2B), and the third cluster is composed mostly of the older red, yellow, pink, and blue subfamilies (Figure S2C). However, 26 sequences (7.7%) are assigned ancestors on the 5′ network that are distantly related to ancestors in the 3′ network. A probable explanation for this discrepancy in placement between the 3′ and 5′ ancestral networks is recombination across the VNTR. Aside from these putative recombinants, the network structure within the three 5′ clusters is largely in agreement with the structure of the 3′ network (compare Figure 4 and Figure S2).

### Validation of AluSc ancestry network using rhesus macaque homologues to human elements

Ancestral inference of TEs that inserted prior to a speciation event can be validated by comparing homologous elements between two species. To see this, consider that if the ancestor is correct, then the number of shared differences from the ancestor at each site will be approximately proportional to the time between insertion and speciation (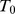). The number of unique differences in each branch will be approximately proportional to the time between speciation and the present (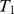). Sequences that differ from the predicted ancestor upon insertion will have an inflated number of shared differences from the predicted ancestor. This will lead to a higher estimate of 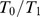 than at non-discriminatory sites.

Taking the *Alu*Sc consensus sequence as the presumed ancestor, we found that five of the six discriminatory sites inferred by our method exceeded the mean 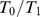 ratio by 3-fold or greater (Figure S3), whereas all of the non-discriminatory sites have lower ratios. To validate each branch on the tree in Figure 5b, we separately considered the descendants of each predicted ancestral sequence (the “test” ancestor) along with all of the descendants of its ancestor (the “parent”). Considering the 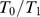 ratios assuming the parent sequence was the true ancestral sequence, a positive validation result would consist of a high ratio (exceeding the 3x threshold) for the site that discriminated the test ancestor from the parent. All predicted ancestors were validated by this test. No non-discriminatory sites exceeded the 3-fold threshold except a single CpG site (position 1), which is possibly a true discriminatory site that was undetected because we eliminated CpG sites in the AnTE analysis. It is also notable that in this branch-validation analysis, the discriminatory site with the lowest ratio in the overall consensus analysis (Figure S3) was validated, but the two non-discriminatory sites that had higher ratios were not.

## Discussion

We have confirmed here that the CoSeg subfamily classification method fails to identify many highly-probable ancestral sequences in both LAVA and *Alu*Sc, and therefore that CoSeg subfamily consensus sequences are problematic for use as presumed ancestors in divergence and substitution analysis. In contrast, the AnTE method we developed and describe here provides a detailed picture of TE evolutionary history, providing ancestral sequences, the times of replicative activity of these sequences, and their replication frequency. The AnTE method is fast and enables the probabilistic evaluation of relationships between thousands of elements within subfamilies and between subfamilies. The AnTE program, relevant datasets, and user instructions are available at www.EvolutionaryGenomics.com.

Though the AnTE method identifies more sequences than previous approaches in both subfamilies studied, many more ancestrally-replicative sequences were identified for LAVA (50–60) than for *Alu*Sc (6–7) from similar-sized sequence datasets. Our analysis suggests that most *Alu*Sc sequences derive from a single ancestor, while the most successful LAVA source sequence is responsible for only 13% of extant LAVA elements. The two datasets are not directly comparable, as most of the LAVA sequences identified in the Gibbon genome were used for our analysis of LAVA, while only a small subset of *Alu*Sc was used, and *Alu*Sc itself is a subfamily of the much larger *Alu* TE family. Nevertheless, this large difference between families suggests differing evolutionary dynamics.

The method presented in this paper has some limitations that should be addressed in future work. Firstly, it assumes that all differences between sequences and their ancestors are the result of mutation, rather than recombination or gene conversion. We found strong evidence of recombination across the large VNTR region in LAVA in 7.7% of full sequences, but no obvious evidence of recombination between distant ancestral sequences within the regions either 5′ or 3′ from the VNTR. However, we cannot rule out the possibility that some sequences are a result of recombination events between closely-related subfamilies. Second, our method, like most phylogenetic methods, assumes site-independence. We excluded CpG sites from our analysis because their elevated mutation rate violates site independence. CpG sites are common in both LAVA and *Alu*, and it is possible that some are discriminatory sites that can help distinguish true ancestral sequences. Methods that allow the relaxation of site-independence assumptions would also allow large deletions and microsatellites to be used as TE subfamily markers. Here, we had to analyze the clusters separated by large deletions in independent analyses. Third, our method accounts for the activity periods of transposable elements in a simplistic way, assuming a single time point of activity rather than representing a distribution of replication rates across time. One obvious but non-trivial improvement that could be made would be to better estimate the distribution of replication times for each ancestral subfamily, such as has been done for *Alu* subfamilies [26].

Despite the assumptions made in creating subfamilies using previous approaches, they have often been used in studies of TE evolution. For example, most methods for estimating the age of subfamilies are based on some measure of divergence between subfamily consensus sequences and the members of the subfamily [27]–[30]. Our findings suggest that this prior widespread use of subfamily consensus sequences as the single ancestral subfamily source sequence to analyze TE mutation patterns [14] has led to over-estimation of substitution rates and TE divergence times, and to incorrect inference of substitution patterns. AnTE can be used to improve such analyses, and may be useful to revise existing subfamily nomenclature based on more realistic estimates of ancestral replication patterns, as we have done with the gibbon LAVA elements. Overall, we expect that such approaches will be central for evaluating genome structural evolution and using TEs to understand genome-wide mutation processes.

## Methods

### AluSc sequence filtering and alignment

The human genome was downloaded from the RepeatMasker [25] website. The 2006 build of the human genome was masked based on Repbase [31] version 20090604 using version RepeatMaskerOpen-3.2.8 of RepeatMasker. The annotated *Alu* sequences were extracted from the genome and sorted by subfamily classification. A total of 34,515 *Alu*Sc sequences were identified in this way. Of these, 1200 were selected at random for ancestry determination and manually aligned.

For all human *Alu* elements, the corresponding *Alu* elements from rhesus macaque were obtained using Galaxy [32]. The “extract pair wise MAF blocks” tool from Galaxy was used to get the sequence matches of each of macaque to human *Alu* elements. The “Stitch MAF blocks” tool was used to obtain the correspondence between matches among genomes to the human *Alu* coordinates. To ensure accurate alignment, macaque *Alu*Sc sequences with less than 80% identity to their human homologue were removed from analysis.

### Gibbon LAVA sequence filtering and alignment

We identified LAVA sequences in the Gibbon genome using the probability-based oligonucleotide clustering method *P-clouds* [33]. The published LAVA consensus sequence, which contains only the region 3′ of the VNTR [19], was segmented into regions which were used to form clouds. We then searched the genome for locations that matched the cloud data. Identified locations were merged if the distance between them was less than the length of the region in the consensus sequence. This resulted in 1136 sequences will full 3′ regions. Sequence for the region 5′ of the VNTR was obtained by building clouds from the region upstream of the VNTR in these sequences. Locations matching these clouds were then merged to the 5′ sequences to obtain full length sequences. This process identified 338 sequences with complete 5′ regions. Alignments for both the 3′ and 5′ regions were constructed manually.

### Sequence processing

An assumption of our model is that the substitution process at each site is independent of all other sites. This assumption is clearly violated by large insertions/deletions, CpG sites, and microsatellites. Therefore, CpG and gap sites within the consensus, as well as microsatellite regions, were excluded from all analyses. All sequences with gaps larger than four nucleotides in their alignment to the consensus were also excluded from analysis. This left 986 LAVA and 972 *Alu*Sc sequences for the main analysis. Alignments before and after processing are provided in Supplementary Data Files S1–S10.

### Identifying discriminatory sites and candidate ancestral element sequences

We define “discriminatory sites” as those sites which differ among historically replicative sequences. Since only discriminatory sites are informative in ancestry determination, our first goal is to predict these sites. Two features distinguish discriminatory from non-discriminatory sites. First, discriminatory sites will tend to have a higher frequency of a particular variant than expected by mutation alone. At non-discriminatory sites, all variation is due to substitution; at discriminatory sites, replication of a sequence that already differs at that position will also increase the frequency of the variant. Second, discriminatory sites will show association with each other, because discriminatory variants arise in particular backgrounds of variation at other discriminatory sites.

We predicted which sites are discriminatory as follows: First, a nucleotide substitution probability matrix 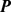 was derived by counting the number of differences from the consensus sequence 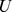 to each of the 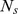 elements in the sequence database 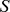. Each nucleotide difference count 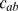 between each pair of bases or gaps, was used to obtain relative substitution probabilities from 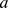 to 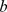,
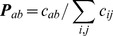
(1)


Sites with mutations exceeding the mean rate of any type of mutation by more than three standard deviations were then identified as an initial set of predicted discriminatory sites. For each predicted discriminatory site, we then tested for association with all other sites using a Monte Carlo chi-square test. All sites with p-values <.01 for association with any of the initially-predicted site were added to the pool of discriminatory sites. Note that, as described below, each candidate ancestral sequence is evaluated by MCMC for the probability it is a true ancestor. Therefore, we are not concerned with including some false discriminatory sites, as the strength of evidence for each site will be reflected in the final results.

A set of candidate ancestral sequences was constructed based on the predicted discriminatory sites. By definition, ancestral sequences do not differ at non-discriminatory sites, so all ancestors were assumed to agree with the consensus except at discriminatory sites. For *Alu*Sc, the small number of discriminatory sites allowed inclusion of all possible discriminatory site combinations as ancestral sequences. For LAVA, all discriminatory site combinations observed in the data were included as an initial set of candidate ancestral sequences. Since some ancestors may have had combinations of discriminatory site which no longer exist, we added new plausible candidates during the burn-in phase of the MCMC, as described below.

### The ancestry model

The TE ancestry model consists of three sets of parameters: 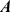, the replicative frequency of each candidate ancestor; 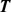, the estimated time of replicative activity of each ancestor, and rate parameters for a nucleotide substitution rate matrix 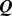. The ancestral frequencies were modeled as discrete variables with constant sum equal to the total number of sequences in the data. The parameters 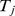 approximate the time of replicative activity for each candidate 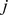 as a single time point, in which that candidate produced all descendants. For computational efficiency, these time parameters were restricted to 1001 equally-spaced points between 0 and 1, with 0 defined as the present and 1 as the time of activity of the root sequence. Flat priors were assumed for all parameters except 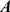, for which a penalty is applied for each candidate with nonzero replicative frequency. The size of this penalty was varied across runs to reflect different beliefs about the prior probability any given sequence is replicative. The likelihood of generating any TE sequence 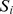 in the dataset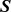, given all parameters, is defined as: 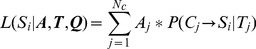
(2)where 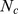 is the number of ancestral candidates, 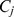 is the *j*th candidate ancestral sequence, 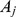 is the replicative frequency of candidate *j*, and 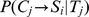 is the probability of transitioning from sequence 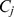
to sequence 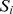 in time period 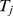. This sequence transition probability is the product of the transition probabilities at each site between the base in 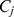 and the base in 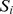 at that site. The transition probabilities between each pair of nucleotides over time 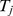 are obtained from the matrix exponential 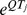.

The overall likelihood of the data, 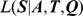, is the product of the likelihood of all sequences which exist according to these parameters, both current and ancestral. Note that for any *i* such that 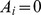, there is no implication that candidate 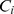 ever existed, so we need only consider the likelihood all candidate ancestors *i* for which 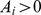. For any such sequence, other than the root of the family:
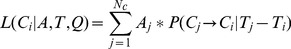
(3)


For 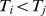, the sequence transition probability 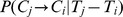 is the probability of transitioning from sequence 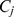 to sequence 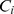 over time period 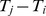, calculated, as described above, by taking the product of nucleotide transition probabilities over all sites. For 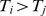, this probability is zero, since ancestral sequences cannot produce descendants which were active earlier than they were. The root sequence is defined to have likelihood 1.

The substitution rate matrix 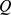 is defined by 10 rate parameters according to a general strand-symmetric model, giving the substitution rates between all pairs of nucleotides and single-nucleotide insertion/deletions.

### Details of the Markov chain analysis

The Markov chain was run using the Metropolis-Hastings method [34] to sample all parameters. The chain was initialized by randomly selecting half of the candidate sequences as replicative, and their initial frequencies were assigned according to a multinomial distribution with equal prior probabilities for each selected candidate. Two types of proposals were used to efficiently sample 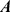, the replicative frequencies of the candidate ancestors. In the first proposal type, two candidate ancestors are selected at random; the frequency of the first is increased by one and the frequency of the second is reduced by one. Proposals are always rejected if acceptance would lead to negative values. In the second proposal type, the frequency of two randomly-chosen candidate ancestors is swapped.

The 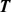 parameters were also sampled by two proposal types. In the first, a candidate ancestor *j* was selected at random. A random integer *n* was drawn from 0 to 1000, and 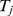 is set to n/1000. In the second, candidate ancestors 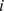 and 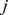 were selected at random, and their associated parameters 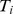 and 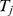 were swapped. The substitution rate parameters were sampled by a single proposal, in which the current rate was added to a draw from a normal distribution with mean 0 and standard deviation .01.

As all proposals are symmetric, the chain satisfies detailed balance if the acceptance probability 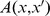 for the moves from 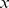 to 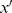 follows the Metropolis-Hastings [34] acceptance proposal, where 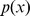 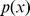 is the likelihood of the set of all parameters 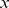. 
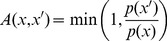
(4)


The first 10 million generations of the Markov chain were considered a burn-in stage, used to obtain an equilibrium sample of parameters prior to sampling the posterior. For the LAVA sequences, this stage was also used to add plausible candidates to the pool of candidate ancestral sequences for inclusion in the model. The initial set of candidate ancestral sequences was the set of discriminatory site sequences observed in the data. However, it was necessary to account for the possibility that some ancestral sequences were not represented; for every candidate sequence in the pool at any sampling point, every possible nucleotide change in the sequence was evaluated for whether the overall likelihood of the data would increase if that change were made, keeping everything else constant. If the likelihood increased for a given nucleotide change, a new candidate sequence, differing only by that nucleotide change, was added to the ancestral pool. New candidates were tested for addition every 100,000 steps from step 2.5 million to step 7.5 million in the burn-in.

After burn-in, the Markov chains were run for 10 million generations and sampled every 10,000 generations. Good mixing was verified by running three replicates with each replicate starting from a random parameter set, and confirming that the within-replicate variance was at least 99% of the overall variance.

### Validating the number of ancestral replicating sequences

Given a proposed ancestral reconstruction for a set of TE sequences, we developed a test of the hypothesis that mutation alone can explain the variation between descendants and their proposed ancestors. If the mutation hypothesis is true, we expect the substitution process at a given site to be independent of the ancestral sequence once the ancestral nucleotide and the site position are accounted for. Therefore, we can reject the mutation-only hypothesis if the descendants of a proposed ancestor have a higher frequency of a variant than can be explained by mutation alone. Such a result suggests the existence of one or more intermediate sequences between the ancestor and some of its proposed descendants that vary from the proposed ancestor at the high-frequency variant sites.

The basis of the test is to “redraw” the substitutions of each sequence in the data. First, the number of substitutions of each type at each site between all proposed ancestors and descendants were counted. For each sequence 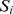 in the data, a new sequence 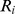 was constructed from its proposed ancestral sequence 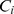 by adding a number of substitutions equal to the number of differences between 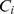 and 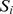. These substitutions were drawn randomly according to the following process. First, a site is selected for substitution. The probability of selecting any site 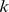 which was in nucleotide state 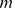 in 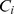 is weighted by the fraction of sequences which has a substitution at site 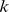 from an ancestor in state 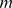 out of all sequences whose ancestor was in state 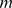 at site 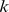. Then, the particular substitution is selected, with the probability of each substitution type weighted by the frequency of that substitution from the ancestral nucleotide 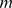 at that site according to the proposed ancestral reconstruction. This process is repeated until 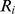 has a number of differences from 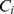 equal to the number of differences between 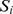 and 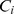. Note that this redraw process accounts for differences in substitution probability at a site based on ancestral nucleotide at that site and position.

The redraw process is conducted 1000 times. For each redraw, a 3-dimensional matrix is constructed giving the number of each variant at each site among descendants of each ancestor. The entries in these matrices are averaged among redraws to give a matrix of expected values. For each redraw 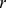, the sum 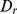 of absolute differences between observed and expected values is computed over the entire matrix. Finally, the sum 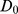 is computed by the same calculation based on the actual substitutions according to the proposed ancestral reconstruction. If the mutation hypothesis is true, 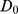 should fall within the distribution of the 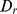 values.

This redraw test was run on both the CoSeg-inferred ancestors and the ancestors inferred from the AnTE algorithm. To draw a deterministic ancestral reconstruction from the probabilistic output of AnTE, a step of the MCMC after convergence was selected at random, and all sequences were assigned ancestors based on their probability of descendance according to the parameters at that step.

### Models of replicative sequence constraint

A relevant question in understanding TE ancestry is whether only a limited number of sequences can be successful in the replication process. If so, it is expected that mutations at constrained sites will lead to inactive copies that will not replicate further. Such sites will be non-discriminatory, while sites that do allow substitutions among ancestral replicators may become discriminatory sites. To assess whether there was support for constraint at some sites, we tested whether the substitution patterns matched either of two models of sequence constraint in replicative TEs. In the null model, no constraint was assumed, so the expected relative frequency of substitutions at a site among replicative elements equaled the relative frequency overall. In the test model, it was assumed that *m* sites were completely constrained, so that any differences from consensus at that site prevented replication.

A random tree of ancestral relationships was drawn from the MCMC data by selecting a step of the MCMC at random, and assigning ancestors to all data sequences and inferred ancestral sequences randomly, with the probability of assignment to each ancestor weighted by the probability of descendence from that ancestor according to the parameters at that step. As this tree gives the ancestral sequence for all sequences in the data, we can use it to derive the substitutions between ancestors and descendants, distinguishing between substitutions to replicative and non-replicative sequences.

The test statistic 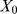 was the number of sites with no substitutions among replicative sequences; i.e., the number of discriminatory sites. We generated distributions of 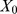 according to the assumptions of each model, and then compared these to the posterior distribution of 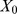 implied by the MCMC results. First, 1000 trees of TE relationships were drawn randomly. For each tree, the number of substitutions at each site was calculated, both for all elements and restricting to replicative elements. Additionally, the number of sites with no substitutions was calculated to get the distribution of 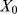 according to the MCMC results. Then, to generate a distribution of 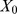 according to each model, for each tree we drew from a multinomial distribution with number of trials equal to the total number of substitutions among replicative elements according to that tree. For the first model, the vector of probabilities in the multinomial distribution is the relative frequency of substitution at each site. For the second model, 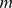 sites were selected from the sites for which no substitutions occurred among replicative elements according to the tree. These sites were assigned a substitution probability of zero, and the other probabilities were normalized to sum to 1 before drawing from the multinomial distribution. Thus, from 1000 draws of a tree, we obtain distributions of 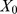 according to the MCMC results, the no-constraint model, and models for each possible value of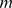, from 1 to the total number of sites. We reject a model if fewer than 5% of 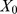 values fell within the 95% confidence region for 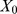 from the MCMC. The best fit 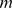 for the second model was defined as the 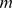 that minimized the absolute difference of the ordered 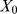 values from the MCMC and the model.

### Validation of *Alu*Sc ancestry relationships using homologous macaque sequences

The *Alu*Sc subfamily predates the split between human and rhesus macaque. We used the homologous *Alu*Sc sequence to validate the ancestors inferred by AnTE. We define 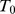 as the time between insertion of an *Alu*Sc sequence and the split between macaque and human, and 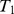 as the time between the split and the present. Given that the ancestral nucleotide at a position is 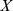, we can estimate the probability that neither, one, or both of the macaque and human sequence have substituted away from 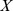. Assuming low rates of substitution, and no back-mutation, the probability of substitution is approximately proportional to time. The probability that both descendant sequences are still 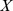 is then:
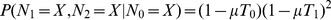
(5)where 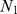 and 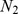 are the present-day bases in human and macaque, respectively, 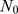 is the base the TE has upon insertion, and 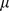 is the mutation rate. Similarly, the probability that one of the two descendant sequences has substituted away is:
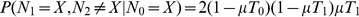
(6)


By inserting the proportion of sequences with 0 or 1 substitutions into the above equations and solving for 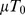 and 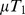, we can obtain an estimate for 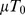 and 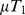 at every site, under the hypothesis that all sequences are descended from the consensus. Though we expect 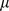 to differ between sites, estimates of the ratio 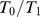 should be similar if the hypothesis holds. If the hypothesis is false, then at sites where some of the sequences already differed from the consensus when they were inserted, we expect estimates of this ratio to be higher than at other positions, to account for the greater number of sequences for which macaque and human share a difference from the consensus. Thus, a relatively high estimate 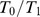 indicates a discriminatory site.

Given a tree of relationships among *Alu*Sc sequences, we estimate 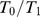 for every position among all descendants (immediate or distant) of each ancestor. We consider a branch in the tree validated if the sites which distinguish the descendant node from the ancestral node all have 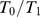 ratios at least 3-fold greater than the mean ratio.

## Supporting Information

Figure S1 Subfamily color legend. Subfamilies as defined by CoSeg are shown divided into two groups: those that correspond to a new AnTE subfamily (shared subfamilies #1–9), and those which are not classified as AnTE subfamilies (ancestral CoSeg-only subfamilies #10–14). The subfamily colors correspond to coloration in the main figures, and numbering corresponds to information in the tables.
(TIF)

Figure S2 LAVA ancestry network based on 5′ region. The predicted network of LAVA ancestry relationships, as described in Figure 4, but based on the region 5′ of the VNTR rather than the 3′ region. A) Cluster 1 network B) Cluster 2 network C) Cluster 3 network. Colors of sequences are based on the subfamily assignments shown in Figure 4.
(TIF)

Figure S3 T_0_/T_1_ ratios for all sites, assuming *Alu*Sc consensus is ancestral. Estimated **T_0_/T_1_** ratios are plotted for every position, assuming that *Alu*Sc is ancestral to all sequences in the data. The two horizontal lines are the mean ratio and 3x the mean ratio. Sites are categorized based on whether they are discriminatory and whether they are CpG sites.
(TIF)

Table S1 Discriminatory site sequence for all LAVA candidate ancestors.
(DOCX)

Table S2 MCMC results for LAVA candidate ancestors.
(DOCX)

Table S3 MCMC results for *AluSc* candidate ancestors.
(DOCX)

File S1 Unprocessed alignment files for LAVA 3′ region sequences. Aligned sequences from the 3′ end of LAVA, before processing.
(FASTA)

File S2 Processed alignment files for LAVA 3′ region sequences. Aligned sequences from the 3′ end of LAVA, after processing, as described in methods.
(FASTA)

File S3 Unprocessed alignment files for LAVA 5′ cluster 1 sequences. Aligned sequences from cluster 1 in the 5′ end of LAVA, before processing.
(FASTA)

File S4 Processed alignment files for LAVA 5′ cluster 1 sequences. Aligned sequences from cluster 1 in the 5′ end of LAVA, after processing.
(FASTA)

File S5 Unprocessed alignment files for LAVA 5′ cluster 2 sequences. Aligned sequences from cluster 2 in the 5′ end of LAVA, before processing.
(FASTA)

File S6 Processed alignment files for LAVA 5′ cluster 2 sequences. Aligned sequences from cluster 2 in the 5′ end of LAVA, after processing.
(FASTA)

File S7 Unprocessed alignment files for LAVA 5′ cluster 3 sequences. Aligned sequences from cluster 3 in the 5′ end of LAVA, before processing.
(FASTA)

File S8 Processed alignment files for LAVA 5′ cluster 3 sequences. Aligned sequences from cluster 3 in the 5′ end of LAVA, after processing.
(FASTA)

File S9 Unprocessed alignment files for *AluSc* sequences. Aligned sequences from *AluSc*, before processing.
(FASTA)

File S10 Processed alignment files for *AluSc* sequences. Aligned sequences from the 3′ end of LAVA, after processing.
(FASTA)
