# Supplementary figures and images for: Inference of Transposable Element Ancestry
Source: PLoS Genet. 2014 Aug 14;10(8):e1004482. doi: 10.1371/journal.pgen.1004482 (PMC4133154; doi:10.1371/journal.pgen.1004482)

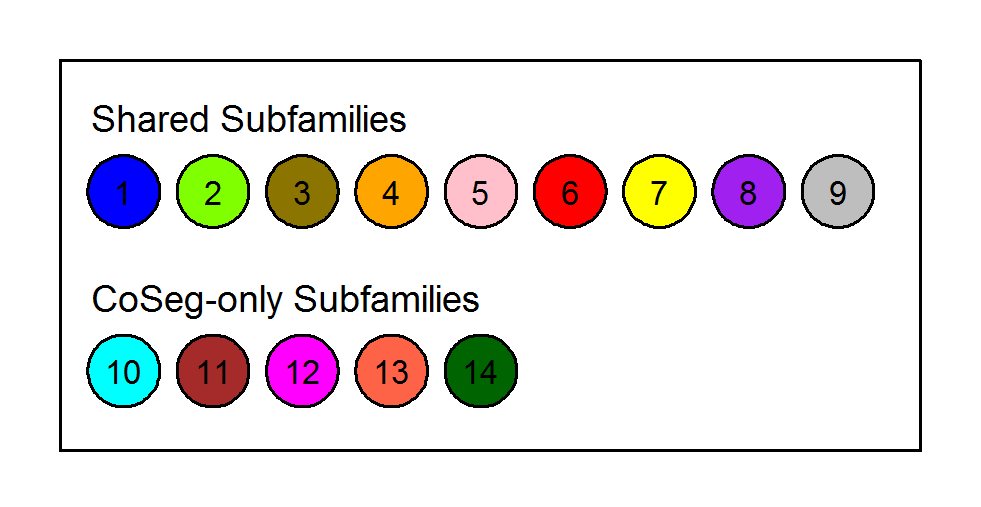

Supplement: Figure S1 — Subfamily color legend. Subfamilies as defined by CoSeg are shown divided into two groups: those that correspond to a new AnTE subfamily (shared subfamilies #1–9), and those which are not classified as AnTE subfamilies (ancestral CoSeg-only subfamilies #10–14). The subfamily colors correspond to coloration in the main figures, and numbering corresponds to information in the tables. (TIF) [file pgen.1004482.s001.tif]

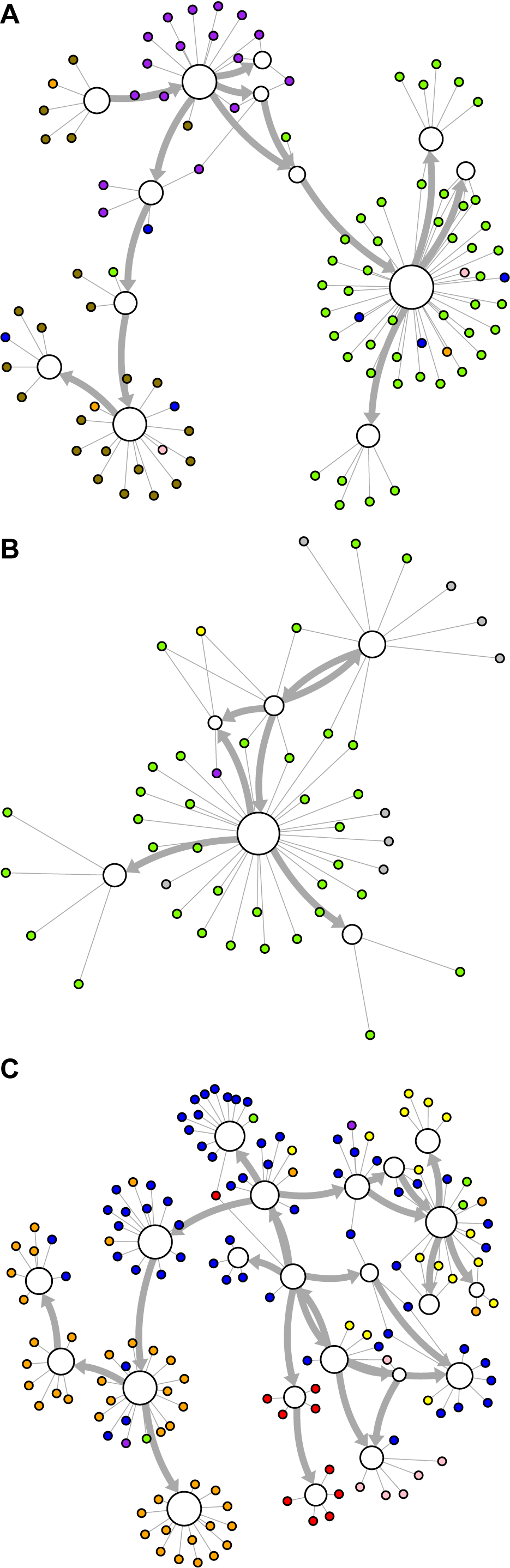

Supplement: Figure S2 — LAVA ancestry network based on 5′ region. The predicted network of LAVA ancestry relationships, as described in Figure 4, but based on the region 5′ of the VNTR rather than the 3′ region. A) Cluster 1 network B) Cluster 2 network C) Cluster 3 network. Colors of sequences are based on the subfamily assignments shown in Figure 4. (TIF) [file pgen.1004482.s002.tif]

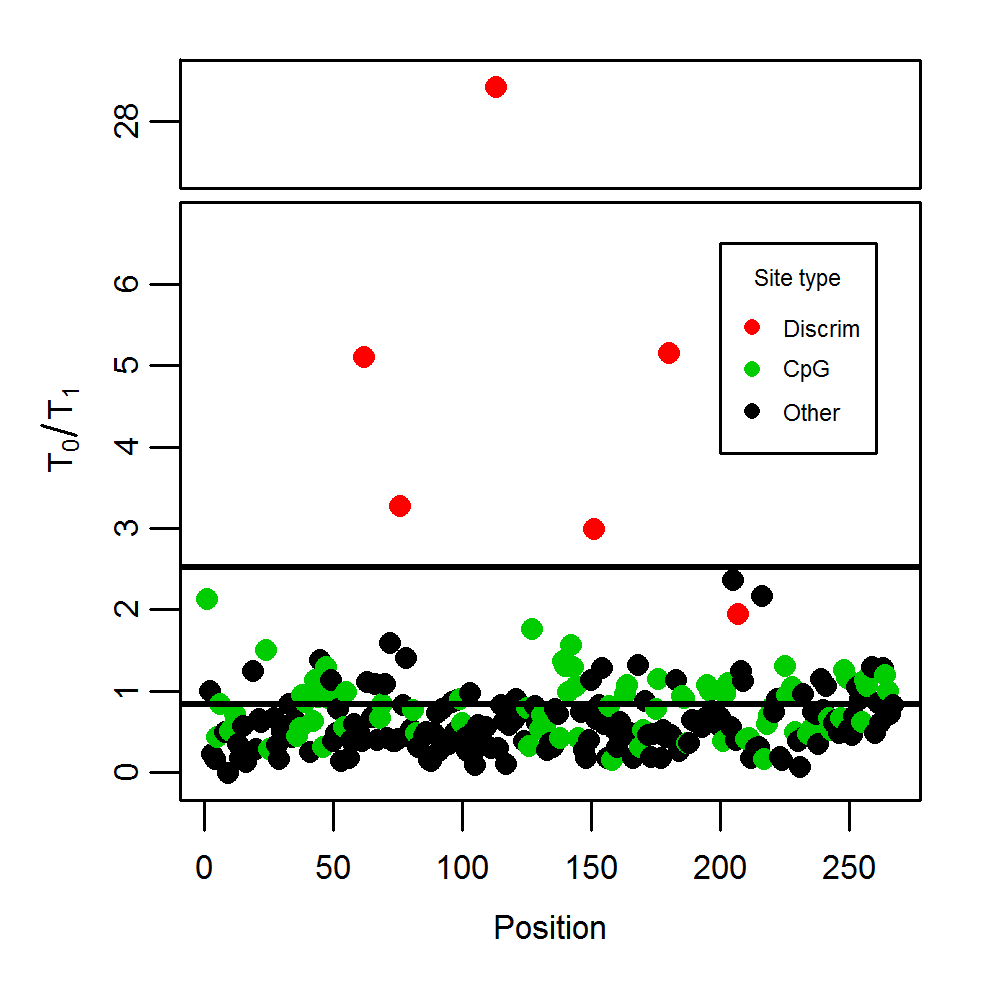

Supplement: Figure S3 — T0/T1 ratios for all sites, assuming AluSc consensus is ancestral. Estimated T0/T1 ratios are plotted for every position, assuming that AluSc is ancestral to all sequences in the data. The two horizontal lines are the mean ratio and 3x the mean ratio. Sites are categorized based on whether they are discriminatory and whether they are CpG sites. (TIF) [file pgen.1004482.s003.tif]
