# Supplementary material for: Inference of Transposable Element Ancestry
Source: PLoS Genet. 2014 Aug 14;10(8):e1004482. doi: 10.1371/journal.pgen.1004482 (PMC4133154; doi:10.1371/journal.pgen.1004482)
Supplement: Table S1 — Discriminatory site sequence for all LAVA candidate ancestors. (DOCX) [file pgen.1004482.s004.docx]

| Replicative frequency rank | Candidate sequence |
| --- | --- |
| 1 | AGGACGAGGAGTCGAGTGCTA--ACAGAATGCAAACGTAAATCAATAAAACCTACCTAACCCCCCATATATAA |
| 2 | AGGACGAGGAGTCGAGTGCTA--ACAGAATGCAAACGTAAATCAATAAAACTTCATTAACCCCCCATATATAA |
| 3 | AGGACAAGAAGTGAAGTGCAAATACTGAACGCAAACGTAAATCAATAAAATTCCATTAATCCCCCATTTATAA |
| 4 | AGGACAAGAAGTGAAGTGCAAATACAGAACGCAAACGTAAATCAATAAAATTCCATTAATCCCCCATTTATAA |
| 5 | AGGACAAGAAGTGAAGCGCAAATACTGTACGCAAACGTAAATCAATAAAATTCCATTAATCCCCCATTTATAA |
| 6 | ACGACGAGGAGTCGAGTGCAAATACAGAATGCAAATGTAAATCAATAAAACTTCATTAGTCCCTCATTTATAA |
| 7 | AGGACAAGAAGTGAAGTGCAAATACAGAACACAAACGTAAATCAATAAAATTCCATTAATCCCCCATTTATAA |
| 8 | AGGACGAGGAGTCAAGTGCAAATACAGAATGCAAACGTAAATCAATAAAACTTCATTAATCCCCCATATATAA |
| 9 | AGGACAAGAAGTGAAGTGCAAATACAGAACGCAAACGTAAATCAAGAAAATTCCATTAATCCCCCATTTATAA |
| 10 | AGGACGAGGAGTCAAGTGCAA--ACAGAATGCAAACGTAAATCAATAAAACTTCATTAATCCCCCATATATAA |
| 11 | AGGACGAGAAGTGAAGTCCAAATACAGAATGCAAACGTAAATCAATAAAATTCCATTAATCCCCCATTTATAA |
| 12 | AGGACAGGAAGTCAAGTGCAAATACAGAACGCAAACGTAAATCAATAAAATTCCATCAATCCCTCATTTATAA |
| 13 | AGGACAAGAAGTGAAGTGCAAATACAGAACGCAAACGTAAATCAATAAAATTCCATTAATCCCTCATTTATAA |
| 14 | AGGACGAGGAGTCGAGTGCAA--ACAGAATGCAAACGTAAATCAATAAAACTTCATTAATCCCCCATATATAA |
| 15 | AGGACGAGGAGTCAAGTGCAAATACAGAATGCAAACGTAAATCAATAAAACTTCATTAATCCCTCATTTATAA |
| 16 | AGGACAAGAAGTGAAGTGCAAATACTGAACGCAAACGTGAATCAATAAAATTCCATTAATCCCCCATTGATAA |
| 17 | AGGACAAGAAGTGAAGTGCAA--ACAGAACGCAAACGTAAATCAATAAAATTCCATTAATCCCCCATTTATAA |
| 18 | AGGACGAGGAGTCGAGTGCTA--ACAGAATGCAAACGTAAATTAATAAAACCTACCTAACCCCCCATATATAA |
| 19 | AGGACAAGAAGTCAAGTGCAAATACAGAACGCAAACGTAAATCAATAAAATTCCATTAATCCCTCATTTATAA |
| 20 | GGGACAAGAAGTGAAGTGTAAATACAAAACACAAACGTAAATCAATAAAATTCCATTACTCCCCCATTTATAA |
| 21 | ACGACGAGGAGTCGAGTGCAGATGCAGAATGCGGGTGTAAATCAATAAAACTTCATTAGTCCCTCATTTGTGG |
| 22 | AGGACAAAAAGTGAAGTGCAAATATAGAACACAAACGTAAATCAATAAAATTCCATTAATCTCCCATTTATAA |
| 23 | AGGACAAAAAGTGAAGTGCAAATACAGAACACAAACGTAAATCAATAAAATTCCATTAATCCCCCATTTATAA |
| 24 | AGGACAAGAAGCGAAGTGCAAATACAGAACGCAAACGTAAATCAATAAAATTCCATTAATCCCTCATTTATAA |
| 25 | AGAACAGGAAGTCAAGTGCAAATACAGAACGCAAACGTAAATCAATAAAATTCCATCAATCCCTCATTTATAA |
| 26 | AGGACGAGGAGTCGAGTGCAAATACAGAATGCAAACGTAAATCAATAAAACTTCATTAATCCCCCATATATAA |
| 27 | AGGACGAGGAGTCGAGTGCAAATACAGAATGCAAACGTAAATCAATAAAACTTCATTAATCCCTCATTTATAA |
| 28 | GGGACAAGAAGTGAAGTGCAAATACA--ACACAAACGTAAATCAATAAAATTCCATTACTCCCCCATTTATAA |
| 29 | GGGACAAGAAGTGAAGTGCAAATACAAAACACAAACGTAAATCAATAAAATTCCATTACTCCCCCATTTATAA |
| 30 | AGGACGAGGAGTCGAGTGCTA--ACAGAATGCAAACGCAAATCAATAAAACTTCATTAACCCCCCATATATAA |
| 31 | AGGACAAGAAGTGAAGTGCAAATACAGAACACAAACGTAAATCAAGAAAATTCCATTAATCCCCCATTTATAA |
| 32 | AGGACGAGGAGTCGAGTGCTA--ACAGAATGCAAACGTAAAT-AATAAAACCTACCTAACCCCCCATATATAA |
| 33 | AGGACAAGAAGTGAAGTGCAAATACAG--CGCAAACGTAAATCAATAAAATTCCATTAATCCCCCATTTATAA |
| 34 | AGGACAAGAAGTGAGGTGCAAATACAGAACACAAACGTAAATCAATAAAATTCCATTAATCCCCCATTTATAA |
| 35 | AGGACAAGAAGTGGAGTGCAAATACAGAACGCAAACGTAAATCAATAAAATTCCATTAATCCCTCATTTATAA |
| 36 | AGGACAAGAAGTCGAGTGCAAATACAGAATGCAAACGTAAATCAATAAAATTTCATTAATCCCTCATTTATAA |
| 37 | AGGACAAGAAGTCAAGTGCAAATACAGAACGCAAACGTAAATCAATAAAATTTCATTAATCCCTCATTTATAA |
| 38 | AGGACAAGAAGTGAAGTGCAAATAC---GCGCAAACGTAAATCAATAAAATTCCATTAATCCCCCATTTATAA |
| 39 | AGGACGAGGAGTTGAGTGCAAATACAGAATGCAGGCGTAAGTCAGTGAGGCTTCATTGGTCCCTCATTTACAG |
| 40 | AGGACATGAAGTGAAGTGCAAATACAGAACACAAACGTAAATCAATAAAATTCCATTAATCCACCATTTATAA |
| 41 | AGGACAAAAAGTGAAGTGCAAATATAGAGCACAAACGTAAACCAGTAAAATTCCATTAATCTCCCATTTATAA |
| 42 | AGGACGAGGAGTCAGGTGCAAATACAGAATGCAAACGTAAATCAATAAAACTTCATTAGTCCCCCATATATAA |
| 43 | AGGACGAGGAGTCGACTGCTA--ACAGAATGCAAACGTAAATCAATAAAACCTACCTAACCCCCCATATATAA |
| 44 | AGGACGAGGAGTCGAGTGCTA--ACAGAATGCAAACGTAAATCAATAAAACTTCAT--ACCCCCCATATATAA |
| 45 | AGGACGAGGAGTCGAGTGCTA--ACAGAATGCAAGCGTAAATTGGTAGGACCTACCTAACCCCCCATATATAA |
| 46 | AGGACAAAAAGTGAAGTGCAAATACAGAACACAAACGTAAATCAATAAAATTCCATTAATCTCCCATTTATAA |
| 47 | AGGACAAGAAGTGA--TGCAAATACTGAACGCAAACGTAAATCAATAAAATTCCATTAATCCCCCATTTATAA |
| 48 | AGGTCGAGGAGTCGAGTGCTA--ACAGAATGCAAACGTAAATCAATAAAACCTACCTAACCCCCCATATATAA |
| 49 | AGGACAAGAAGTCAAGTGCAAATACAGAACGCAAACGTAAATCAATAAAACTTCATTAATCCCTCATTTATAA |
| 50 | AGGACGAGGAGTCGAGTGCTA--ACAGAATGCAAGCGTAAATTAATAAGACCTACCTAACCCCCCATATATAA |
| 51 | AGGACGAGGAGTCAAGTGCTA--ACAGAATGCAAACGTAAATCAATAAAACTTCATTAACCCCCCATATATAA |
| 52 | AGGACAAGAAGTCAAGTGCAAATACAGAATGCAAACGTAAATCAATAAAACTTCATTAATCCCTCATTTATAA |
| 53 | AGGACAAGAAGTGAAGTGCAA--ACTGAACGCAAACGTAAATCAATAAAATTCCATTAATCCCCCATTTATAA |
| 54 | AGGACGAGGAGTCGAGTGCTA--ACAGAATGCAAACGTAAATCAATAAAACCTACTTAACCCCCCATATATAA |
| 55 | GGGACAAGAAGTGAAGTGCAAATACAAAACACAAACGTAAATCAATAAAATTCCATTAATCCCCCATTTATAA |
| 56 | AGGACAAAAAGTGAAGTGCAAATACAGA-CACAAACGTAAATCAATAAAATTCCATTAATCTCCCATTTATAA |
| 57 | GGGACAAGAAATGAAGTGCAAATACAAAACACAAACGTAAATCAATAAAATTCCATTAATCCCCCATTTATAA |
| 58 | AGGACAAGAAGTGAAGTGCAA--ACAGAACGCAAACGTAAATCAATAAAATTCCATTAATCCCTCATTTATAA |
| 59 | AGGACGAGGAGTCGAGTGCTA--ACAGAATGCAAACGTAAATCAGTAAAACCTACCTAACCCCCCATATATAA |
| 60 | AGGACAAGAAGTGAAGTGTAAATAAAGAACATAAACGTAAATCAATAAAATTCCATTAATCCCCCATTTATAA |
| 61 | AGGACAAGAAGTGAAGTGCAAATACTGA-CGCAAACGTAAATCAATAAAATTCCATTAATCCCCCATTTATAA |
| 62 | AGGACAAGAAGTCAAGTGCAAATACAGAATGCAAACGTAAATCAATAAAATTTCATTAATCCCTCATTTATAA |
| 63 | AGGACAAGAAGTGAAGTGCAAATACTGAACGCAAACGTAAATCAATAAAATTCCATTAATCCCCCATTGATAA |
| 64 | AGGACGAGGAGTCGAGTGCTA--ACAGAATGCAGGCGTAAATTAATAAGACCTACCTAACCCCCCATATATAA |
| 65 | AGGACAAGAAGTGAAGTGCAAATACTGAACGCAAACGTGAATCAATAAAATTCCATTAATCCCCCATTTATAA |
| 66 | AGGACGAGGAGTCGAGTGCTA--ACACAATGCCAACGTAAATCAATAAAACTTCATTAACCCCCCATATATAA |
| 67 | AGGACGAGGAGTCGAGTGCTA--TCAGAATGCAAACGTAAATCAATAAAACTTCATTAACCCCCCATATATAA |
| 68 | AGGACGAGGAGTCAAGTGCAAATACAGAATGCAAACGTAAATCAATAAAACTTCATTAATCCCTCATTTACAA |
| 69 | AGGACGAGGAGTTGAGTGCTA--ACAGAATGCAAACGTAAATCAATAAAACTTCATTAACCCCCCATATATAA |
| 70 | ACGACGAGGAGTCGAGTGCAAATACAGAATGCAAATGTAAATCAATAAAACTTCATTAGTCCCTCATTTATTA |
| 71 | AGGACGAGGGGTCGAGTGCAA--ACAGAATGCAAACGTAAATCAATAAAACTTCATTAATCCCCCATATATAA |
| 72 | AGGACAAGAAGTCAAGTGCAA--ACAGAACGCAAACGTAAATCAATAAAATTCCATTAATCCCTCATTTATAA |
| 73 | AGGACGAGGAGTCGAGTGCTA--ACAGGATGCAAACGTAAATCAATAAAACCTACCTAACCCCCCATATATAA |
| 74 | AGGACGAGGAGTCGAGTGCTA--ACAGAATGCAAACGTAAATCGATAAAACTTCATTAACCCCCCATATATAA |
| 75 | AGGACAAGAAGTGAAGTGCAAATAC---GCACAAACGTAAATCAATAAAATTCCATTAATCCCCCATTTATAA |
| 76 | AGGACAAGAAGTGAAGTGCAAATACAGA-CGCAAACGTAAATCAATAAAATTCCATTAATCCCCCATTTATAA |
| 77 | AGGACGAGAAGTGAAGTCCAAATACAGAATGCAAACGTAAATCAATAAAATTCCATTAATCCCCCAATTATAA |
| 78 | AGGACGAGGAGTCAAGTGCAA--ACAGAATGCAAACGTAAATCAATAAAACTTCATTAATCCCTCATTTATAA |
| 79 | AGGATAAGAAGTGAAGTGCAAATACAGAACGCAAACGTAAATCAATAAAATTCCATTAATCCCCCATTTATAA |
| 80 | AGGACAAGAAGTGAGGCGCAAATACTGTACGCAAACGTAAATCAATAAAATTCCATTAATCCCCCATTTATAA |
| 81 | AGGACAAGAAGTGAAGTGCAAATACAGAACGCAAACGTAAATCGAGAAAATTCCATTAATCCCCCATTTATAA |
| 82 | AGGACAAGAAGTGAAGTGCAAATACAGAATGCAAACGTAAATCAATAAAATTCCATTAATCCCCCATTTATAA |
| 83 | AGGACAAGAAGTGAAGTGCAAATACAGAACGCAAACGTAAATCAATAAAATTCCATTAATCCCCCGTTTATAA |
| 84 | AGGACGAGGAGTCGAGTGCTA--ACAGAATGCAAACGTAAATCAATAAAACTTCATTAACCCCTCATATATAA |
| 85 | AGGACAAGAAGTGAAGTGCAAATACAGAACACAAACGTAAATCGATAAAATTCCATTAATCCCCCATTTATAA |
| 86 | AGGACAAGAAGTGAGGTGCAAATACAGAACGCAAACGTAAATCAATAAAATTCCATTAATCCCCCATTTATAA |
| 87 | AGGACAAGAAGTGAAGCGCAAATACAGAACGCAAACGTAAATCAATAAAATTCCATTAATCCCCCATTTATAA |
| 88 | AGGACAGGAAGTGAAGTGTAAATAAAGAACATAAACGTAAATCAATAAAATTCCATTAATCCCCCATTTATAA |
| 89 | AGGACGAGGAGTCGAGTGCTA--ACAGAATGCAAACGTAAATCAATAAAACTTCATT-ACCCCCCATATATAA |
| 90 | GGGACAAGAAGTGAAGTGCAA--ACA--ACACAAACGTAAATCAATAAAATTCCATTACTCCCCCATTTATAA |
| 91 | AGGACGAGGAGTCGAGTGCAAATACAGAATGCAAATGTAAATCAATAAAACTTCATTAGTCCCTCATTTATAA |
| 92 | AGGACAAGAAGTGAAGTGCAA--ACAGAACACAAACGTAAATCAATAAAATTCCATTAATCCCCCATTTATAA |
| 93 | ACGACGAGGAGTCGAGTGCAGATGCAGAATGCAGGTGTAAATCAATAAAACTTCATTAGTCCCTCATTTGTGG |
| 94 | AGGACAAGAAGTGAAGTGCAAATGCTGAACGCAAACGTAAATCAATAAAATTCCATTAATCCCCCATTTATAA |
| 95 | AGGACAAGAAGTGAAGTGCAAATACAGAACACAAACGTAAATCAATAAAATTCCATTAATCCCTCATTTATAA |
| 96 | AGGTCGAGGAGTCGAGTGCTA--ACAGAATGCAAACGTAAAT-AATAAAACCTACCTAACCCCCCATATATAA |
| 97 | AGGACGAGGAGTCAGGTGCAAATACAGAATGCAAACGTAAATCAATAAGACTTCATTAGTCCCCCATATATAA |
| 98 | AGGACAAGAAGTGAAGTGCAAATACTGAACACAAACGTAAATCAATAAAATTCCATTAATCCCCCATTTATAA |
| 99 | AGGACAAGAAGTGAAGTCCAAATACAGAACGCAAACGTAAATCAAGAAAATTCCATTAATCCCCCATTTATAA |
| 100 | AGGACAAGAAGTGAAGTGCAAATACTG--CGCAAACGTAAATCAATAAAATTCCATTAATCCCCCATTTATAA |
| 101 | AGGACAAGAAGTGAAGTGCAAATACAAAACACAAACGTAAATCAATAAAATTCCATTAATCCCCCATTTATAA |
| 102 | AGGACGAGAAGTCAAGTGCAAATACAGAACGCAAACGTAAATCAATAAAATTCCATTAATCCCTCATTTATAA |
| 103 | AGGACGAGGAGTCAAGTGCAAATACAGAATGCAAACGTAGATCAATAAAACTTCATTAATCCCTCATTTATAA |
| 104 | GGGACAAGAAGTGAAGTGTAAATACA--ACACAAACGTAAATCAATAAAATTCCATTACTCCCCCATTTATAA |
| 105 | AGGACGAGGAGTCGAGTGCTA--ACAGAATGCAAACGTAAATCAATAAAACTTCATTAATCCCCCATATATAA |
| 106 | AGGACAAGAAGTGAAGTGCAAATACAGAATGCAAACGTAAATCAAGAAAATTCCATTAATCCCCCATTTATAA |
| 107 | AGGACAAGAAGTCAAGTGCAA--ACAGAACGCAAACGTAAATCAATAAAATTCCATCAATCCCTCATTTATAA |
| 108 | AGGACAAGAAGTGAAGCGCAAATACTGTACGCAAGCGTAAATCAATAAAATTCCATTAATCCCCCATTTATAA |
| 109 | AGGACGAGGAGTCGAGTGCTA--ACAGAATGCAAACGTAAATCAATAAAACTT-ATTAACCCCCCATATATAA |
| 110 | AGGACAAAAAGTGAAGTGCAAATACAGAACGCAAACGTAAATCAATAAAATTCCATTAATCCCCCATTTATAA |
| 111 | AGGACAAGAAGCGAAGTGCAAATACAGAACGCAAACGTAAATCAATAAAATTCCATTAATCCCTCGTTTATAA |
| 112 | AGGACAAAAAGTGAAGTGCAAATACAGAATGCAAACGTAAATCGATAAAATTCCATTAATCCCCCATTTATAA |
| 113 | AGGACAAGAAGTGAAGTGCAAATACTGAATGCAAACGTAAATCAATAAAATTCCATTAATCCCCCATTTATAA |
| 114 | AGGACAAAAAGTGAAGTGCAAATACAGAATGCAAACGTAAATCAATAAAATTCCATTAATCCCCCATTTATAA |
| 115 | AGGACGAGGAGTCAGGTGCAAATACAGAATGCAAACGTAAATCAATAAAACTTCATTAATCCCCCATATATAA |
| 116 | AGGACGAGGAGTCGAGTGCTA--ACAGAATGCAAACGTAAATCAACAAAACTTCATT-ACCCCCCATATATAA |
| 117 | AGGACGAGGAGTCAAGTGCAAATACAGAACGCAAACGTAAATCAATAAAACTTCATTAATCCCTCATTTATAA |
| 118 | AGGACGAGAAGTGAAGTGCAAATACAGAACACAAACGTAAATCAATAAAATTCCATTAATCCCCCATTTATAA |
| 119 | AGGACAAGAAGTGAAGTGCAAATACTGAACGCAAACGTAAATCGATAAAATTCCATTAATCCCCCATTTATAA |
| 120 | AGGACAAGAAGTGAAGTGCAAATACTGAACGCAAACGTAAATCAATAAAATTCCATTAATCCCCCATCTATAA |
| 121 | AGAACAGGAAGTCAAGTGCAA-TACAGAACGCAAACGTAAATCAATAAAATTCCATCAATCCCTCATTTATAA |
| 122 | AGGACAAGAAGTGAAGCGCAAATACTGAACGCAAACGTAAATCAATAAAATTCCATTAATCCCCCATTTATAA |
| 123 | AGGACGAGAAGTGAAGTGCAAATACAGAATGCAAACGTAAATCAATAAAATTCCATTAATCCCCCATTTATAA |
| 124 | AGGACGAGGAGTCGAGTGCAA--ACAGAATGCAAACGTAAATCAATAAAACTTCATTAACCCCCCATATATAA |
| 125 | AGGACGAGGAGTCGAGTGCTA--ACAGAATGCAAACGTAAATTAATAAGACCTACCTAACCCCCCATATATAA |
| 126 | AGAACAGGAAGTCAAGTGCAAA-ACAGAACGCAAACGTAAATCAATAAAATTCCATCAATCCCTCATTTATAA |
| 127 | AGGACAAGAAGTGAAGCGCAAATACTGTACGCAAACGTAATTCAATAAAATTCCATTAATCCCCCATTTATAA |
| 128 | AGGACGAGGAGTCGAGTGCTA--ACAGAATGCAAACGTAAA-TAATAAAACCTACCTAACCCCCCATATATAA |
| 129 | AGGGCGAGGAGTCGAGTGCTA--ACAGAATGCAAACGTAAATCAATAAAACCTACCTAACCCCCCATATATAA |
| 130 | AGGACGAGGAGTCGAGTGCTA--ACAGAATGCAAACATAAATCAATAAAACTTCATTAACCCCCCATATATAA |
| 131 | AGGACGAGGAGTCGAGTGCTA--ACACAATGCCAACGTAAATCAATAAAACTTCATTAACCCCCCATATATGA |
| 132 | AGGACGAGGAGTCAAGTGCAAATACAGAATGCAAACGTAAATCAATAAAATTTCATTAATCCCTCATTTATAA |
| 133 | AGGACAAGAAGTGAAGTGTAAATAAAGAACATAAACGTAAATCAATAAAATTCCATTAATGCCCCATTTATAA |
| 134 | AGGACAAGAAGTCAAGTGCAAATACAGAACGCAAACGTAAATCAATAAAATTCCATCAATCCCTCATTTATAA |
| 135 | AGGACAAGAAGTCAAGTGCAAATACAGAACACAAACGTAAATCAATAAAATTCCATTAATCCCTCATTTATAA |
| 136 | AGGACAAGAAGTGAAGTGCTAATACTGAACGCAAACGTAAATCAATAAAATTCCATTAATCCCCCATTTATAA |
| 137 | AGGACAAGAAATGAAGTGCAAATACAGAACGCAAACGTAAATCAATAAAATTCCATTAATGCTCCATTTATAA |
| 138 | AGGACAAGAAGTGAAGTGCAAATACTGAACGAAAACGTAAATCAATAAAATTCCATTAATCCCCCATTTATAA |
| 139 | AGGACGAGGAGTCGGGTGCTA--ACAGAATGCAAACGTAAATCAATAAAACTTCATTAACCCCCCATATATAA |
| 140 | AGGACAAGAAGTGAAGTGCAAATACAG-ACGCAAACGTAAATCAATAAAATTCCATTAATCCCCCATTTATAA |
| 141 | AGGACAAGAAGTGAAGTGCGAATACTGAACGCAAACGTAAATCAATAAAATTCCATTAATCCCCCATTTATAA |
| 142 | AGGACGAGAAGTGAAGCGCAAATACTGTACGCAAACGTAAATCAATAAAATTGCATTAATCCCCCATTTATAA |
| 143 | AGGACAAGAAGTGAAGTGCAA--ACAGAACGCAAACGTAAATCAAGAAAATTCCATTAATCCCCCATTTATAA |
| 144 | AGGACAAGAAGTGAAGTGCAAATAC-GAACGCAAACGTAAATCAATAAAATTCCATTAATCCCCCATTTATAA |
| 145 | AGGACGAGGAGTCGAGTGCTA--ACAGAATGCAAACGTAAATCAATAAAACTTCATTTACCCCCCATATATAA |
| 146 | AGGACGAGGAGTCAAGTGCAA--ACAGAATGCAAACGTAAATCAATAAAACTTCATTAACCCCCCATATATAA |
| 147 | AGGACAAGAAGTGAAGTCCAAATACAGAATGCAAACGTAAATCAATAAAATTCCATTAATCCCCCATTTATAA |
| 148 | GGGACAAGAAGTGAAGTGCAA--ACA--ACACAAACGTAATTCAATAAAATTCCATTACTCCCCCATTTATAA |
| 149 | GGGACAAGAAGTGAAGTGTAA--ACA--ACACAAACGTAAATCAATAAAATTCCATTACTCCCCCATTTATAA |
| 150 | AGGACAAGAAGTGAAGTGCAAATAC-GAACACAAACGTAAATCAATAAAATTCCATTAATCCCCCATTTATAA |
| 151 | AGGACAAGAAGTGAAGTGCAAATACAGAGCACAAACGTAAATCAATAAAATTCCATTAATCCCCCATTTATAA |
| 152 | AGGACGAGGAGTCGAGTGCTA--ACAGAATGCAAACGTAAATCAATAAAACCT-ATTAACCCCCCATATATAA |
| 153 | AGGACGAGGAGTCGACTGCTA--ACAGAATGCAAACGTAAATCAATAAAACTT-ATTAACCCCCCATATATAA |
| 154 | GGGACAAGAAATGAAGTGCAAATACAGAACACAAACGTAAATCAATAAAATTCCATTAATCCCCCATTTATAA |
| 155 | AGGACAAGAAGTGAAGTGCAAATACAGAACGCAAACGTAAATCAATAAAACTCCATTAATCCCTCATTTATAA |
| 156 | AGGACAAGAAGTGAAGTGCAAATACAGAACGCAAACGTAAATCAATAAAATTTCATTAATCCCCCATTTATAA |
| 157 | AGGACAAGAAGTGAAGTGCAAATACAGAACAAAAACGTAAATCAACAAAATTCCATTAATCCCCCATTTATAA |
| 158 | AGGACGAGAAGTCAAGTGCAAATACAGAATGCAAACGTAAATCAATAAAATTCCATTAATCCCTCATTTATAA |
| 159 | AGGACAAGAAGTGAAGTGCAAATACAGAACGCAAACGTGAATCAATAAAATTCCATTAATCCCCCATTTATAA |
| 160 | AGGACAAGAAGTGAGGTGCAAATACAGAACGCAAACGTAAATCAATAAAATTCCATCAATCCCCCATTTATAA |
| 161 | AGGACGAGGTGTCGAGTGCTA--ACAGAATGCAAACGTAAATCAATAAAACTTCATTAACCCCTCATATATAA |
| 162 | AGGATAAGAAGTGAAGTGCAAATACAGAACGCAAACGTGAATCAATAAAATTCCATTAATCCCCCATTTATAA |
| 163 | AGGATAAGAAGTGAAATGCAAATACAGAACGCAAACGTAAATCAATAAAATTCCATTAATCCCCCATTTATAA |
| 164 | AGGACAAGAAGTGAGGTGCAACTACAGAACGCAAACGTAAATCAATAAAATTCCATTAATCCCCCATTTATAA |
| 165 | AGGACAAGAAGTGAAGTGCAAATACAGAGCGCAAACGTAAATCAATAAAATTCCATTAATCCCCCATTTATAA |
| 166 | AGGACAAGAAGTGGAGTGCAAATACAGA-CGCAAACGTAAATCAATAAAATTCCATTAATCCCCCATTTATAA |
| 167 | AGGACAAGAAGTGAAGTGCAAATACAGAATGCAAACGTAAATCAATAAAATTCCATTAATCCCCCATTTGTAA |
| 168 | AGGACAAGAAGTGAAGTGCAAATACAGAACACAAACGTAAATCAATAAAATTCCATTAATCCACCATTTATAA |
| 169 | AGGACAAGAAGTGAAGTGCAAG-ACAGAACGCAAACGTAAATCAATAAAATTCCATTAATCCCCCATTTATAA |
| 170 | AGGACAAGGAGTGAAGTGCAAATACAGAACACAAACGTAAATCAATAAAGTTCCATTAATCCCCCATTTATAA |
| 171 | AGGACAAGAAGTCAAGTGCAAATACAGAATGCAAACGTAAATCAATAAAATTTCATTAATCCCCCATTTATAA |
| 172 | AGGACAAGAAGTGAGGCGCAAATACTGTACGCAAGCGTAAATCAATAAAATTCCATTAATCCCCCATTTATAA |
| 173 | AGGACAAGAAGTCAAGTGCAAATACAGACTGCAAACGTAAATCAATAAAACTTCATTAATCCCTCATTTATAA |
| 174 | AGGACAAGAAGTCAGGTGCAAATACAGAATGCAAACGTAAATCAATAAAACTTCATTAATCCCTCATTTATAA |
| 175 | ACGACGAGGAGTCGAGTGCAAATACAGAATGCAAATGTAAATCGATAAAACTTCATTAGTCCCTCATTTTTAA |
| 176 | ACGACGAGGAGTCGAGTGCAAATGCAGAATGCAAATGTAAATCGATAAAACTTCATTAGTCCCTCATTTATAA |
| 177 | ACGACGAGGAGTCAAGTGCAAATACAGAATGCAAATGTAAATCGATAAAACTTCATTAGTCCCTCATTTATAA |
| 178 | AGGACGAGGAGTCGAGTGCTA--ACAGAATGCAAACGCAAATCAATAAAACTTCATTCACCCCCCATATATAA |
| 179 | AGGACAAGAAGTGGAGTGCAAATACAGAACGCAAACGTAAATCAATAAAATTTCATTAATCCCTCATTTATAA |
| 180 | AGGACAAGAAGTGGAGTGCAAATACAGAATGCAAACGTAAATCAATAAAATTCCATTAATCCCTCATTTATAA |
| 181 | AGGACAAGAAGTGAAGTGCAAATACAGAACGCAAACGTAAATCAATAAAATTCCATTAATCCCCCATTGATAA |
| 182 | AGGACGAGGAGTCGAGTGCTA-GACAGAATGCAAACGTAAATCAATAAAACTTCATTAACCCCCCATATATAA |
| 183 | AGGACAAGAAGTGGAGTGCAA--ACAGAACGCAAACGTAAATCAATAAAATTCCATTAATCCCTCATTTATAA |
| 184 | AGGACGAGGAGTCGAGTGCTA--ACAGAATGCAAACGTAAATTAATAAAACCTACCTAACCCCCTATATATAA |
| 185 | AGGACAAGAAGTGAAGTGCAAATACAGAATGCAAACGTAAATCAATAAAATTCCATCAATCCCCCATTTATAA |
| 186 | AGGACAAGAAGTGAAGTGCAAATACAGAATGCAAACGTAAATCGATAAAATTCCATTAATCCCCCATTTATAA |
| 187 | AGGACAAGAAGTCAAGTGCAA--ACAGAACGCAAACGTAAATCAATAAAATTCCATTAATCCCCCATTTATAA |
| 188 | AGGACAAGAAGTGAAGTGCAAATAAAGAACACAAACGTAAATCAATAAAATTCCATTAATCCCCCATTTATAA |
| 189 | GGGACAAGAAGTGAAGTGCAAATACAGAACGCAAACGTAAATCAATAAAATTCCATTAATCCCCCATTTATAA |
| 190 | AGGACGAGGAGTCGAGTGCTAATACAGAATGCAAATGTAAATCAATAAAACTTCATTAGTCCCTCATTTATAA |
| 191 | AGGACGAGGAGTCGAGTGCTA-GACAGAATGCAAACGTAAATCAATAAAACCTACCTAACCCCCCATATATAA |
| 192 | AGGACGAGGAGTCGAGTGCTAG-ACAGAATGCAAACGTAAATCAATAAAACCTACCTAACCCCCCATATATAA |
| 193 | AGGACGAGGAGTCGAGTGCTA-CACAGAATGCAAACGTAAATCAATAAAACCTACCTAACCCCCCATATATAA |
| 194 | AGGACGAGGAGTCGAGTGCTAC-ACAGAATGCAAACGTAAATCAATAAAACCTACCTAACCCCCCATATATAA |
| 195 | AGGACGAGGAGTCGAGTGCAA-GACAGAATGCAAACGTAAATCAATAAAACTTCATTAATCCCCCATATATAA |
| 196 | AGGACGAGGAGTCGAGTGCAA-CACAGAATGCAAACGTAAATCAATAAAACTTCATTAATCCCCCATATATAA |
| 197 | AGGACGAGGAGTCGAGTGCAAG-ACAGAATGCAAACGTAAATCAATAAAACTTCATTAATCCCCCATATATAA |
| 198 | AGGACAGGAAGTCAAGTGCAA--ACAGAACGCAAACGTAAATCAATAAAATTCCATTAATCCCTCATTTATAA |
| 199 | AGGACAAGGAGTCGAGTGCAAATACAGAATGCAAACGTAAATCAATAAAATTTCATTAATCCCTCATTTATAA |
| 200 | AGGACGAGAAGTCGAGTGCAAATACAGAATGCAAACGTAAATCAATAAAATTTCATTAATCCCTCATTTATAA |
| 201 | AGGACAAGAAGTGAAGTGCAA-CACAGAACGCAAACGTAAATCAATAAAATTCCATTAATCCCCCATTTATAA |
| 202 | AGGACAAGAAGTGAAGTGCAAC-ACAGAACGCAAACGTAAATCAATAAAATTCCATTAATCCCCCATTTATAA |
| 203 | AGGACGAGGAGTCGAGTGCTA-CACAGAATGCAAACGTAAATCAATAAAACTTCATTAACCCCCCATATATAA |
| 204 | AGGACGAGGAGTCGAGTGCTAG-ACAGAATGCAAACGTAAATCAATAAAACTTCATTAACCCCCCATATATAA |
| 205 | AGGACGAGGAGTCAAGTGCAA--ACAGAATGCAAACGTAAATCAATAAAACTTCATT-ATCCCCCATATATAA |
| 206 | AGGACGAGGAGTCAAGTGCAA--ACAGAATGCAAACGTAAATCAATAAAACTTCAT-AATCCCCCATATATAA |
| 207 | AGGACGAGGAGTCGAGTGCAA--ACAGAATGCAAACGTAAATCAATAAAGCTTCATTAACCCCCCATATATAA |
| 208 | AGGACGAGGAGTCGGGTGCAA--ACAGAATGCAAACGTAAATCAATAAAACTTCATTAACCCCCCATATATAA |
| 209 | AGGACAAAAAGTGAAGTGCAAATACAGAACACAAACGTAAATCAATAGAATTCCATTAATCTCCCATTTATAA |
| 210 | AGGACGAGGAGTCGAGTGCTA--ACAGAATGCAAACGTAAAT-AATAAAACCTACCTAACCCTCCATATATAA |
| 211 | GGGACAAGAAGTGAAGTGCAACTACAAAACACAAACGTAAATCAATAAAATTCCATTACTCCCCCATTTATAA |
| 212 | AGGACAAGAAGTGAAGTGCAAATACAAAACACAAACGTAAATCAACAAAATTCCATTAATCCCCCATTTATAA |
| 213 | AGGACAAGGAGTCAAGTGCAAATACAGAATGCAAACGTAAATCAATAAAATTTCATTAATCCCTCATTTATAA |
| 214 | AGGACGAGAAGTCAAGTGCAAATACAGAATGCAAACGTAAATCAATAAAATTTCATTAATCCCTCATTTATAA |
| 215 | AGGACGAGGAGTCGAGTGCTA--ACAGAATGCAAACGTAAAT-AATAAAACCTACCTAACCCCCCACATATAA |
| 216 | AGGACGAGGAGTCGAGTGCTA--ACAGAATGCAGACGTAAATCAATAAAACCTACCTAACCCCCCATATATAA |
| 217 | AGGACGAGAAGTGAAGTGCAAATACAGAACGCAAACGTAAATCAATAAAATTCCATTAATCCCCCATTTGTAA |
| 218 | AGGACGAGAAGTGAAGTCCAAATACAGAACGCAAACGTAAATCAATAAAATTCCATTAATCCCCCATTTATAA |
| 219 | AGGACGAGGAGTCAAGTGCAAATACAGAACGCAAACGTAAATCAATAAAATTCCATTAATCCCTCATTTATAA |
| 220 | AGGACAGGAAGTCAAGTGCAAATACAGAACGCAAACGTAAATCAATAAAATTC-ATCAATCCCTCATTTATAA |
| 221 | AGGACAAGAAGTGAAGTGCAAATACA---CGCAAACGTAAATCAATAAAATTCCATTAATCCCCCATTTATAA |
| 222 | AGGACGAGGAGTCGAGTGCTA--TCAGAATGTAAACGTAAATCAATAAAACTTCATTAACCCCCCATATATAA |
| 223 | AGGACAAGAAGTCGAGTGCAAATACAGAATGCAAACGTAAATCAATAAAACTTCATTAATCCCTCATTTATAA |
| 224 | GGGACAAGAAGTGAAGTGCAAATACAGAACACAAACGTAAATCAATAAAATTCCATTACTCCCCCATTTATAA |
| 225 | AGGACGAGGAGTCGAGTGCAA--ACAGAATGCAAACGTAAATCAATGAAACTTCATTAATCCCCCATATATAA |
| 226 | AGGACAAGGAGTCAAGTGCAAATACAGAACGCAAACGTAAATCAATAAAATTTCATTAATCCCTCATTTATAA |
| 227 | AGGACGAGAAGTCAAGTGCAAATACAGAACGCAAACGTAAATCAATAAAATTTCATTAATCCCTCATTTATAA |
| 228 | AGGTCGAGGAGTCGAGTGCTA--ACAGAATGCAAACGTAAATCAATAAAACCTACCTAACCCTCCATATATAA |
| 229 | AGGACAAGAAGTCGAGTGCAAATACAGAACGCAAACGTAAATCAATAAAACTTCATTAATCCCTCATTTATAA |
| 230 | AGGACAAGAAGTGAAGTGCAAATACTGTACGCAAACGTAAATCAATAAAATTCCATTAATCCCCCATTTATAA |
| 231 | AGGACAAGAAGTGAAGCGCGAATACTGTACGCAAACGTAAAGCAATAAAATTCCATTAATCCCCCATTTATAA |
| 232 | AGGACAAGAAGTGAAGCTCAAATACTGTACGCAAACGTAAAGCAATAAAATTCCATTAATCCCCCATTTATAA |
| 233 | AGGACAAGAAGTCAAGTGCAA--ACAGAACACAAACGTAAATCAATAAAATTCCATTAATCCCTCATTTATAA |
| 234 | AGGACAAGAGGTCAAGTGCAA--ACAGAACGCAAACGTAAATCAATAAAATTCCATTAATCCCTCATTTATAA |
| 235 | AGAACAAGAAGTCAAGTGCAA--ACAGAACGCAAACGTAAATCAATAAAATTCCATTAATCCCTCATTTATAA |
| 236 | AGGTCAAGAAGTGAAGTGCAAATACTGAACGCAAACGTAAATCAATAAAATTCCATTAATCCCCCATTGATAA |
| 237 | AGGACAAGAGGTGAAGTGCAA--ACAGAACGCAAACGTAAATCAATAAAATTCCATTAATCCCTCATTTATAA |
| 238 | AGGACGAGGAGTCCAGTGCTA--ACAGAATGCAAACGTAAATCAATAAAACTTCATTAACCCCCCATATATAA |
| 239 | AGGACAAGAAATGAAGTGCAAATACAGAACACAAACGTAAATCAATAAAATTCCATTAATCCCCCATTTATAA |
| 240 | AGGACGAGAAGTGAAGTCCAAATACAGAATGCAAACGTAAATCAATAAAACTCCATTAATCCCCCAATTATAA |
| 241 | AGGACGAGGAGTCGAGTGCAAATACAGAATGCAAACGTAAATCAATAAAACTTCATTAGTCCCCCATATATAA |
| 242 | ACGACGAGGAGTCGAGTGTAAATACAGAATGCAAATGTAAATCAATAAAACTTCATTAGTCCCTCATTTATTA |
| 243 | ACGACGAGGAGTCGATTGCAAATACAGAATGCAAATGTAAATCAATAAAACTTCATTAGTCCCTCATTTATTA |
| 244 | AGGACAAGAAGTGAAGTGCAAATACAAAACACAAACGTAAATCAATAAAATTCCATTACTCCCCCATTTATAA |
| 245 | AGGACAAGAAATGAAGTGCAAATACAAAACACAAACGTAAATCAATAAAATTCCATTAATCCCCCATTTATAA |
| 246 | AGGACGACGAGTCAAGTGCAAATACAGAATGCAAACGTAAATCAATAAAACTTCATTAATCCCTCATTTACAA |
| 247 | GGGACAAGAAGTGAAGTGCAAACACAAAACACAAACGTAAATCAATAAAATTCCATTACTCCCCCATTTATAA |
| 248 | AGGACGAGGAGTCGAGTGCTA--TCAGAATGCGAACGTAAATCAATAAAACTTCATTAACCCCCCATATATAA |
| 249 | AGGACGAGGAGTCGAGTGCTA--ACTGAATGCAAACGTAAATCAATAAAACCTACTTAACCCCCCATATATAA |
| 250 | AGGACGAGGAGTCAAGTGCAAATACAGAATGCAAACGTAAATCAGTAAAACTTCATTAATCCCTCATTTACAA |
| 251 | AGGACGAGGAGTCAAGTGCAAATACAGAATGCAAACGTAAATCAATAAAACTTCATTAATCTCTCATTTATAA |
| 252 | AGGACGAGGAGTCAAGTGCAA--ACAGAATGCAAACGTAAATCAATAAAACTTCATTAATCCCTCATTTACAA |
| 253 | AGGACGGGGAGTCAAGTGCAA--ACAGAATGCAAACGTAAATCAATAAAACTTCATTAATCCCTCATTTATAA |
| 254 | AGGACAAAAAGTGAAGTGCAAATACAGAACACAAACGTAAATCAATAAAATTCTATTAATCTCCCATTTATAA |
| 255 | AGGACGAGGAGTCGAGTGCTA--ACAGAATGCAAACGTAAAT-AATAAAACCTACCTAACCCCCCCTATATAA |
| 256 | AGGACAAGAAGTCAAGTGCAAATACAGAACACAAACGTAAATCAATAAAACTTCATTAATCCCTCATTTATAA |
| 257 | AGGACAAGAAGTCAAGTGCAAATACAGAACGCAAACGTAAATCAATAAAACTCCATTAATCCCTCATTTATAA |
| 258 | AGGACAAGAAGTCAAGTGCAAATACAGAACGCAAACGTAAATCAATAAAATTCCATTAATCCCCCATTTATAA |
| 259 | AGGACGGGAAGTCAAGTGCAAATACAGAACGCAAACGTAAATCAATAAAATTCCATCAATCCCTCATTTATAA |
| 260 | AGGACAAGAAGTGAAGTGCAAATACAGAACGCAAACGTAAATCAATAAAATTTCATTAATCCCTCATTTATAA |
| 261 | AGGACGAGGAGTCGAGTGCTA--ACAGAATGCAAGCGTAAATTAATAAGACCTATCTAACCCCCCATATATAA |
| 262 | AGGACGAGGAGTCGAGTGCTA--ACAGAATGCAAGCGTAAATTAATAGGACCTACCTAACCCCCCATATATAA |
| 263 | AGGACGAGGAGTCGAGTGCTA--ACAGAATGCAAGCGTAAATTAGTAAGACCTACCTAACCCCCCATATATAA |
| 264 | AGGACGAGGAGTCGAGTGCTA--ACAGAATGCAAGCGTAAATTGATAAGACCTACCTAACCCCCCATATATAA |
| 265 | AGGACGAGGAGTCAAGTGCAA--ACAGAATGCAAACGTAAATCAATAAAACTTCATTAATCCCCCATTTATAA |
| 266 | AGGACGAGGAGTCAAGTGCAAATACAGAATGCAAACGTAAATCAATAAAACTTCATTAGTCCCCCATATATAA |
| 267 | AGGACAAGGAGTCAAGTGCAAATACAGAACGCAAACGTAAATCAATAAAACTTCATTAATCCCTCATTTATAA |
| 268 | AGGACGAGAAGTCAAGTGCAAATACAGAACGCAAACGTAAATCAATAAAACTTCATTAATCCCTCATTTATAA |
| 269 | GGGACAAGAAGTGAAGTGCAAATACAA-ACACAAACGTAAATCAATAAAATTCCATTACTCCCCCATTTATAA |
| 270 | GGGACAAGAAGTGAAGTGCAAATACA-AACACAAACGTAAATCAATAAAATTCCATTACTCCCCCATTTATAA |
| 271 | GGGACAAGAAGTGAAGTGCAAATACA--ACACAAACGTAAATCAATAAAATTCCATTAATCCCCCATTTATAA |
| 272 | GGGACAAGAAGTGAAGTGCAAA-ACA--ACACAAACGTAAATCAATAAAATTCCATTACTCCCCCATTTATAA |
| 273 | GGGACAAGAAGTGAAGTGCAA-TACA--ACACAAACGTAAATCAATAAAATTCCATTACTCCCCCATTTATAA |
| 274 | AGGACAAGAAGTGAAGTGCAAATACTGA-CGCAAACGTGAATCAATAAAATTCCATTAATCCCCCATTTATAA |
| 275 | AGGACAAGAAGTGAAGTGCAA--ACTGAACGCAAACGTAAATCAATAAAATTCCATTAATCCCCCATTGATAA |
| 276 | AGGACGAGGAGTCGAGTGCTA--ACAGAATGCAAACGTAAATCAATAAAACTTCA---ACCCCCCATATATAA |
| 277 | AGGACGAGGAGTTGAGTGCTA--ACAGAATGCAAACGTAAATCAATAAAACTTCAT--ACCCCCCATATATAA |
| 278 | AGGACGAGAAGTCGAGTGCTA--ACAGAATGCAAACGTAAATCAATAAAACTTCAT--ACCCCCCATATATAA |
| 279 | AGGACGAGGAGTCGAGTGCAAATACAGAATGCAAACGTAAATCAATAAGACTTCATTAATCCCTCATTTATAA |
| 280 | AGGACGAGGAGTCGAGTGCAAATACAGAATGCAAACGTAAATCAGTAAAACTTCATTAATCCCTCATTTATAA |
| 281 | AGGACGAGGAGTCGAGTGCAAATACAGAATGCAAACGTAAGTCAATAAAACTTCATTAATCCCTCATTTATAA |
| 282 | AGGACAAAAAGTGAAGTGCAAATATAGAACACAAACGTAAATCAATAAAATTCCATTAATCCCCCATTTATAA |
| 283 | AGGACAAGAAGTGAAGTGCAAATACAG--CACAAACGTAAATCAATAAAATTCCATTAATCCCCCATTTATAA |
| 284 | AGGACAAGAAGTGAAGTGCAAATACAG-GCGCAAACGTAAATCAATAAAATTCCATTAATCCCCCATTTATAA |
| 285 | AGGACGAGAAGTGAAGTGCAAATACAG--CGCAAACGTAAATCAATAAAATTCCATTAATCCCCCATTTATAA |
| 286 | AGGACAAGAAGTGAAGTGCAG--ACAGAACGCAAACGTAAATCAATAAAATTCCATTAATCCCCCATTTATAA |
| 287 | ACGACGAGGAGTCGAGTGCAAATACAGAATGCAAATGTAAATCGATAAAACTTCATTAGTCCCTCATTTATAA |
| 288 | AGGACATGAAGTGAAGTGCAAATACAGAACACAAACGTAAATCAATAAAATTCCCTTAATCCACCATTTATAA |
| 289 | AGGACGTGAAGTGAAGTGCAAATACAGAACACAAACGTAAATCAATAAAATTCCATTAATCCACCATTTATAA |
| 290 | AGGACGAGGAGTCGAGTGCAAATACAGAATGCAAACGTAAATCAATAAAACTTCATTAATCCCTCATATATAA |
| 291 | AGGACGAGGAGTCGAGTGCAAATACAGAATGCAAACGTAAATCAATAAAACTTCATTAATCCCCCATTTATAA |
| 292 | AGGACAAAAAGTGAAGTGCAAATATAGA-CACAAACGTAAATCAATAAAATTCCATTAATCTCCCATTTATAA |
| 293 | AGGACAAAAAGTGAAGTGCAAATACAGAACACAAACGTAAATCAATAAAATTCTATTAATCCCCCATTTATAA |
| 294 | AGGACAAAAAGTGAAGTGCAAATACAGA-CACAAACGTAAATCAATAAAATTCCATTAATCCCCCATTTATAA |
| 295 | AGGACGAGGAGTCGAGTGCTA--ACAGAATGCAAACGTAAATCAATAAAACCTCATTAACCCCCCATATATAA |
| 296 | AGGACGAGGAGTCGGGTGCAA--ACAGAATGCAAACGTAAATCAATAAAACTTCATTAATCCCCCATATATAA |
| 297 | AGGACGAGGAGTCGAGTGCAAATACTGAATGCAAACGTAAATCAATAAAACTTCATTAATCCCCCATATATAA |
| 298 | AGGACGAGGAGTCGAGTGCAAA-ACAGAATGCAAACGTAAATCAATAAAACTTCATTAATCCCCCATATATAA |
| 299 | AGGACGAGGAGTCGAGTGCAA-TACAGAATGCAAACGTAAATCAATAAAACTTCATTAATCCCCCATATATAA |
| 300 | AGGACAAGAAGTGAAGTGCAAATACTG--CACAAACGTAAATCAATAAAATTCCATTAATCCCCCATTTATAA |
| 301 | AGGACAAAAAGTGAAGTGCAAATATAGAACACAAACGTAAATCAGTAAAATTCCATTAATCTCCCATTTATAA |
| 302 | AGGACAAAAAGTGAAGTGCAAATATAGAACACAAACGTAAACCAATAAAATTCCATTAATCTCCCATTTATAA |
| 303 | AGGACAAAAAGTGAAGTGCAAATATAGAGCACAAACGTAAATCAATAAAATTCCATTAATCTCCCATTTATAA |
| 304 | AGGACAAAAAGTGAAGTGCATATACAGAACACAAACGTAAATCAATAAAATTCCATTAATCCCCCATTTATAA |
| 305 | AGGACATGAAGTGAAGTGCAAATACAGAACACAAACGTAAATCAATAAAATTCCATTAATCCCCCATTTATAA |
| 306 | AGGACGAGGAGTCGAGTGCTA--ACAGAATGCAGGCGTAAATTAATAGGACCTACCTAACCCCCCATATATAA |
| 307 | AGGACGAGGAGTCGAGTGCTA--ACAGAATGCAGGCGTAAATTAGTAAGACCTACCTAACCCCCCATATATAA |
| 308 | AGGACGAGGAGTCGAGTGCTA--ACAGAATGCAGGCGTAAATTGATAAGACCTACCTAACCCCCCATATATAA |
| 309 | AGGACGAGGAGTTGAGTGCAAATACAGAATGCAGGCGTAAGTCAGTGAGACTTCATTGGTCCCTCATTTACAG |
| 310 | AGGACGAGGAGTAGAGTGCAAATACAGAATGCAGGCGTAAGTCAGTGAGGCTTCATTGGTCCCTCATTTACAG |
| 311 | AGGACGAGGAGTCGAGTGCTA--ACAGAATGCAAGCGTAAATTAATAAAACCTACCTAACCCCCCATATATAA |
| 312 | AGGACGAGGAGTCGAGTGCTA--ACAGAATGCAGACGTAAATTAATAAAACCTACCTAACCCCCCATATATAA |
| 313 | AGGACGAGGAGTCGAGTGCTA--ACAGAATGCAAACGTAAATCAATAAAACCTCAT--ACCCCCCATATATAA |
| 314 | AGGACGAGGAGTCGAGTGCTA--ACAGAACGCAAACGTAAATCAATAAAACCTACTTAACCCCCCATATATAA |
| 315 | AGGACGAGGAGTCAAGTGCAA--ACAGAATGCAAACGTAAATCAATGAAACTTCATTAATCCCCCATATATAA |
| 316 | ACGACGAGGAGTCGAGTGCAAATGCAGAATGCAAATGTAAATCAATAAAACTTCATTAGTCCCTCATTTATAA |
| 317 | AGGACGAGGAGTCGAGTGCAAATACAGA-TGCAAACGTAAATCAATAAAACTTCATTAATCCCCCATATATAA |
| 318 | AGGACAAGAAGCGAAGTGCAAATACAGAACGCAAACGTAAATCAATAAAGTTCCATTAATCCCTCGTTTATAA |
| 319 | AGGACAAGAAGCGAAGTGCAAATACAGAACGCAAACGTAAATCAGTAAAATTCCATTAATCCCTCGTTTATAA |
| 320 | AGGACAAGAAGCGAAGTGCAAATACAGAGCGCAAACGTAAATCAATAAAATTCCATTAATCCCTCGTTTATAA |
| 321 | AGAACAGGAAGTCAAGTGCAAATACAGAACGCAAGCGTAAATCAATAAAATTCCATCAATCCCTCATTTATAA |
| 322 | AGGACGAGGAGTCAGGTGCAAATACAGAATGCAAACGTAAATCAATAAGACTTCATTAATCCCCCATATATAA |
| 323 | AGGACGAGGAGTCAAGTGCAAATACAGAACGCAAACGTAAATCAATAAAACTTCATTAATCCCCCATATATAA |
| 324 | AGGACGAGGAGTCAAGTGCAAATACTGAATGCAAACGTAAATCAATAAAACTTCATTAATCCCCCATATATAA |
| 325 | AGGACGAGGAGTCGAGTGCAAATACAGAATGCAAACGTAAATCAATAAAACTTCATTAATCCCTCATTTACAA |
| 326 | AGGACGAGGAGTCGAGTGCAAATACAGAATGCAAACGTAAATCAATAAAACTTCATTAGTCCCTCATTTATAA |
| 327 | AGGACGAGGAGTCGAGTGCAAATACAGAATGCAAACGTAAATCAATAAAACTTCATTGATCCCTCATTTATAA |
| 328 | AGGACGAGGAGTCGAGTGCAAATACAGAATGCAAATGTAAATCAATAAAACTTCATTAATCCCTCATTTATAA |
| 329 | AGGACGAGGAGTTGAGTGCAAATACAGAATGCAAACGTAAATCAATAAAACTTCATTAATCCCTCATTTATAA |
| 330 | ACGACGAGGAGTCGAGTGCAAATACAGAATGCAAACGTAAATCAATAAAACTTCATTAATCCCTCATTTATAA |
| 331 | AGGACGAGAAGTGAAGTCCAAATACAGA-TGCAAACGTAAATCAATAAAATTCCATTAATCCCCCATTTATAA |
| 332 | AGGACAAGAAGTGAAGTGCAA-TACTGAACGCAAACGTGAATCAATAAAATTCCATTAATCCCCCATTGATAA |
| 333 | AGGTCAAGAAGTGAAGTGCAAATACTGAACGCAAACGTGAATCAATAAAATTCCATTAATCCCCCATTGATAA |
| 334 | AGGACGAGGAGTCAAGTGCAAATACAGAATGCAAACGTAAATCAATAAAACTTCATTAATCCCCCATTTATAA |
| 335 | AGGACGACGAGTCAAGTGCAAATACAGAATGCAAACGTAAATCAATAAAACTTCATTAATCCCTCATTTATAA |
| 336 | GGGACAAGAAGTGAAGTGTAAATACA--ACACAAACGTAAATCAATAAAATTCCATTAATCCCCCATTTATAA |
| 337 | GGGACAAGAAGTGAAGTGTAAA-ACA--ACACAAACGTAAATCAATAAAATTCCATTACTCCCCCATTTATAA |
| 338 | GGGACAAGAAGTGAAGTGTAA-TACA--ACACAAACGTAAATCAATAAAATTCCATTACTCCCCCATTTATAA |
| 339 | AGGACAAGGAGTCAAGTGCAAATACAGAATGCAAACGTAAATCAATAAAACTTCATTAATCCCTCATTTATAA |
| 340 | AGGACGAGAAGTCAAGTGCAAATACAGAATGCAAACGTAAATCAATAAAACTTCATTAATCCCTCATTTATAA |
| 341 | AGGACAAGAAGTCGAGTGCAAATACAGAACGCAAACGTAAATCAATAAAATTTCATTAATCCCTCATTTATAA |
| 342 | AGGACAAAAAGTGAAGTGCAAATATAGAACACAAACGTAAATCAATAAAATTCCATTAATCTTCCATTTATAA |
| 343 | AGGACAAAAAGTGAAGTGCAAATATAGAACACAAACGTAAATCAATAAAATTCTATTAATCTCCCATTTATAA |
| 344 | GGGACAAGAAGTGAAGTGTAAATACAA-ACACAAACGTAAATCAATAAAATTCCATTACTCCCCCATTTATAA |
| 345 | GGGACAAGAAGTGAAGTGTAAATACA-AACACAAACGTAAATCAATAAAATTCCATTACTCCCCCATTTATAA |
| 346 | AGGACAAGAAGTCAAGTGCAAATACAGAATGCAAACGTAAATCAATAAAATTCCATTAATCCCTCATTTATAA |
| 347 | AGGACAGGAAGTCAAGTGCAAATACAGAACGCAAACGTAAATCAATAAAATTCCATTAATCCCTCATTTATAA |
| 348 | AGGACAAGAAGTGAGGTGCAACTACAGAACACAAACGTAAATCAATAAAATTCCATTAATCCCCCATTTATAA |
| 349 | AGGACAAGAAGTGAAGTGCAAATACTGA-TGCAAACGTAAATCAATAAAATTCCATTAATCCCCCATTTATAA |
| 350 | GGGACAAGAAATGAAGTGCAAATACAAAACACAAACGTAAATCAATAAAATTCCATTACTCCCCCATTTATAA |
| 351 | AGGACAAGAAGTGAAGTGCAAATACAGAACACAAACGTAAATCAATAAAATTCCATTAATCTCCCATTTATAA |
| 352 | AGGACAAGAAGTGAAGTGCAAATACAGAACACAAACGTAAATCAATAAAATTCCATTACTCCCCCATTTATAA |
| 353 | AGGACAAGAAGTGAAGTGCAAATACAGA-CACAAACGTAAATCAATAAAATTCCATTAATCCCCCATTTATAA |
| 354 | GGGACAAGAAGTGAAGTGCAAATACAGAACACAAACGTAAATCAATAAAATTCCATTAATCCCCCATTTATAA |
| 355 | AGGACAAGAAGTGAAGTGCAA-TACAGAACGCAAACGTAAATCAATAAAATTCCATTAATCCCCCATTTATAA |
| 356 | AGGACAAGAAGTGAAGTGCAAA-ACAGAACGCAAACGTAAATCAATAAAATTCCATTAATCCCCCATTTATAA |
| 357 | AGGACAAGAGGTGAAGTGCAA--ACAGAACGCAAACGTAAATCAATAAAATTCCATTAATCCCCCATTTATAA |
| 358 | AGGCCAAGAAGTCAAGTGCAAATACAGAACGCAAACGTAAATCAATAAAATTCCATTAATCCCTCATTTATAA |
| 359 | AGAACGAGGAGTCGAGTGCTA--ACAGAATGCAAACGTAAATCAATAAAACCTACCTAACCCCCCATATATAA |
| 360 | AGGACGAGGAGTCAAGTGCAAATACAGAACGCAAACGTAAATCAATAAA-TTTCATTAATCCCTCATTTATAA |
| 361 | AGGACGAGGAGTCGAGTGGTA--ACAGAATGCAAACGTAAATCAATAAAACCTACCTAATCCCCTATATATAA |
| 362 | AGGACGAGAAGTGAAGTCCAAATACTGAATGCAAACGTAAAACAATAAAGTTCCATTAATCCCCCATTTATAA |
| 363 | AGGACGAGGAGTCGAGTGCTA--ACAGAATGCAAACGTAAATCAATAAAACCTACCTAACCCTCCATATATAA |
| 364 | AGGACAAGAAGTGAAGTGCAAATACTGAACCCAAACGTAAATCAATAAAATTCTATTAATCCCCCATTTAAAA |
| 365 | AGGACAAGAAGTGAAGTGCAAATACAGAGCACAAACGTAAATCAATAAAATTCCATTAATCCTTCATTTATAA |
| 366 | AGGACAAAAAGTGAAGTGCAAATACAGAACACAAACGTAAATCAATAGAATTCTATTAATCTCTCATTTATAA |
| 367 | AGGACAAGAAGTGAAGTGCAA--ACTGA-CGCAAACGTAAATCAATAAAATTCCATTAATCCACCATTTATAA |
| 368 | AGGATGAGGAGTCAAGTGCTA--ACAGAATGCAAACGTAAATCAATAAAACTTCATTAACCCCCCATATATAA |
| 369 | AGGTCAGGAAGTGAAGTGCAAATATTGA-CGCAAACGTGAATCAATAAAATTCCATTTATCCCCCATTGATAA |
| 370 | AGGACGAAGAGTCGAGTGCTA--ACAGAATGCAAACGTAAATCAATAAAACCTACCTAACCCCCCATATATAA |
| 371 | AGGACGAGGAGTCGAGTGCTA--ACAGAATGCAAACGTAAATCAATAAAACCTACCTAACCCC-CATATATAA |
| 372 | ACGACGAGTAGTCGAGTGCAGATGCAGAATGCGGGTGTAAATCAATAAAACTTCATTAGTCCCTCATTTGTGG |
| 373 | AGGACAAGAAGTGAAGTGCAAATACAGAACGCAAATGTAAATCAATAAAATTCCATTAATCCCCCATTTATAA |
| 374 | AGGACAAGAGGTGAAGTGCAA-TACAGAATGCAAACGTAAATCAAGAAAATTTCATTAATCCCTCATTTATAA |
| 375 | AGGACGAGGAGTCGAGGGCTA--ACAGAATGCAAACGTAAATCAATAAAACCTACCTA-CCCTCCATATATAA |
| 376 | AGGACAAGAAGTGAAGCGCAAATACTGTACGCAAACGTAAATCAATACAATTCCATTAATCCCCCATTTATAA |
| 377 | AGGACGAGAGGTGAAGTCCAAATACAGAATGCAAACGTAAATCAATAAAATTCCATTAATCCCCCATTTATAA |
| 378 | AGGACAAAAA-TGAAGTGCAAAT--AGAACACAAACGTAAATCAATAAAATTCCATTAATCTCCCATTTACAA |
| 379 | AGGACAAGAAGG-GAGTGCAAATACAGAACGCAAACGTAAATCAATAAAATTCCATTAATCCCCCATTTATAA |
| 380 | AGGACGAGGAGTCGAGTGCAA--ACAGAATGCAAACGTAAATCAATAA-ACTTCATTAATCCCCCATATATAA |
| 381 | AGGACGAGGAGTCGAGTGCTA--ACAGAATGCAAACGTAGATCAATAAAACCTACCTAACCCCCCAGATATAA |
| 382 | AGGACAGGAAGTCAAGTGCAAATACAGAACGCAAACGTAAATCAATAAAATTCCATCAATCCCTCATCTATAA |
| 383 | GGGACAAGAAATGAAGTGCAAATACAAAACACAAACGTAAATCAATAAAGTTCCATTAATCCCCCATTTATAA |
| 384 | AGGACAAGAAGTGAAGTGCAAATACAGAACGCAAACGTAAATCAATAAAATTCCATTAATCCCCCATTCATAA |
| 385 | AGGACGAGGAGTCGAGTGCTA--ACAGAATGCAAACGTAAATCAATAAA-CTTCATT-ACCCCCCATATATAA |
| 386 | AGGACGAGAAGTCAAGTGCAAATAC---GTGCAAACGTAAATCAATAAAACTTCATTAATCCCTCATTTATAA |
| 387 | AGGACGAGGAGTCAAGTGCAA--ACAGAATGCAAACGTAAATCAA-GAAACTTCATTA-TCCCCCATATATAA |
| 388 | GGGACAAGAAGTGAAGTGCAAATACA--ACACAAACGTAAA-CAATAAAATTCCATTAATCCCCCATTTATAA |
| 389 | AGGACAAGAAGTGAAGTGCAAATGCTGAACGCAAACGTAAATCAATAAAATTCCATTA-TTCCCCATTTATAA |
| 390 | AGGACAAGAAGTGAAGTGCAAATACAGAACGCAAACGTAAATCAATAAAATTCCATTAATCTCCCATTTATAA |
| 391 | AGGACGCGGAGTCGAGTGCAA--ACAGAATGCAAACGTAAATCAATAAAACCTCATTAATCCCCCATATATAA |
| 392 | AGGACGAGGGGTCAAGTGCAAATACAGAATGTAAACGTAAAGCAATAAAACTTCATTAATCCCCCATATATAA |
| 393 | AGGACGAGGAGTCCAGTGCTA--ACAGAATGCAAACGTAAGTCAATAAAACTTCATTAACCCCCCATATATAA |
| 394 | AGGACAAGAAGTGAAGTGCAAAT-CTGAACGCAAACGTAAATCAATAAAATTCCATTAATCCCCCATTGATAA |
| 395 | AGGACAAGAAGTGAAGCGCAAATACTGTACACAAACGTAAATCAATAAAATTCCATTAGCCCCCCATTTATAA |
| 396 | AGGACAAAAAGTGAAGTGCAAATACAGA-CACAAACGTAAATCAATAAAATTCTATTAATCTCCCATTTATAA |
| 397 | AGGACGAGGAGTCGAGTGCTA--ACAGAATGCAAACGTAAATCAATAAAACCCACCTAACCCCCCATATATAA |
| 398 | AGGACGAGAAGTGAAGTGCAAATACAG--TGCAAACGTAAATCAATAAAATTCCATTAATCCCCCATTTATAA |
| 399 | AGGACAAGAGGTGAAGCGCAAATACTGTACGCAAACGTAATTCAATAAAATTCTATTAATCCCCCATTTATAA |
| 400 | AGTACATGAAGTGAAGTGCAAATACAGAACACAAACGTAAATCAATAAAATTCCATTAATCCACCATTTATAA |
| 401 | AGGACGAGGAGTCAAGTGCAAATACAGAACGCAAACGTAAATCAATAAAGCTTCATTAATCCCCCATATATAA |
| 402 | AGGATAAGAAGTGAAGTGCAAATACAGAACGCAAACGTAAATCAATAAAATTTCATTAATCCCCCATTTATAA |
| 403 | AGGACCAGAAGTGAAGCGCAAATACTGTACGCAAACGTAAATCAATAAAACTCCATGAATCCCCCATTTATAA |
| 404 | AGGACAAGAAGTGAAGTGCAAATACTGAACGCAAACGTAAATCAAGAAAATTCCATTAATCCCCCATTTATAA |
| 405 | AGGACGAGGAGTCAGGTGCTA--ACAGAATGCAAACGTAAATCAATAAAACTTCATTAACCCCCCATATATAA |
| 406 | ACGACGAGGAGTCGAGTGCAAATACAGAATGCAAATGTAAATCACTAAAACTTCATTAGTCCCTCATTTATAA |
| 407 | AGGACGAGGAGTCGAGTGCTA--ACAGAATGCAAACGTAAATCAATAAAACTTCATTAACCCCCCATGTATAA |
| 408 | AGGACGAGGATTCGAGTGCAA--ACAGAATGCAAACGTAAATCAATAAAACTTCATTAATCCCCCATATATAA |
| 409 | AGGACAAGAAGTCAAGTGCAAATACAGAACGCAAACGTAAATCAATAAAACTTCATTAATTCCTGATTTATAA |
| 410 | AGGACGAGCAGTCAAGTGCAAATACAGAATGCAAACGTAGATCAATAAAACTTCATTAATCCCTCATTTATAA |
| 411 | AGGACAAGGAGTCGAGTGCTA--ACAGAATGCAAACGTAAAT-AATAAAACCTACCTAACCCCCTCCATATAA |
| 412 | AGGACGAGGAGTCAAGTGCAAATACAGAATGCAAACGTAAATCAATAAAACTTCATTAATCCCTCATATATAA |
| 413 | GGGACAAGAAGTGAGGTGTAAATACAAAACACAAACGTAAATCAATAAAATTCCATTACTCCCCCATTTATAA |
| 414 | AAGACAAGAAGTGAAGTGCGAATACAG--CACAAACGTAAATCAATAAAATTCCATTAATCCCCCATTTATAA |
| 415 | AGAACAAGAAGTGAGGT-CAGCTACAGAACACAAACGTAAATCAATAAAATTCCATTAATCCCCCATTTATAA |
| 416 | AGGACAAGAAGTGAAGCG-AAATACTGAACGCAAACGTAAATCAATAAAATCCCAT--ATCCCCCATCTATAA |
| 417 | AGGACGAGGAGTCAAGTGCAAATACAGAATGCAAACGTAAATCAATAAAACTTCATTAACCCCCCATATATAA |
| 418 | AGGACGAGGAGTCGAATGCTA--ACAGAATGCAAACGTAAATCAATAAAACTTCATTAACCCCCCATATATAA |
| 419 | AGGACAAGAAGTGAAGTGCAAATACAGAATGCAAACGTAAATCAATAGAATTCCATTAATCCCACGTTTATAA |
| 420 | AGGACATGAAGTGAAGTGCAAATACAGA-CACAAACGTAAATCAATAAAATTCCATTAATCCACCATTTATAA |
| 421 | AGGACGAGGAGTCAAGTGCTA--ACAGAATGCAAACGTAAGTCAATAAAACCTACCTAACCCCCCATATATAA |
| 422 | AGGACGAGGAGTCGAGTGCTA--ACAGAATGCAAACGTAAATCAATAAAACTTACCTAACCCCCCATATATAA |
| 423 | AGGACAAGAGGTGGAGTGCAA--ACAGAACGCAAACGTAAATCAATAAAATTCCATTAATCCCTCATTTATAA |
| 424 | ACGACGAGGAGTCGAGTGCAGATGCAGAATGCGGGTGTAAATCGATAAAACTTCATTAGTCCCTCATTTGTGG |
| 425 | AGGACAAGAAGTCAAGGGCAAATATAGAACACAAACGTAAATCAATAAAATTCTATTAATCTCCCATTTATAA |
| 426 | AGGACAAGAAGTCAAGTCCAAATACAGAATGCAAACGTAAATCAATAAAATTTCATTAATCCCTCATTTATAA |
| 427 | AGGACGAGGAGTCAAGTGCTA--ACAGAATGCAAACGTAAATCAATAAAACCTACTTAACCCCCCATATATAA |
| 428 | AGGACAAGAAGTGAAGTGCAAATACAGAACGCAAACGTAA-TCAATAAAATTCCGTTAATCCCCCATTTATAA |
| 429 | AGGACAAGGAGTCGAGTGCTA--ACAGAATGCAAACGTAAATCAATAAAACTTCATTAACCCCCCATATATAA |
| 430 | AGGACGAGGAGTCAAGTGCAA--ACAGAATGCAAGCGTAAATTAATGAAACTTCATTAATCCCCCATATATAA |
| 431 | AGGACAAGGAGTGAAGTGCAAATACAGAACACAAACGTAAATCAATAAAATTCCATTAATCCCCCATTTATAA |
| 432 | AGGACGAGGAGTCAAGTGCAA--ACAGTATGCAAACGTAAATCAATAA-ACTTCATTAATCCCCCATATATAA |
| 433 | AGGACAAGGAGTGAAGTGCGAATACAGAACGCAAACGTAAATCAAGAAAATTCCATTAATCCTCCATTTATAA |
| 434 | AGGACAAAAAGTGAAGTGCAAATACAGAACGCAAACGTAAATCAAGAAAATTCCATTAATCCCCCATTTATAA |
| 435 | AGGACAAGAAGTGAAGCGCAAATACTGTACGCAAACGTAAATCAATAAAATTCCATTAATCCCCCATTTATA- |
| 436 | AGGACGAGGAGTC-AGTGCAA--ACAGAATGCA-ACGTAAATCAATGAAACTTCA---ATCCCCCATATATAA |
| 437 | GGGACAAGAAGTGAAGTGCAAATACAGAATGCAAACGTAAATCAATAA-ATTCCATCAATCCCCCATTTATAA |
| 438 | AGGACGAGGAGTCGAGTGCAAATACAGAACGCAAACGTAAATCAATAAAACTTCATTAATCCCCCATATATAA |
| 439 | AGGACGAGGAGT-CAGTGCTA--ACAGAATGCAAACGTAAATCAATAAAACTTCATTAACCCCCCATATATAA |
| 440 | AGGACAAAAAGTGAAGTGCAA--ACAGAACACAAACGTAAATCAAGAAAATTCCA---ATCCCCCATTTATAA |
| 441 | AGGACGAGGAGTCAAGTGCAAATACAGAATGCAAACGTAAATCAATAAAACTTCATTAATCC--CATATATAA |
| 442 | AGGACAAGAAGTGAAGTGCAA--ACTGAACGCAAACGTAAATCAATAAAATTCCATTAATCCCCCATCTATAA |
| 443 | AGAACAGGAAGTCAAGTGCAAATACAGAAAGCAAGCGTAAATCAATAAAATTCCATCAATCCCTCATTTATAA |
| 444 | AGGACAAAAAGTGAGGTGCAAATACAGAATGCAAACGTAAATCAATAAAATTCCATCAACCCCCCATTTATAA |
| 445 | AGGACAAGAAGTGAGGTGCAAATGCTGAACGCAAACGTAAATCAATAAAATTCCATTAATCCCCCATTTATAA |
| 446 | AGGACGAGGAGTCGAGTGCTA--ACAGAATGCAAACGTACATCAATAAAACCTACCTAACCCCCCATATATAA |
| 447 | ACGACGAGTAGTCGAGTGCAAATACAGAATGCAAATGTAAATCAATA-AACTTCATTAATCCCTCATTTATAA |
| 448 | AGGACAAGAAGTGAGGCGCAAATACTGTATGCAAACGTAAATCAATAAAATTCTATTAATCCCCCATTTATAA |
| 449 | AGGACGAGGAGTCGAGTGCTA--ACAGAATGCAAACGTAAATCAATAAAACTTCATTAATCCCTCATATATAA |
| 450 | AGGACGAGGAGTCAAGTGCAA--ACAGAATGCAAACGTAAATCAATAAAACTTCATTAATCCC-CATATATAA |
| 451 | GGGACAAGAAGTGAAGTGCAAATACAGAACGCAAGCGTAAGTCAATAAAATTCCATTAGTCCCCCGTTTATAA |
| 452 | AGGACAAGGAGTCGAGTGCAAATACAGAATGCAAACGTAAATCAATAAAACTTCATTAATCCCCCATATATAA |
| 453 | AGGACAAGAAGCGAAGTGCAAATACAGAGCGCAAACGTAAATCAGTAAAGTTCCATTAATCCCTCGTTTATAA |
| 454 | GGGACAAGAAGTGAAGTGCAA--ACA--ACACAAACGTAAAGCAATAAAATTCCATTACTCCCCCATTTATAA |
| 455 | AGGACGAGGAGCCGAGTGCTA--ACAGAATGCAAACGTAAATCAATAAAACTTCATTAACCCCCCATATATAA |
| 456 | AGGATAAGAAGTCGAGTGCAAATACAGAATGCAAACGTAAATCAATAAAATTTCATTAATCCCTCATTTATAA |
| 457 | AGGACGAGAAGTGAAGCGCAAATACTGTACGCAAACGTAATTCAATAAAATTCCATTAATCCCACATTTATAA |
| 458 | AGGACAAGAAGTGAAGCGCGAATACTGTACGCAAGCGTAAATCAATAAAATTCCATTAATCCCCCATTTATAA |
| 459 | AGGACAAGAAGTGAAGCGCAAATACTGTACACAAACGTAAATCAATAAAATTCCATTAATCCCCCATTTATAA |
| 460 | ACGACGAGGAGTCAAGTGCAAATACAGAATGCAAATGTAAATCAATAAAACTTCATTAGTCCCTCATTTATAA |
| 461 | AGGACGAGGAGTCAAGTGCAA--ACAGAATGCAAACGTAAATCAATAAAACTTCATTAATCCCCCGTATATAA |
| 462 | AGGACAAGAAGTCAAGTGCATATAC-GA-TGCAAACGTAAATCAATAAAATTTCATTAATCCCCCATTTATAA |
| 463 | AGGACGAGGAGTCGAGTGCTA--ACAGAATGCAAACGTAAATAAATAAAACTTCATTAACCCCCCATATATAA |
| 464 | GGGACAAGAAGTGAAGTGTAAATACAAAACACAAACGTAAATCAATAAAATTCCATTGCTCCCCCATTTGTAA |
| 465 | GGGACAAGAAGTGAGGCGCAAATACTGTACGCAGGCGTAAATCGATAAAATTCCATTAGTCCCCCATTTATAA |
| 466 | ACGACGAGGAGTCGAGTGCAAATACAGAATGCAAATGTAAATCAATAAAACTTCATTA-TCCCTCATTTATTA |
| 467 | AGGACAAAAAGTGAAGTGCAAATATAGAACAAAAACGTAAATCAATAAAATTCCATTAATCTCCCATTTATAA |
| 468 | AGGACGAGGAGTCAAGTGCAAATACAGAATGCAAACGTAAATCAATAAGACTTCATTAATCCCTCATTTATAA |
| 469 | AGGTCGAGGAGTCGAGTGCTA--ACAGAATGCAAACGTAAATCAATAAAACCTATCTAACCCCCCATATATAA |
| 470 | AGGACAAGAAGTCAAGTGCAAATACAGAACACAAACGTAAATCAATAAAATTTCATTAATCCCTCATTTATAA |
| 471 | AGGACAAGAAGTGGAGCGCAAATACTGTACGCAAACGTAAATCAATAAAA-TCCATTCATCCCCCATTTATAA |
| 472 | AGGACAAAAAGTGAAGTGCAAATACAGAACACAAACGTAAA-CAATAAAATTCCATTAATCTTCCATTTATAA |
| 473 | AGGATAAGAAGTGAAGTGCAAATAC---GCGCAAACGTAAATCAATAAAATTCCATTAATCCCCGATTTATAA |
| 474 | AGGACAAGAAGTGAAGTGCAAATACTGAACGCAAACGTGAATCAATAAAATTCCATTAATTCCCCATTGATAA |
| 475 | AGGACGAGGAGTCGAGTGCTA--ACAGAATGCAAACGTAAATCAGTAAAACTTCATTAACCCCCCATATATAA |
| 476 | AGGACGAGGAGTCGAGTGCTA--ACAGAATGCAAACGTAA-TCAATAAAACTTCATTAACCCCCCGTATATAA |
| 477 | AGGACGAGGAGTCGAGTGCAA--AAAGAGTGCAAACGTAAATCAATAAAACTTCATTAATCCCCCATATATAA |
| 478 | AGGACAGGAAGTCAAGTGCAAATACAGAAAGCAAACATAAATCAATAAAATTCCATCAATCCCTCATTTATAA |
| 479 | AGGACAAGAAGTGAAGTGCAAATACAGAACGCAAGCGTAAATCAAGAAAATTCCATTAATCCCCCATTTATAA |
| 480 | AGGACAAGAAGTGAAGTGCAA--ACTGAACGCAAACGTGAATCAATAAAATTCCATTAATCCCCCATTGATAA |
| 481 | AGGACAAGAAGTGAAGTGCAAATACTGAACGCAAACGTAAATCAGTAAA-TTCCATTAATCCCCCATTTATAA |
| 482 | AGGACGAGGAGTCGAGTGCTA--ACAGAATGCAAACGTAAATCGATAAAACCTACCTAACCCCCCATATATAA |
| 483 | AGGTCAAGAAGTGAAGTGCAA-TACTGAACGCAAACGTAAATCAATAAAGTTACATTAATCACCCATTGATAA |
| 484 | AGGACGAGAAGTGAAGTCCAAATACAGAATGCAAACGTGAATCAATAAAATTCCATTAATCCCCCATTTATAA |
| 485 | AGGACAAGAAGTGAAGCGCAAATACTGAACGCAAACGTGAATCAATAAAATTCCATTAATCCCCCATTTATAA |
| 486 | AGGACGAGAAGTGAGGTCCAAATACAGAATGCAAACGTAAATCAATAAAATTCCATTAATCCCCCATTTATAA |
| 487 | AGGACGAGGAGTCGAGTGCTA--ACAGAATGCAAACGCAAATCGATAAAACTTCATTAACCCCCCATATATAA |
| 488 | AGGACAGGAAGTCAAGTGCAAATACAGAAAGCAAACGCAAATCAATAAAATTC-ATCAATCCCTCATTTATAA |
| 489 | AGGACAAGAAATGGA-TGCAAATACTGA-CGCAAACGTGAATCAATAAAATTCCATTAATCCCCCATTTATAA |
| 490 | AGGACAAGAAGTGAAGTGCAAATACGG--CACAAACGTAAATCAATAAAATTCCATTAATCCCCCATTTATAA |
| 491 | AGGACAAGAAGTGAAGTACAAATACAGAACGCAAACGTAAATCAATAAAATTCCATTAATCCCCCATTTATAA |
| 492 | AGGACAAGAAGTGAAGTGCAAATACAGAACACAAACGTAAA-CAATAAAATTCCATTAATCCCCCATTTATAA |
| 493 | AGGACAGAAAGTGAAGTGCAAATACAGAACACAAACGTAAATCAATAAAATTCCATTAATCCCCCATTTATAA |
| 494 | AGGACGAGGAGTCGAGTGCTA--ACAG--TGCAAACGCAAATCAATAAAACTTCATTAACCCCCCATATATAA |
| 495 | AGGACGAGGAGTCGAGTGCTA--ACAGAATGCAAACGTAAATCAATAAATCTTCATTAACCCCCCATATATAA |
| 496 | AGGACAAGAGGTGAAGTGCAA--ACAGAACGCAAACGTAAATCGATAAAATTCGATTAATCCCCCATTTATGA |
| 497 | ACGACGAGGAGTCGAGTGCAGATGCAGAATGCGAGTGTAAATCAATAAAACTTCATTAGTCCCTCATTTGTGG |
| 498 | AGGACGAGGAGTCGAGTGCAA--ACAGAATGCAAACGTAAATCAATAAAACTTCACTAATCCCCCATATATAA |
| 499 | AGGACGAGGAGTCAAGTGCAAATACAGAATGCAAATGTAGATCAACAAAACTTCATTAATCCTTCATTTATAA |
| 500 | GGGACAAGAAGTGAAGTGTAAATACAAAACACAAACGTAAATCAATAAAATTCCATTACTCCCCCATTTGTAA |
| 501 | AGGACGAGGGGTCAAGTGCAAATACAGAATGCAAACGTAATTCAATAAAACTTCATTAATCCCTCATTTATAA |
| 502 | AGGACGAGGAGTTGAGTGCTA--ACAGAATGCAAACGTAAATCAATAAAACTTCATTAACCCTCCATATATAA |
| 503 | AGGACAAGAAGTGAAGTGCAAATAC---ACACAAACGTAAATCAATAAAATTCCATTAATCCCCCATTTATAA |
| 504 | AGGACAAGAAGTGAAGTGCAAATACTGAACGCAAACGTAGATCAATAAGATTCCATTGATCCCCCATTTATAA |
| 505 | AGGACGAGGAGTCGAGTGCTA--ACAGAATGCAAACGTAGATCAATAAAACTTCATTAACCCCCCATATATAA |
| 506 | AGGACGAGGAGTCAAGTGCAAATACAGA-TGCAAACGTAAATCAATAAAACTTCATTAATCCCCCATATATAA |
| 507 | AGGACGAGGAGTCGAGTGCTA--ACAGAATGCAAACGTAAATCAATAAAACCT---TAACCCCCCATATATAA |
| 508 | AGGACAAGAAGTGAAGTGCAAATACAAAACAAAAACGTAAATCAACAAAATTCCATTAATCCCCCATTTATAA |
| 509 | AGGACGTGAAGTGAAGTGCAAATACAGAACACAAACGTAAATCAATAAAATTCACTTAATCCACCATTTATAA |
| 510 | AGGACAAAAAGTGAAGTGCAAATACAGAACACAAACGTAAATCAATAAAATTCCGTTAATCCCCCATTTATAA |
| 511 | AGGACAAGAAGTGAAGCGCAAATACTGTACGCAAACGTAAATCAATAAAATCCCATTAATCCCCCATTTATAA |
| 512 | AGGACAAGAAGTGAAGTGCAAATACTGAACGCAAACGTAAATCAATAAAATTCCATTGATCCCCCATTTATAA |
| 513 | AGGACAAAAAGTGAGGTGCAAATACAGAACACAAACGTAAATCAATAAAATTCCATTAAGCCACCATTTATAA |
| 514 | ACGACGAGGAGTCGAGTGCAGATGCAGAATGCAGGTGTAAATCAATAAAACTTCATTAGTCCCTGATTTGTGG |
| 515 | AGGACAAGAAGTGAAGTGCAAACACAGAACGCAAACGTAAATCAATAAAATTCCATTAATCCCCCGTTTATAA |
| 516 | AGGACAAGAAGTGAAGTGCAAATACAGAACGCAAACGTAAATCAATAAAATTCTATTAATCCCCCATTTATAA |
| 517 | AGGACAAGAAGCGAAGCGCAAATACTGTACGCAAGCGTAAATCAATAAAATTCCATTAATCCCCCATTTATAA |
| 518 | AGGACGAGGAGTCGACTGCTA--ACAGAATGCAAACGTAAATCAATAAAACCT-ATTAACCCCCCATATATAA |
| 519 | AGAAGAGGAAGTCAAGTGCAAATACAGAACACAAACGTAAATCAATAAAATTCCATCAATCCCTCATTTATAA |
| 520 | AGGACAAGAGGTGAAGTGCAAATACAGAACGCAAACGTAAATCAAGAAAATTCCATTAATCCCCCATTTATAA |
| 521 | AGGACGAGGAGTCGAGTGCTA--ACAGAATGCAAACGTAAATCAATAAAACCTACCTAACCCCCCATATATCA |
| 522 | AGGACGAGGAGTCGAGTGCAAATACTGA-CGCAAACGTAAATCAATAAAACTTCATTAATCCCCCATATATAA |
| 523 | AGTACAAGAAGTGAAGCGCAAATACTGA-CGCAAACGTAAATCAATAA-ATTCCATTAATCCCCCATTTATAA |
| 524 | AGGACGAGGAGTCAAGTGCAAATACTGAATGCAAACGTAAATCAATAAAATTCCATTAATCCCCCATATATAA |
| 525 | GGGACAAAAAATGATGTGCAAATACAAAACACAAACGTAAATCAATAAGATTCCATTAATCCCCCATTTATAA |
| 526 | AGGACAAGAAGTGAAGTGCAAATACAGAACAAAAACGTAAATCAATAAAATTCCATTAATCCCCCATTTATAA |
| 527 | AGGACGAGGTGTCGAGTGCTA--ACAGAATGCAAACGTAAATCAATAAAACCTACCTAACCCCCCATATATAA |
| 528 | AGGACGAGGGGTCAAGTGCAAATACAGAATGCAAACGTAAATCAATAAAACTTCATTAATCCCTCATTTATAA |
| 529 | AGGACAAGAAGTGAAGTGCAAATACTGAACGCAAACGTCAATCAATAAAATTCCATTAATCCCCCATTTATAA |
| 530 | AGGACGAGGAGTCGAGTGCTA--ACAGAATGCAAACGTAAATTAACAAAACCTACCTAACCCCCCATATATAA |
| 531 | ACGACGA-GAGTCGAGTGCAGATGCAGAATGCGGGTGTAAATCAATAAAACTTCATTAGTCCCTCATTTGTGG |
| 532 | AGGACAAGAAGCGAAGTGCAAATACAGAACGCAAACGTGAATCAATAAAATTCCATTAAGCCCTCATTTATAA |
| 533 | AGGACGAGGAGTCGAGTGCAA--ACAGTATGCAAACGTAAATCAATAAAACTTCATTAATCCCCCATATATAA |
| 534 | AGGACAAGAAGTGAAGTGCAAATACAGAATGCAAACGTAAATCAATAAAACTCCATTAATCCCCCATTTATAA |
| 535 | GGGACAAGAAATGAAGTGCAAATACAAAACACAAGCGTAAATCAATAAAATTCCATTAATCCCCCATTTATAA |
| 536 | ACGACGAGGAGTCGAGTGCAAATACAGAATGTAAATGTAAATCGATAAAACTTCATTAGTCCCTCATTTATAA |
| 537 | AGGACAAGAAGTGAAGCGCAAATACTGTACGCAAACGTAAATCAATAAAATTCCATTAATCCACCATTTATAA |
| 538 | AGGACGAGGAGTCGAGTCCTA--ACAGAATGCAAACGTAAATCAATAAAACTTCATTAACCCTCCATATATAA |
| 539 | AGGACGAGGAGTCAGGTGCAAATACGGAGTGCAGGCGTAAATCAATAAAACTTCATTAGTCCCCCATATATAA |
| 540 | AGGACAAGAAGTGAAGTGCGAATAGTGAACGCAAACGTAAATCAATAAAATTCCATTAATCCCCCATTTATGA |
| 541 | ACGACGAGGAGTCGAGTGCAA--ACAGAATGCAAACGTAAATCAATAATACTTCATTAATCCCCCATATATAA |
| 542 | AGGTCGAGGAGTCAAGTGCAAATACAGAATGCAAACGTAAATCAATAAAACTTCATTAATCCCCCATATATAA |
| 543 | AGGACAAAAAGTGAAGTGCATATACAGAACACAAACGTAAATCAATAAA-TTCCATTAATCCCCCAT-TATAA |
| 544 | AGGACAAAAAGTGAAGTGCATATACAGAACGCAAACGTAAATCAATAAAATTCCATTAATCCCCCATTTATAA |
| 545 | AGGACAAGAATTGAAGTGCAAATACAGAACACAAACGTAAATCAATAAAATTCCATTAATCCCCCATTTATAA |
| 546 | AGGACAAGAAGTGAAGTGCAAATACTGAACGCAAACGTAAATCAATAACACTCCATTAATCCCCCATTTATAA |
| 547 | AGGACGAGGAGTCGAGTGCTA--TCAGAATGTGAACGTAAATCAATAAAACTTCATTAACCCCCCATATATAA |
| 548 | AGGATAAGAAGTGAAGTGCAAATACAGA-CGCAAACGTAAATCAATAAAATTCCCTTAATCCCCCATTTATAA |
| 549 | AGGACAAGAAGTGAAGTGCAAATACTGAATGCAAACGTAAATCAACAAAATTCCATTAATCCCCCATTTATAA |
| 550 | AGGACAAGAAGTGAAGTGCAAATACAGAATGCAAACGTAAATCAATAAAATTCCATTAATCCCTCATTTATAA |
| 551 | AGGACGAGGAGTCGAGTGCTA--ACAGAATGCAAACGTAAATCGATAAAACCTACCTAACCCCCCGTATATAA |
| 552 | AGGACGAGGAGTCAAGTGCTA--ACAGAATGCAAACGTAAATTAATAAAACCTACCTAACCCCCCATATATAA |
| 553 | AGGACGAGGAGTTGAGTGCTA--ACAGAATGCAAACGTAATTCAATAAAACTTCATTAACCCCCCATATATAA |
| 554 | AGGACGAGGAGTCGAGTGCTA--ACAGAATGCAAACGTAAATCAATAAGACTTCATTAACCCCCCATATATAA |
| 555 | AGGACGAGGAGTCGCGTGCTA--ACAGAATGCAAACGTAAATCAATAAAACCTACCTAACCCCCCATATATAA |
| 556 | AGCACAAGAAGTGAAGTGCAAATACAGAACCCAAACGTAAATCAATAAAATTCCATTAATCCCTCATTTATAA |
| 557 | AGGACGAGAAGTCGAGTGCTA--ACAGAATGCAAACGTAAATCAGTAAAACCTACCTAACCCCCCATATATAA |
| 558 | AGGACGAGGAGTCGAGTGCAA--ACTGAATGCAAACGTAAATCAATAAAACTTCATTGATCCCCCATATATAA |
| 559 | AGGACGAGGAGTCAAGTGCAAATACAGAACGCGAATGTAAATCAATAAAACTTCATTAATCCCCCATATATAA |
| 560 | AG-ACGAGAAGTGAAGT-CAAATACAGAATGCAAACGTAAATCAATAAAATTCCATTAATCCCCCATTTATAA |
| 561 | AGGACAAGAAGTGAAGTGCAAATACTGAATGCAAACGTAAATCAATAAAATTCCATTAACCCCCCATTTATAA |
| 562 | AGGACGGGGAGTCGAGTGCTA--ACAGAATGCAAACGTAAATCAATAAAACCTCATTAACCCCCCATATATAA |
| 563 | AGGACAAAAAGTGAAGTGCAAATATAGAACACAAACGTAAATCAACACAATTCCATTAATCTTCCATTTATAA |
| 564 | AGGACAAGAAGTCAAGTGCAAATACAGAACGCAAACGTAAATCAATAAAACTCCATTAATCCCTCATGTATAA |
| 565 | AGGACAAAAAGTGAAGTGCGAATATAGAGCACAAACGTAA--CAGTAAAATTCTATTAATCTCACATTTATAA |
| 566 | AGGACGAGAAGTCAAGTGCAAATACAGAACACGAACGTGAATCAATAAAATTCCATTAATCCCTCATTTATAA |
| 567 | AGGACAAGAAGTGAAGTGCAAATACTGAACGCAAACGTAAATCAATAAAATTCCATTAACCCCCCATTTATAA |
| 568 | AGGACAAGAAGTGAAGTGCAAATAATGAACGCAAACGTAAATCAATAAAATTCTATTAATCCCCCATTTATAA |
| 569 | AGGACAAGAAGTGAAGCGCAAATACTGTACGCCAACGTAAATCAATAAAATTCCATTAATCCCCCATTTATAA |
| 570 | ACGACGAGGAGTCGATTGCAAATACAGAATGCAAATGTAAATCAATAAAACTTCATTAGTCCCTCATTTATAA |
| 571 | AGAACAGGAAGTCAAGTGCAAATACAGAACACAAACGTAAATCAATAAAATTCCATCAATCCCTCATTTATAA |
| 572 | AGGACAAGAAGTGAAGTGCAAATACAGAACACAAACGTAAATCAATAAAGTTCCATTAATCCCTCATTTATAA |
| 573 | ACGACGAGGAGTCGGGTGCAAATACAGAATGCAAATGTAAATAAATAAAACTTCGTTAGTCCCTCATTTATAA |
| 574 | AGGACAAAAAGTGAAGTGCAAATATAGAACACAAACGGAAATCAATAAAATTCCATTAATCTCCCATTTATAA |
| 575 | AG-ACGAGGAGTCGAGTGCTA--ACAGAATGCAAACGTAAATCAATAAAACCTACCTAACCCCCCATATATAA |
| 576 | ACGACGAGGAATCGAGTGCAAATACAGAATGCAAATGTAAATCAATAAAACTTCATTAGTCCCTCATTTATAA |
| 577 | AGGACGAGGAGTCGAGTGCTA--ACAGAATGCAAACGTAAATCAATAAAACCTACCTAACCCCCCGTATATAA |
| 578 | AGGACAAGAAGTGAGGCGCAAATACTGTACGCAAACGTAATTCAATAAAATTCCATTAATCCCCCATTTATAA |
| 579 | AGGACAAAAAGTGAAGTGCAAATACAG--CACAAACGTAAATCAATAAAATTCCATTAATCTCCCATTTATAA |
| 580 | AGGACGAGGAGTCGAGTGCTA--ACAGAATGCAAACGTAAATCAATAAAACCTACCTAACCCCCTATATATAA |
| 581 | AGGACGAGGAGTCAAGTGCAAATACAGAATGCAAACGTAACTCAATAAAACTTCATTAATCTCTCATTTATAA |
| 582 | AGGACGACGAGTCAAGTGCAAATACAGAATGCAAACGTAAATCAATAAAACTTCATTAATCCCCCATATATAA |
| 583 | AGGACGAGGAGTCGAGTGCTA--ACAGAATGCAAACGTAAATCAATATAACTTCATTAACCCCCCATATATAA |
| 584 | AGGACGAGGAGTTGAGTGCTT--ACAGAATGCAAACGTAAATCAATAAAACCTACCTAACCCCCCATATATAA |
| 585 | AGGACGAGGAGTCAAGTGCAA--ACAGAATGTAAACGTAAATCAATAAAACTTCATTAATCCCCCATATATAA |
| 586 | AGGACAAGAAGTGGGGTGCAAATACTGAACGCCGACGTAAATCAATAAAATTCCATTAATCCCCCATTTATAA |
| 587 | AGGACAAGAAGTCAAGTGCAAATACAGAACGCAAACGTAAATCAATAAAATTCCACTAATCCCCCATTTGTAA |
| 588 | AGGACAGGAAGTCAAGTGCAA--ACAGAACACAAACGTAAATCAATAAAATTCCATCAATCCCTCATTTATAA |
| 589 | AGGACAAGAAGTGAAGTGCAAATACAGAATGCAAACGTAAATCAATGAAATTCCATTAATCCCCCATTTGTAA |
| 590 | AGGACAAGAAGTCAAGTGCAAATACAGAATGCAAACGTAAATCAATAAAATTTCATTAATCC-TCATTTATAA |
| 591 | AGGACGAGGAGTCGAGTGCAA--ACAGAATGCAAACGTAAATCAATAAAGCTTCATTAATCCCCCATATATAA |
| 592 | AGGACAAGAAGTGAAGTGCAAATACAAAACACAAACGTAAATCAATAAAATTCCATTAATCCCTGATTTATAA |
| 593 | AGGACAAGGAGTGAAGTGCAAATACAAAACACAAACGTAAATCAAGAAAATTCCATTAATCCCCCATTTATAA |
| 594 | AGGACGAGGACTCGAGTGCTA--ACAGAATGCAAACGTAAATCAATAAAACTTCATTAACCCCCCATATATAA |
| 595 | AGGACAAGAAGTCTAGTGCAAATACAGAACGCAAACGTAAATCAATAAAATTCCATTAATCCCTCATTTATAA |
| 596 | AGGACGAGAAGTGAAGTGCAAATACAGAATGCAAACGTAAATCAATAAAACTCCATTAATCCCCCATTTGTAA |
| 597 | AGGACAAGAAGTGAAGTGCAAGTACTGAACGCAAACGTAAATCAATAAAATTCCATTAATCCCCCATTTATAA |
| 598 | AGGACGAGGAGTCGAGTGCTA--ACAGAATGCAAACGTAAATCAATAAAACCTACCTAACCCCCCCTATATAA |
| 599 | AGGACGAGGAGTCGAGTGCTA--ACAGAATGCAAACGTAAATCAATAAA-CCTACCTAACCCCCCATATATAA |
| 600 | AGGACAAGAAGTGAGGTGCAAGTACAGAACACAAACGTAAATCAATAAAATTCCATTAATCCCCCATTTATAA |
| 601 | GGGACAAAAAGTGAAGTGTAAATACAAAACACAAACGTAAATCAATAAAATTCCATTACTCCCCCATTTATAA |
| 602 | AGGACGAGGAGTCAAGTGCAA--ACAGAATGCAAACGTAAATCAATAAAACTTCATTAATCACCCATATATAA |
| 603 | AGGACGAGGAGTCGAGTGCTA--ACAGAATGCAAATGTAAATCAATAAAACTTCATTAGTCCCTCATTTATAA |
| 604 | AGGACAAGAAGTGAGGTGCAAATACTGAACGCAAACGTAAATCAATAAAATTCCATTAATCCCCCATCTATAA |
| 605 | AGGACAAGAAGTGAAGTGCAAATACAGAACGCAAACGTAAATCAA-AAAATTCCATTAATCCCCCATTTATAA |
| 606 | AGGACGAGAAGTGAAGCGCAAATACTGTACGCAAACGTAAATCAATAAAATTCCATTAATCCCCCATTTATAA |
| 607 | AGGACGAGAAGTGAAGTCCAAATACA---TGCAAACGTAAATCAATAAAATTCCATTAATCCCCCATTTATAA |
| 608 | AGGACAAGAAGTAAAGTGCAAATACAGAACGCAAACGTAAATCAATAAAATTCCATTAATGCCCCGTTTATAA |
| 609 | ACGACGAGGAGTTGAGTGCAAATACAGAATGCAAATGTAAATCAATAAAACTTCATTAGTCCCTCATTTATAA |
| 610 | GGGATAAGAAATGAAGTGCAAATACA--ACACAAACGTAAATCAATAAAATTCTATTAATCCCTCATTTATAA |
| 611 | AGGACAAGAAGTGAAGCGCAAATACTGTACGCAAACGTAAATCCATAAAATTCCATTAATCCCCCATTTATAA |
| 612 | AGGACATGAAGTGAAGTGCAAATACTGAACGGAAACGTGAATCAATAAAATTCCATTGGTCCCCCATTGATAA |
| 613 | AGGACGAGGAGTCGAGTGCTA--ACAGAATGCAAACGTAAATCAATAAAACCTACC--ACCCCCCATATATAA |
| 614 | AGGACGACGAGTCAAGTGCAA--ACAGAATGCAAACGTAAATCAATAAAACTTCATTAACCCCCCATATATAA |
| 615 | AGGACGAGGAGTCAAGTGCAA--ACAGAATGCAAACGTAAATCAATAAAACTTCATTAATCCCCCCTATATAA |
| 616 | AGGACGAGAAGTGAAGTGCAAAGACAGAACGCAAACGTACATCAAGAAAATTCCATTAATCCCCCATTTATAA |
| 617 | AGGACAAGAAGTGAAGTGCAAATACAGAACGCAAACGTAAATCAATAAAATTCCATTAATCCCCCATTTCTAA |
| 618 | GGGACGAGAAGTGAAGTGTAAATACAAAACACAAACGTAA-TCAATAAAATTCCATTACTCCCCCATTTATAA |
| 619 | GGGACAAGAAGTGAAGTGTAA--ACA--ACACAAACGTAATTCAATAAAATTCCATTACTCCCCCATTTATAA |
| 620 | AGGACGAGGAGTCGAGTGCTA--ACAGAATGCAAACGTAAATCAATAGAACCTACCTAACCCCCCATATATAA |
| 621 | AGGACAAGAAGTTAAGCGCAAATACTGTACGCAAGCGTAAATCAATAAAATTCCATTAATCCCCCATTTATAA |
| 622 | AGGACGAGGAGTCGAGTGCTA--ACAGAATGCAAACGTAAGTCAATAAAACTTCATTAACCCCCCATATATAA |
| 623 | AGGACGAGAAGTGAGGTGCAAATACAGAACACAAACGTAAATCAATAAAATTCCATTAATCCCCCATTTATAA |
| 624 | GGGACAAGAAGTGAAGTGTAAATACAAAACACAAACGTAAATCAATAAAATTCCATTACTCCCCCGTTTATAA |
| 625 | AGGACAAGAAGTCAAGTGCAAATACAGAACACAAACGTAAATCAAGAAAATTCCATTAATCCCCCATTTATAA |
| 626 | AGGACAAGAAGTGAAGCGCAAATACAGAACGCAAACGTAGATCAATAA-ATTCCATTAATCCCCCATTTATAA |
| 627 | AGGACAAGAAGTGAAGCGCAAATACAGAACACAAACGTAAATCAATAAAATTCCATTAATCCCCCATTTATAA |
| 628 | AGGACAAGAAGTGAAGTGCAAAAACAGAACACAAACGTAAATCAATAAAAATCCATTAATCCCTCATTTATAA |
| 629 | AGGACAAGAAGTCAGGTGCAAATACAGACTGCAAACGTAAATCAATAAAACTTCATTAATCCCTCATTTATAA |
| 630 | AGGACAAGCAGTGAAGTGCAAATACAGAACGCAAATGTAAATCAATAAAATTCCATTAATCCCTCATTTATAA |
| 631 | AGGACGTGGAGTCAAGTGCAAATACAG--TGCAAACGTAAATCAATAAAACTTCATTAATCCCCCATATATAA |
| 632 | AGGACAAGAAGTGAAGTGCAAACACAGAATGCAAACGTAAATCAATAAAATTCCATTAATCCCCCATTTATAA |
| 633 | AGGACAAGAAGTGAAGTGCAAATGCTGAACGCAAACGTAAATCAGTAAAATTCCATTAATCCCCCATTTATAA |
| 634 | AGGACGAGAAGTTGAGTGCTA--ACAGAATGCAAA-GTAAATCAATAAAACTTCAT--ACCCCCCATATATAA |
| 635 | AGGACGAGGAGTTGAGTGCTA--ACAGAATGCAAACGCAAATCAATAAAACTTCATTAACCCCCCATATATAA |
| 636 | AGGACAAGAAGTGAAGTGCAAATACAGAACGCAAACGTAAATCGAGAAAATTCCATTAATCTCCCATTTATAA |
| 637 | AGGACAAGAAGTGAGGTGCAAATACAGAACACAAACGTAAATCAATAAAATTCCATCAATCCCCCATTTATAA |
| 638 | AGGACAAGAAGTGAAGTGCAAATACTGAACGCAAATGTGAATCAATAAAATTCCATTAATCCCCCATTTATAA |
| 639 | GGGACAAGAAGTGAAGTGCAAATACA--ACACAAACGTAAACCAATAAAATTCCATTACTCCCCCATTTATAC |
| 640 | AGGGCGAGGAGTCGACTGCTA--ACAGAATGCAAACGTAAATCAATAAAACCTACCTAACCCCCCATATATAA |
| 641 | AGGACAAGAAGTTGAGTGCAAATACAGAACACAAACGTAAATCAATAAAACTCCATTAATCCCCCATTTATAA |
| 642 | AGGACGAGGAGTCGAGTCCTA--ACAGAATGCAAACATAAATCAATAAAACTTCATTAACCCCCCATATATAA |
| 643 | AGAACAAGAAGTGAAGTGCAAATACAGAACGCAAACGTAAATCGGGAAAATTCCATTAATCCCCCATTTATAA |
| 644 | AGGACAAGAAGTGAAGTGCAAATACTGAACGCAAACGTAAATCAATAAAACTCCATTAATCCCCCATTTATAA |
| 645 | AGGACAGGAAGTCAAGTGCAAATACAGA-CGCAAACGTAAATCAATAAAATTCCATCAATCCCTCATTTATAA |
| 646 | AGGACGAGGAGTCGAGTGCTA--ACAGAATGCAAACGTAAATCAATAAAACTTCATTAATCCCCTATATATAA |
| 647 | AGGATGAGAAGTCAAGTGCAAATACAGAATGCAAACGTAAATCAATAAAATTCCATTAATCCCTCATTTATAA |
| 648 | AGGACAAGAAGTGAAGTGCAAATACTGAACGCAAACGTGAATCAA-AAAATTCCATTAATCCCCCATTGATAA |
| 649 | AGGTCGAGGAGTCGAGTGCTA--ACAGAAAGCAAACGTAAATCAATAAAACCTACCTAACCCCCCATATATAA |
| 650 | AGGACAAGAAGTGAAGTGCAAGTACTGAACGCAAACGTAAATCAATAAAATTCCATTAATCCCCCATTGATAA |
| 651 | AGGACGAGGAGTCGGGTGCAA--ACAGAATGCAAACGTAAATCAATAAAGCTTCATTAACCCCCCATATATAA |
| 652 | AGGACGAGAAGTGAAGTCCAAATACAGAACGCAAACGTAAATCAAGAAAATTCCATTAATCCCCCATTTATAA |
| 653 | AGGACGAGGAGTCGAGTGCTA--ACAGAATGCAAACGTAAATCAATAAGACTTCATTGACCCCCCATATATAA |
| 654 | AGGACAAGAAGTGAGGTCCAACTACAGAAGGCAAACGTAAATCAATAAAATTCCATTAATCCCCCATTTATAA |
| 655 | AGAACAAGAAGTCAACTGCAA--ACAGAACGCAAACGTAATTCAATAAAATTCCATCAATCCCTCATTTATAA |
| 656 | AGGACGAGGAGTTGAGTGCTA--ACAGAATGAAAACGTAAATCAATAAAACTTCATTAACCCCCCATTTATAA |
| 657 | AGGACAAGGAGTGAAGTGCAAATACAGAACGCAAACGTAAATCAATAAAGTTCCATTAATCCCCCATTTATAA |
| 658 | AGGACGAGGAGTCCAGTGCTA--ACAGAATGCAAACGTAAATCAATAA-ACTTCATTAACCCCCCATATATAA |
| 659 | AGGACGAGGAGTCAAGTGCAA--ACAGAATGCAAACGTAAGTCAATAAAACTTCATTAATCCCCCATATATAA |
| 660 | AGGACAAGAAGTGAAGTGCAAATACTG--CGCAAACGTGAATCAATAAAATTCCATTAATCCCCCATTTATAA |
| 661 | AGAACAGGAAGTCAAGTGCAA--ACAGAACGCAAGCGTAAATCAATAAAATTCCGTCAATCCCTCATTTATAA |
| 662 | AGGACGAGGGGTCGAGTGCAA--ACAGAATGCAAACGTAAATCAATAAAACTTCATTAATCCCTCATATATAA |
| 663 | AGGACGAGGAGTCGAGTGCTA--ACAGAATGCAGGCGTAAATTAATAAGACCTATCTAACCCCCCATATATAA |
| 664 | AGGACAAGAAGTGAAGTGCAAATACTGAACGCAAACGTAAATCAATAAAATTCCATTAATCCTCCATTTATAA |
| 665 | AGGACGAGGAGTCGAGTGCTA--ACAGAACGCAAACGTAAATCAATAAAACTTCATTAACCCCCCATATATAA |
| 666 | AGGACGGGGAGTCAAGTGCAA--ACAGAATGCAAACGTAAATCAATAAAACTTCATTAATCCCTCATTTACAA |
| 667 | AGGACAAGAAGTGAAGTGCAAATACAGA-CACAAACGTAAATCGATGAAATTCCATTAATCCCCCATTTATAA |
| 668 | AGGACGGGGAGT-GGGTGCAA--ACAGAATGCAAACGTAAATCAATAAAACTTCATTAATCCCCCATATATAA |
| 669 | AGGACAAGAAGTGGAGTGCAAATACAGAACGCAAACGTAAATCAATAAAATTCCATTAACCCCTCATTTATAA |
| 670 | AGGACGAGGAGTCAAGTGCTA--ACAGAATGCAAACGTAAATCAATAAAACTTACCTAACCCCCCATATATAA |
| 671 | AGGACGAGGAGTCGAGTGCTA--ACGGAATGCAAACGTAAATCAATAAAACTTCATTAACCCCCCATATATAA |
| 672 | AGGACAAGAAGTGAAGTGCAAATACTGAAAGCAAACGTGAATCAATAAAATTCCATTAATCCCCCATTTATAA |
| 673 | AGGATAAGAAGTGAGGTGCAAATACAGAACGCAAACGTAAATCAATAAAATTCCATTAATCCCCCATTTATAA |
| 674 | AGGACGGGGAGTCGAGTGCTA--ACAGAATGCAAACGTAAATCAATAAAACTTCATTAACCCCCCATATATAA |
| 675 | GGGACAAGAAGTGAAGTGCAAATACA--ACACAAACGTAAATCAATAAAATTCCATTACTCCCCCATTTGTAA |
| 676 | AGGTCAAGAAGTGAAGTGCAA--ACAGAACACAAATGTAAATCAATAAAATTCCATTAATCCCCCATTTATAA |
| 677 | ACGACGAGGAGTCGAGTGCTA--TCAGAATGCAAACGTAAATCAATAAAACTTCATTAACCCCCCATATATAA |
| 678 | AGGACGAGGAGTGAAGTGCAA--ACAGAATGCAAACGTAAATCAATAAAACTTCATTAATCCCCCATATATAA |
| 679 | ACGACGAGGAGTCGAGTGCTA--ACAGAATGCAAACGTAAATCAATAAAACTTCATTAACCCCCCATATATAA |
| 680 | AGGACAAGAAGTGAAGTGCAAATACTGAACGCAAACGTGAATCAATAAAATTCCATAAATCCCCCATTGATAA |
| 681 | AGAACAGGAAGTCAAGTGCAAATACAGAACGCAAAAGTAAATCAATAAAATTCCATCAATCCCTCATTTATAA |
| 682 | AGGACAAGAAGTGAAGTGCAAATACAGAATGCAAACGTAAATCAATAAAATTCCATTGATCCCCCATTTATAA |
| 683 | AGGACAAGAGGTGAAGTGCAAATACAGAACACAAACGTAAATCAAGAAAATTCCATTAATCCCACATTTATAA |
| 684 | AGGACAAGAAGTGAAGTACAAATAC-GAACACAAACGTAAATCAATAAAATTCCATTAATCCCCCATTTATAA |
| 685 | AGGACAAGAAGTGAAGTGCAAATACAGAACGCAAACGTAAATCAATAAAATTCCATTAATCCCCCATTTATAG |
| 686 | AGGACAAGAAGTGAAGTGCAAATATAGAACGCAAACGTAAATCAATAAAATTCCATTAATCCCCCATTTATAA |
| 687 | AGGACGA-GAGTCGGGTGCTA--ACAGAATGCAAACGTAAATCAATAAAACTTCATTAACCCCCCATATATAA |
| 688 | AACACGAGAAGTGAAGTCCAAATACAGAATGCAAACGTAATTCAATAAAATTCCATTAATCCTCCATTTATAA |
| 689 | AGGACAAGAAGTGAAGTTCAAATACAGAACGCAAACGTAAATCAATAAAATTCCATTAATCCCCCATTTATAA |
| 690 | AGGACTAGAAGTGAAGTGCAA--ACTGAACACAAACGTAAAT-AATAAAATTCCATTAATCCCCCATTTATAA |
| 691 | AGGACGAGAAGTGAAGTCCAAATACAGAATGCAAACGTAA-TCAATAAAACTCCATTAATCCCCCAATTATAA |
| 692 | AGGACAAGAAGTGA--TGCAAATGCTGAACGCAAACGTAAATCAATAAAATTCCATTAATCCCCCATTTATAA |
| 693 | GGGACAAGAAGTGAAGTGTAAATACAAAACACAAACGTAAATCAATGAAATTCCATTATTCCCCCATTTATAA |
| 694 | AGGACAAGAAGTGAAGTGGAAATACTGAATGCAAACGTAAATCAGTAAAATTCCATTAATCCCCCATTTATAA |
| 695 | GGGACAAGAAGTGAAGTGCAA-TACAAAACACAAACGTAAATCAATAAAATTCCATTACTCCCCCATTTATAA |
| 696 | AGGACAAGAAGTGAAGTGCAAATACAGAACGCAAACGTAAATCAATAAAATTCCATTAATCCCTGATTTATAA |
| 697 | AGGACAAGAAGTGAAGCGCAAATACTGTACGCAAACGTAAATCAATAAAATTCCATTAATCCCCCATTTATCA |
| 698 | AGGACGAGGAGTGGAGTGCAAAAACAGAATGCAAACGTAAATCAATAAAACTTCATTAATCCCTCATATATAA |
| 699 | AGGACGAGGAGTCGAGTGCAAATACAGAATGCAAACGTAAGTCAATAAAACTTCATTAATCCCCCATATATAA |
| 700 | AGGACGAGAAGTCAAGTGCAAATACAGAATGCAAACGTAAATCAATAAAACTTCATTAATCCCCCATATATAA |
| 701 | AGGACGAGGAGTCAAGTGCAAATACAGAACGCAAACGTAAATCAATAAAATTCCATCAATCCCTCATTTATAA |
| 702 | AGGACAAGAAGTGAAGTGCAAATAGAGAACACAAACGTAAATCAATAAAATTCCATTAATCCCCCATTTATAA |
| 703 | AGGACAAGAAGTG-AGTGCAAATACAGA-CGCAAACGTAAATCAATAAAATTCCATTAATCCCTCATTTATTA |
| 704 | AGGACAAGAAGTGAAGCGCAAATACTG-ACGCAAACGTAAATCAATAAAATTCCATTAATCCCTCATTTATAA |
| 705 | AGGACAAGAAGTCAAGTGCAAATACAGAACGCAAACGTAAA-CAATAAAATTCCATTAATCCCTCATTTATAA |
| 706 | AGGACAAAAAGTGAAGTGCAAATTCAGAACACAAACGTAAATCAATAAAATTCCATTAATCCCCCATTTATAA |
| 707 | AGGACGAGGAGTCAAGTGCAAATGCAGAATGCAAACGTAAATCAATAAAACTTCATTAATCCCCCATATATAA |
| 708 | AGGACAAGAAGTGAAGTGCAA--ACTGAACGCAAACGTAAATCAATAAAATTCCATTAATCCC-CATTTATAA |
| 709 | AGGACAAGAAGTGAAGTGCAAATAC---GCGCAAACGTGATTCAATAAAATTCCATTAATCCC-CATTTATA- |
| 710 | AGGACAAGAAGGGAAGTGCAAATAC---GCACAAACGTAAATCAATAAAATTCCATTAATCCCCCATTTATAA |
| 711 | AGGACAAGAAGTGAAGTGCAAATGCAGAACGCAAACGTAAATCAATAAAATTCCATTAATCCCCCATTTATAA |
| 712 | AGGACGAGGAGTCAAGTGCAA--ACAGAATGCAAACGTAAATCAATAAAACTTCAT--ATCCCCCATATATAA |
| 713 | AGGACAAGAAGTGAAGCGCAAATACTGTACGCAAACGTAAATCAATAAAATTCCATTAATCCCCCATTTATAT |
| 714 | ACGACGAGGAGTCGATTGTAAATACAGAATGCAAATGTAAATCAATAAAACTTCATTAGTCCCTCATTTATTA |
| 715 | AGGACAAGAAGTGAAGTGCAAATACAGAACACAAACGTAAACCAATAAAATTCCATTAATCTCCCATTTGTAA |
| 716 | AGGACAAAAAGTGAAGTGCAAATACAGAACGCAAACGTAAATCAATAAAATTCCATTAATCTCCCATTTATAA |
| 717 | AGAACAAGAAGTGAAGTGCAAATACTGAACGCAAACGTGAATCAATAAAATTCCATTAATCCCCCATTGATAA |
| 718 | AGGACGAGGAGTCGGGTGCAAATGCAGAATGCAAACGTAAATCAATAAAACTTCATTGATCCCTCATTTATAA |
| 719 | AGGACAAGAAGTGAAGTGCAAATACAGAACGCAAACGTAAATCAATAA-ATTCCATTAATCCCTCATTTATAA |
| 720 | AGGACAGGAACTCAAGTGCAAATACAGAATGCAAACGTAAATCAATAAAATTCCATCAATCCCTCATTTATAA |
| 721 | AGGACAAGAAGTGAAGTGCAAATACAGAACGCAAACGTAAATCAATAAAATTCCATTAATCCCTCGTTTATAA |
| 722 | AGGATAAGAAGTGGAGCGCAAATACTGTACGCAAACGTAAATTAATAAAATTCCAATAATCCCCCATTTATAA |
| 723 | AGGACAAAAAGTGAAGTGCAAACATAGAGCACAAACGTAACCCAGTAAAATTCCATTAATCTCCCATTTATAA |
| 724 | GGGACAAGAAGTGAAGTGCAACCACAAAACATAAACGTAAATCAATAAAATTCCATTACTCCCCCATTTATAA |
| 725 | AGGACAAGAAGTGAAGTGCAAATACTGAACGCAAACGTAAATCAATAAAATCCCATTAATCCCCCATTTATAA |
| 726 | AGGACAAGAAGTGAAGTGCAAATACAGAACACAAACGCAAATCAATAAAATTCCATTACTCCCCCATTTATAA |
| 727 | AGCACGAGGAATCGAGTGCTA--ACAGAATGCAAACGTAAATCAATAAAACTTCATTAACCCCCCATATATAA |
| 728 | AGGACAAGAAGTGAAGTGCAAATACAGAACACAAACGTAAATCGA-AAAATTCCATTAATCCCCCATTTATAA |
| 729 | AGGACGAGGAGTCGAGTGCTA--ACAGAATGCAAATGTAAATCAATAAAACTTCATTAACCCCCCATATATAA |
| 730 | ACGACGAAGAGTCGAGTGCAGATGCAGAATGCGGGTGTAAACCAATAAAACTTCATTAGTCCCTCATTTGTGG |
| 731 | AGGACGAGGAGTCGAGTGCAAATACAGAATGCAAATGTAAATCAATAAAATTTCATTAGTCCCTCATTTATAA |
| 732 | AGGACAAGAAGTGAAGTGCAAATACAGAGCGCAAACGTAAATCAAGAAAATTCCATTAATCCCCCATTTATAA |
| 733 | AGGACAACAAGTGGAGTGCAAATACAGAATGCAAACGTAAATCAATAAAATTCCATTAATCCTTCATTTATAA |
| 734 | AGGACAAGAAGTGAAGTGCAAATACTGAATGCAAACGTAAATCGATAAAATTCTATTAATCCCCCATTTATAA |
| 735 | AGGACGAGGAGTCAAGTGCAAACACAGAATGCAAACGTATATCAATAAAACTTCATTAATCCCCCATATATAA |
| 736 | AGGACAAGAAGTGAAGTGCAAACACAGAACACAAACGTAAATCAATAAAATTCCATTAATCCCCCATTTATAA |
| 737 | AGGACGAGGAGTCGAGTGCTA--ACAGAATGCAAACGTAAATCAATAAAACTTCATTAACCCTCCATATATAA |
| 738 | AGGACAAGAAGTGAAGTGCAAATACAGAACGCAAACGTAAATCAAGAAAA-TCCATTAATCCCCCATTTATAA |
| 739 | GGGACAAGAAGTGAAGTGTAAACACAAAACACAAACGTAAATCAA-AAAATTCCATTACTCCCCCATTTATAA |
| 740 | AGGACGAGGAGTCGAGTGCTA--ACAGAATGCAAACGTAAATCAATAAAACTTCATTAATCCC-CATATATAA |
| 741 | AGGACGAGAAGTGAAGTCCAAATATAGAATGCAAACGTAAATCAATAAAATTCCATTAATCCCCCATTTATAA |
| 742 | AGGACAAGAAATGAAGTCCAAATACAGAACGCAAACGTAAATCAAGAAAATTCCATTAATCC--CATTTATAA |
| 743 | AGGACATGAAGTGAAGTGCAAATACTGAACGCAAACGTAAATCAATAAAATTCCATTAATCCCCCATTTATAC |
| 744 | AGGACAAGAAGTGAAGCGCAAATACAGAACGCAAACGGAAATCAATAAAATTCCATTAATCCCCCATTTATAA |
| 745 | GCGACGAGGAGTCGAGTGCAAATACAGAATGCAAATGTAAATCAATAAAACTTCATTAGTCCCTCATTTATAA |
| 746 | AGGACGAGGAGTCGGGTGCTA--ACAGGATGCAAACGTAAGTCGATAAAACTTCATTAACCCCCCATATATGA |
| 747 | AGGACAAGAAGTGAGGTGCAAATACAGAACGCAAACGTAAATCAAGAAAATTCCATTAATCCCCCATTTATAA |
| 748 | AGGACGAGGAGTCGAGTGCCA--ACAGAATGCAAACGTAAATCAATAAAACCTACCTAACCCCCCATATATAA |
| 749 | AGGACGAGGAGTCGAGTGCTA--ACAGAATGCAAACGTAAATCAATAAAACCTAACTAACCCCCCATATATAA |
| 750 | AGGACGAGGAGTCGGGTGCAAATACAGAATGCAAACGTAAATCAATAAAACTTCATTAATCCCCCATATATAA |
| 751 | AGGACAAGAAGTGAAGTGCGAATACAGAACGCAAACGTAAATCAAGAAAATTCCATTAATCCCCCATTTATAA |
| 752 | AGGACAAGGAGTCGAGTGCAGATGCAGAATGCGGGCGTAAATCAATAAAACTTCATTAGTCCCTCATTTGTGG |
| 753 | AGGACGAGTAGACAAGTGCAAATACAGAATGCAAACGTAAATCAATAAAACTTCATTAATCCCCCATATATAA |
| 754 | AGGACGAGGAGTCGAGTGCTA--ACTGAACGCAAACGTAAATCAATAAAACCTACTTAACCCCCCATATATAA |
| 755 | AGGACAAAAAGTGAAGTGCGAATACAGAACACAAACGTAAATCAATAAAATTCCATTAATCCCCCATTTATAA |
| 756 | AGGACGAGGAGTCAAGTGCAA--ACAGAATGCAAACGTAAATCAATAAAACTTCAGTAATCCCCCATATATAA |
| 757 | AGGACAAGAAGTGAAGTGCAAATACAGAAGGCAAACGTGAATCAATAAAACTCCATTAATCCCCCATTTATAA |
| 758 | AGGACAAGAAGCGAAGTGCAAATACAGAACGCAAACGTAAATCAATAAAATTCCGTTAATCCCTCATTTATAA |
| 759 | AGGTCGAGGAGTCGAGTGCTA--ACAGAATGCAAACGTAAAT-AATAAAACCTACCTAACCCTCCATATATAA |
| 760 | AGGACAAGAAGTGAAGCGCAAATACTGTATGCAAACGTAAATCAATAAAATTCCATTAATCCCCCATTTATAA |
| 761 | AGGACAGGAAGTGAAGTGTAAATAAAGAACATAAACGTAAATCAATAAAATTCCATTAATGCCCCATTTATAA |
| 762 | AGGACAGGAAGTCAAGTGCAAATACTGAACGCAAACGTAAATCAATAAAATTC-ATCAATCCCTCATTTATAA |
| 763 | ACGACGAGGAGTCGAGTGCAAATACAGAATGCAAATGTAAATCAATACAACTTCATTAGTCCCTCATTTATAA |
| 764 | AGGACGAGGAGTCGAGTGCTA--ACAGAATGCAAACGTAAA-TAATAAAACCTACCTAACCCCCTATATATAA |
| 765 | AGGACAAGAAGTGAAGTGCAAATGCTGAACACAAACGTAAATCAATAAAATTCCATTAATCCCCCATTTATAA |
| 766 | AGGACAAGAAGCCAAGTGCAAATACAGAACACAAACGTAAATCAATAAAATTCCATTAATCCCTCATTTATAA |
| 767 | AGAACAGGAAGTCAAGTGCAAATACAGAATGCAAACGTAAATCAATAAAATTCCATCAATCCCTCATTTATAA |
| 768 | AGGACAAGAAGTGAAGTGCAA--ACAGAACGCAAACGTAAATCAATAAAATTCTATTAATCCCCCATATATAA |
| 769 | AGGACAAGAAGTGAAGTGCAAATACTGAACACAAACGTAAATCGATAAAATTCCATTAATCCCCCATTTATAA |
| 770 | AGGACGAGAAGTCAAGTGCAAATACAGAACGCAAACGTAAATCAATAAAATTCCATCAATCCCTCATTTATAA |
| 771 | AGGACGAGGAGTGGAGTGCTA--ACAGAATGCAAACGTAAATCAATAAAACTTCATTAACCCCCCATATATAA |
| 772 | AGGACAAGAAGTGAAGTGCAAATACAGAATGCAAACGTAAATCAATAAAATTCCGTTAATCCCTCATTTATAA |
| 773 | GGGACAAGAAGTGAAGTGTAA--ACAAAACACAAACGTAAATCAATAAAATTCCGTTACTCCCCCATTTATAA |
| 774 | AGGACGAGGAGTCGAGTGCTA--ACAGAATGCAAACGTAAATCAATAAAACTTCATTAACCCCCCATTTATAA |
| 775 | AGGACGAGAAGTGGAGTGCTAATACAGAACACAAACGTAAATCAATAAAATTCCATTAATCCCCCATTTATAA |
| 776 | CGGACATGAAGTGAAGTGCAAATACAGA-CACAAACGTAAATCAATAAAATTCCATTAATCCACCATTTATAA |
| 777 | ACGACGAGCAGTCGAGTGCAAATACAGAATGCAAATGTAAATCAATAAAACTTCATTAGTCCCTCATTTATAA |
| 778 | AGGACAAGAAGTGAAGTGCAAATACTGAACGCAAACGTAAATCAATAAAGTTCCATTAATCCCCCATTTATAA |
| 779 | AGGACAAGAAGTAAAGTGCAAATACAGAACGCAAACGTAAATCAATAAAATTCCATTAATCCCTCATTTATAA |
| 780 | AGGACAGGAAGTCAAGTGCAAATACAGAATGCAAACGTAAATCAATAAAATTCCATCAATCCCTCATTTATAA |
| 781 | AGGACAAGAAGTGAAGTGCAAACACAGAAT-CAAACGTAA-TCAAGAAAATTCCATTAATCCCCCATTTGTAA |
| 782 | AGGACGAGAGGTGAAGTCCAA--ACAGAATGCAAACGTAAATCAATAAAATTACATTAATCCCCCATTTATAA |
| 783 | AGGACAAGAAGTGAAGTGCAAATACAGAACACGAACGTAAATCAATAAAATTCCATTAATCCCCCATTTATAA |
| 784 | AGGACGAGGAGTCAAGTGCTA--ACAGAATGCAAACGTAAATCAATAAAACCTACCTAACCCCCCATATATAA |
| 785 | AGGACAGGAAGTGAAGTGCAAATACTGAACGCACACGTAAATCAATAAAATTCCATTAATCCCCCATTTATAA |
| 786 | AGGACAAGAAGTGAAGTGCAAATCCAGAGTGCAAACGTAAATCAATAAAATTCCATTAATCCCCCATTGATAA |
| 787 | AGGACGAGGAGTAGAGTGCAAATACAGAATGCAGGCGTAAGTCAGTGAGACTTCATTGGTCCCTCATTTACAG |
| 788 | AGGACGAGGAGTCGAGTGCTA--ACAGA-TGCAAACGTAAATCAATAAAACTTCATTAACCCCCCATATATAA |
| 789 | AGGACGAGGAGTCAAGTGCTA--ACAGAATGCAAACGTAA--CAATAAAACTTCATTAACCCCCCATATATAA |
| 790 | AGGACAAGAAGTGAATTGCAAATACTCAACGCAAACGTAAATCAATAAAATTCCATTAATCCCCCATTTATAA |
| 791 | AGGACAAAAAGTGAAGTGCAAATACAGAACGCAAACGTAAATCCAGAAAATTCCATTAATCCCCCATTTATAA |
| 792 | AGGACAAAAGGTGAAGTGCAAATACAGAACACAAAAGTAAATCAATAAAATTCTATTAATCCCCCATTTATAA |
| 793 | AGGACAAAAAGTGGAGTGCAAATACAGAACGCAAACGTAAATCAATAAAATTTCATTAATCCCTCATTTATAA |
| 794 | AGGACGAGAAGTGGAGTGCTAATACAGAACGCAAACGTAAATCAATAAAATTCCATCAATCCCTCATTTATAA |
| 795 | AGGACAAAAAGTGAAGTGCAAATACAGAATGCAAACGTAAATCGATAAAATTCCATTAATCCACCATTTATAA |
| 796 | AGGACGAGGAGTTGAGTGCTA--ACAGAATGCAAACGTAAATCAATAAAACCTACCTAACCCCCCATATATAA |
| 797 | AGGACAAGAAGTGAGGTGCAAATACAGAACGCAAACGTAGATCAATAAAATTCCATTAATCCCCCATTTATAA |
| 798 | AGGACGAGGAGTCAGGTGCAAATACAGAATGCAAACGTAAATCAATGAAACTTCATTAGTCCCCCATATATAA |
| 799 | AGGACAAGAAGTGAAGTGCAAATACAGAACGCAAACGTAAATCAAGAAGATTCCATTAATCCCCCATTTATAA |
| 800 | AGGACAAGAAGTGAAGTCCAAATACAGAACGCAAACGTAAATCAAGAAAATTCCATTGATCCCCCATTTATAA |
| 801 | GGGACAAGAAGTGAAGTGTAAATACAAAACACGAACGTAAATCAAGAAAATTCCATTACTCCCCCATTTATAA |
| 802 | AGGACAAGAAGTGAAGTGTAAATACAAAACACAAACGTAAATCAATAAAATTCCATTACTCCCCCATTTATAA |
| 803 | AGGACAAGAAGTGAAGTGCAAATACAGAATGCAAACGTAAATCGAGAAAATTCCATTAATCCCCCATTTATAA |
| 804 | AGGACGAGGAGTCGAGTGCTA--ACAGAATGCAGACGTAAATCAGTAAAACCTACCTAACCCCCCATATATAA |
| 805 | AGGCCGAGGAGTCGAGTGCTA--ACAGAATGCAAACGTAAATCAATAAAACCTACCTAACCCCCCATATATAA |
| 806 | AGGACAAGAAGTGAAGTGCAAATACAGAACGCAAACCTACATCAATAAAATTCCATTAATCCCCCATTTATAA |
| 807 | AGGACGAGGAGTCAAGTGCAA--ACAGAATGCGAACGTAAATCAATAAAACTTCATTAATCCCCCATATATAA |
| 808 | AGGACAAGAGGTGAAGTGCAAATACTGA-TGCAAACGTAAATCAATAAAATTCCATTAATCCCCCATTTATAA |
| 809 | AGGACAAGAAGTGAAGCGCAAATACTGTACGCAAACGTAAATCAATAAAATTCCATTAATCC-CCATTTATAA |
| 810 | AGGACAAGAAGTGAAGTGCAAATACTGA-CACAAAAGTAAATCATTAAAATTCCATTAATCCCCCATTTATAA |
| 811 | AGGACAAGAAGTCAAGTGCAAATACAGAATGCAAACGTAAATCAATAAAATTCCATCAATCCCTCATTTATAA |
| 812 | AGGACGAGGAGTGAAGTGCAAATACAGAACACAAACGTAAATCGATAAAGTTCCATTAATCCCCCATTTGTAA |
| 813 | AGGACGAGGAGTCGAGTGCTT--ACAGAATGCAAACGTAAATCAATAAAACCTACCTAACCCCCCATATATAA |
| 814 | AAGACGAGAAGTGAAGTGCAAATACAGAATGCCAACGTAAATCAATAAAATTCCATTAATCCCCCATTTATAA |
| 815 | AGGACAAAAAGTGGAGTGCAAATACAGAACACAAACGTAAATCAATAAAATTCAATTAATCCCCCATTTATAA |
| 816 | CGGACAAGAAGTGGAGTGCAAATACAGA-CGCAAACGTAAATCAATAAAATTCCATTAATCCCCCATTTATAA |
| 817 | AGGACAAGAAGTGAAGTGCAAATACTGAATGCAAACGTAAATCAATAAAATTCCATTAGTCCCCCATTTATAA |
| 818 | AGGACGAGGAGTCAAGTGCAAATACAGAATGCAAACGTAAGTCAATAAAACTTCATTAATCCCTCATTTACAA |
| 819 | AGGACAAGAAGTGAAGTGCAAACACAGAACGCAAACGTAAACCAAGAAAATTCCATTAATCCCCCATTTATAA |
| 820 | AGGACGAGAAGTGAAGTGCAAATACTGAACGCAAACGTAAA-CAATAAAATTCCATTAATGCTGCATTTATAA |
| 821 | AGGACGAGGAGTAGAGTGCTA--ACAGAATGCAAACGTAAATCAATAAAACCTACCTAACCCCCCATATATAA |
| 822 | AGGACAGGAAGTCAGGTGCAAATACAGAACGCAAACGTAAATCAATAAAATTCCATCAATCCCTCATTTATAA |
| 823 | AGGACGACGAGTCAAGTGCAAATACAGAATGCAAACGTAAATCAATAAAATTTCATTAATCCCTCATTTATAA |
| 824 | AGGATAAGAAGTGTAGTGCAAATACTGAACGCAAACGTAAATTAATAAAATTCCATTAATCCCCCATTTATCA |
| 825 | AGGACAAGAAGTGAAGTGCAAATACAGAACGCAAACGTAAATCAATAAAATTCCATTAATCCCTTATTTATAG |
| 826 | AGGACATGAGGTGAAGTGCAAATACAGAACACAAACGTAAATCAATAAAATTCCATTAATCCACCATTTATAA |
| 827 | AGGACGACGAGTCAAGTGCAAATACAGAATGCAAACGTAAATCAGTAAAACTTCATTAATCCCTCATTTACAA |
| 828 | AGGACAAAGAGTGAAGTGCAAATACTGAACACAAACGTAAATCAATAAAATTCCATTAATCCCCCATTTATAA |
| 829 | AGGACAAGAAGTGAAGTGCAAATACAGAACGCAAACGTAAATCAATAAAATTCCATTAATGCCCCATTTGTAA |
| 830 | ACGACGAGGAGTCAAGTGCAA--ACAGAATGCAAACGTAAATCAATAAAACTTCATTAATCCCCCATATATAA |
| 831 | AGGACAAGAAGTGAAGTGCAAATACAG--CGGAAACGTAAATCGAGAAAATTCCATTAATCCC-CATTTATAA |
| 832 | AGGACGAGGAGTTGAGTGCAAATACAGAATGCAAACGTAAATCAATAAAACTTCATTAATCCTTCATTTATAA |
| 833 | GGCACAAGAAATGAAGTGCAG--ACAGAATGGAAACGTAAATCAATAAAATTCCATTAATCCCCCATTTATAA |
| 834 | AGGACAAGAAGTGAAGTGCAG--ACAGAACACAAACGTAAATCAATAAAATTCCATTAATCCGCCATTTATAA |
| 835 | AGGACAAGAAGTGGAGTGCAAATGCAGAACGCAAACGTAAATCAATAAAATTCCATTAATCTCTCATTTATAA |
| 836 | ACGACGAGGAGTCAAGTGCAAATACAGAATGCAAATGTAATTCAATAAAACTTCATTAGTCCCTCATTTATAA |
| 837 | AGGACGAGAAGTGAAGTCCAAATACAGAATGCAAACGTAAATCAAGAAAATTCCATTAATCCCCCATTTATAA |
| 838 | AGGAAGAGAAGTGAAGTCCAAATACAGA-TGCAAACGTAAATCAATAAAATTCCATTAATCCCCCATTTATAA |
| 839 | ACGACGAGGAGTCAAGTGCAAATGCAGAATGCAAATGTAAATCGATAAAACTTCATTAGTCCCTCATTTTTAA |
| 840 | AGGACGAAGAGCCGAGTGCTA--ACAGAATGCAAACGTAAATCAATAAAGGTTCATTAACCCCCCATATATAA |
| 841 | AGGACAAGAAGTCAAGTGCAA--ACAGAACGCAAACGTAAATCAATAAAATTCCATTAATCTCTCATTTATAA |
| 842 | AGGACAAGAAGTGAAGCTCGAATACTGTACGCAAACGTAAAGCAATAAAATTCCATTAATCCCCCATTTATAA |
| 843 | AGGACAAGAAGTGAAGCGCAAA-ACTGTACGCAAATGTAAATCAATAAAATTCCATTAATCCCCCATTTATAA |
| 844 | AGGACAGGAAGTCAAGTGCAAATACAGAATGCAAACGTAAATCAATAAAATTCCATTAATCCCTCATTTATAA |
| 845 | AGGACAAGAGGTGAAGCGCAAATACTGTACGCAAACGTAAATCAATAAAATTCCATTAATCCCCCATTTATAA |
| 846 | AGGACAAGAAGTGAAGTGCAAATACAGAACGCAAACGTAAATCAATAAAACTCCATTAATCCCCCATTTATAA |
| 847 | GTGACAAGAAGTGAAGTGTAAATACA--ACACAAACGTAAATCAATAAAATTCCATTACTCCCCCATTTATAA |
| 848 | AGGACAAAAAGTGAAGTGCAAATATAGAGCACAAACGTAACCCAGTAAAATTCCATTAATCTCCCATTTATAA |
| 849 | AAGACAGGAAGTCAAGTGCAAATACAGAGCGCAAACGTAAATCAATAAAATTCCATCAATCCCTCATTTATAA |
| 850 | AGGACAAGAAGTGAAGTGCAAATACTGTATGCAAACGTAAATCAATAAAATTCCATTAATCCCCCATTTATAA |
| 851 | GGGACAAGAAGTGAAGTGCAAATACAAAACACAAACGTAAATCAATAAAATTCCATTACTTCCCCATTTATAA |
| 852 | AGGGCAAGAAGTGAAGTGCAAATACAGAACGCAAACGTAAATCAATAAAATTCCATTAATCCCCCATCTATAA |
| 853 | AGGGCAAGAAGTGAGGTGCAA--AC--AGCGCAGGCGTAAATCAATAAGATTCCATTAATCCCCCATGTGTAA |
| 854 | AGGACAAGAAGTGAAGTGCAAATACAGAATGCAAACGTAAATCAATAAGACTTCATTAGTCCCCCATATATAA |
| 855 | AGGACGAGGAGTCGAGTGCTA--ACAGAATGCAAACGTAAATCAATAAAACCTCA---ACCCCCCATATATAA |
| 856 | AGGACGAGAAGTGAAGTGCAAATACAGAACGCAAACGTAAATCAATAAAATTCCATTAATCCCCCATTTATAA |
| 857 | AGGACAAGAGGTCAAGTGCAA--ATAGAACACAAACGTAAATCAATAAAACTTCATTAATCCCTCATTTATAA |
| 858 | AGGACAACAAGTGAAGCGCAAATAC--AATGCAAACGTAAATCGAGAAAATTCCATTAATCCCCCATCTATAA |
| 859 | AGGACAAGAAGTGAAGCGCAAATACTGTACGCAAACGTAAATCAATAAAATTCCATTAGTCCCCCATTTATAA |
| 860 | AGGACGAGGTGTCAAGTGCTA--ACAGAATGCAAACGTAAATCAATAAAACTTCATTAACCCCTCATATATAA |
| 861 | AGGACGAGGAGTCGAGTGCTA--GCAGAATGCAAACGTAAATCAATAAAACCTACCTAACCCCCCATATATAA |
| 862 | AGGACAAGAAGTGAAGTGCAAATACAGAACGCAAACGTAAATCAAGAAAATTTCATTAATCCCCCATTTATAA |
| 863 | AGGATAAGAAGTGAAATGCAAATACAGAACGCAAACGTGAATCAATAAAATTCCATTAATCCCCCATTTATAA |
| 864 | AGGACAAAAAGTGAATTGCAAATATAGAACACAAACGTAAATCAATAAAATACCATTAATCTCCCATTTATAA |
| 865 | AGGACGAGGAGTCGAGTGCGA--ACAGAATGCAAACGTAAATCAATAAAACTTCATTAACCCCCCATATATAA |
| 866 | AGGACGAGGAGTCAAGTGCAAATACTGTACGCAAACGTAAATCAATAAAACTTCATTAATCTCCCATTTATAA |
| 867 | AGGACAAGAAGTGAAGTGCAAATACAG--CGCAAACGTAAATCAATAAAATTCCATTAATGCCCCATTTATAA |
| 868 | AGGACAAGAAGTGAAGCGCAAATACTGTACGCAAACGTAAAGCAATAAAATTCCATTAATCCCCCATTTATAA |
| 869 | AGGACGAGGAGTCGGGTGCTA--ACAGAATGCAAACGTAAATCAATAAAACCTACCTAACCCCCCATATATAA |
| 870 | AGGACGAGGAGTCGAGTGCTA--ACAGAATGCAAACGCAGATCAATAAAACTTCATTCACCCCCCATATATAA |
| 871 | GGGACAAGAAGTGAAGTGTAAATACAAA-CACAAACGTAAATCAATAAAATTCCATTACTCCCCCATTTATAA |
| 872 | AGGACAAGAAGTGAAGTGCAAATACTGAACGCAAACGTAAATCAATAAAATTCCATTAATCCCTCATTTATAA |
| 873 | AGGACAAGAAGTGACATGCAAATACAGAACACAAACGTAAATCAATAAAATTCCATTAATCCCCCATTTATAA |
| 874 | AGGACAAAAAGTGAAGTGCAAATGCTGAACGCAAACGTAAATCAATAAAATTCCATTAATCCCCCATTTATAA |
| 875 | AGGCCAAGAAGTGAAGTGCAAATACTGAACGCAAACGTGAATCAATAAAATTCCATTAATCCCCCATCTATAA |
| 876 | AGGACAAGGAGTGAAGTGCAAATACAGAACGCAAACGTAAATCAATAGAATTCCATTAATCCCTCATTTATAA |
| 877 | AGGACAAGAAGTGAAGTGCAAATACAGAATGCAAACGTAGATCAATAAAATTCCATTAATTCCCCATTTATAA |
