# Supplementary material for: Inference of Transposable Element Ancestry
Source: PLoS Genet. 2014 Aug 14;10(8):e1004482. doi: 10.1371/journal.pgen.1004482 (PMC4133154; doi:10.1371/journal.pgen.1004482)
Supplement: Table S2 — MCMC results for LAVA candidate ancestors. (DOCX) [file pgen.1004482.s005.docx]

| Replicative frequency rank | Estimated replicative frequency | Percentile 2.5 of replicative frequency | Percentile 97.5 of replicative frequency | Probability sequence replicated | Ancestral subfamily | Consensus subfamily |
| --- | --- | --- | --- | --- | --- | --- |
| 1 | 130.9 | 111 | 153 | 1 | 3 | 1 |
| 2 | 124.269 | 102 | 147 | 1 | 3 | 1 |
| 3 | 77.855 | 62 | 95 | 1 | 6 | 1 |
| 4 | 76.146 | 61 | 94 | 1 | 2 | 1 |
| 5 | 45.146 | 33 | 58 | 1 | 6 | 1 |
| 6 | 32.934 | 23 | 45 | 1 | 5 | 1 |
| 7 | 30.331 | 20 | 43 | 1 | 2 | 1 |
| 8 | 29.955 | 21 | 40 | 1 | 10 | 1 |
| 9 | 24.909 | 17 | 35 | 1 | 2 | 1 |
| 10 | 22.91 | 15 | 32 | 1 | 10 | 1 |
| 11 | 19.632 | 12 | 29 | 1 | 2 | 1 |
| 12 | 19.215 | 12 | 29 | 1 | 9 | 1 |
| 13 | 18.863 | 11 | 29 | 1 | 9 | 1 |
| 14 | 18.267 | 11 | 27 | 1 | 10 | 1 |
| 15 | 16.079 | 9 | 24 | 1 | 5 | 1 |
| 16 | 15.981 | 8 | 24 | 1 | 6 | 1 |
| 17 | 15.676 | 8 | 25 | 1 | 2 | 1 |
| 18 | 14.868 | 8 | 23 | 1 | 15 | 1 |
| 19 | 14.031 | 8 | 22 | 1 | 9 | 1 |
| 20 | 13.471 | 8 | 21 | 1 | 7 | 1 |
| 21 | 10.748 | 6 | 17 | 1 | 5 | 1 |
| 22 | 10.267 | 5 | 17 | 1 | 8 | 1 |
| 23 | 10.054 | 4 | 18 | 1 | 8 | 1 |
| 24 | 9.74 | 5 | 17 | 1 | 9 | 1 |
| 25 | 9.559 | 5 | 16 | 1 | 9 | 1 |
| 26 | 8.683 | 4 | 16 | 0.999 | 10 | 1 |
| 27 | 8.52 | 4 | 15 | 1 | 5 | 1 |
| 28 | 7.868 | 4 | 14 | 1 | 7 | 1 |
| 29 | 7.781 | 3 | 14 | 1 | 7 | 1 |
| 30 | 7.522 | 0 | 14 | 0.972 | 3 | 1 |
| 31 | 7.151 | 3 | 13 | 0.991 | 2 | 1 |
| 32 | 7.135 | 3 | 14 | 1 | 3 | 1 |
| 33 | 7.076 | 3 | 13 | 1 | 2 | 1 |
| 34 | 6.835 | 0 | 13 | 0.971 | 2 | 1 |
| 35 | 5.968 | 0 | 12 | 0.935 | 9 | 1 |
| 36 | 5.8 | 0 | 11 | 0.956 | 9 | 1 |
| 37 | 5.777 | 0 | 15 | 0.757 | 9 | 1 |
| 38 | 5.529 | 0 | 11 | 0.973 | 2 | 1 |
| 39 | 5.395 | 2 | 11 | 1 | 5 | 1 |
| 40 | 5.379 | 2 | 10 | 1 | 2 | 1 |
| 41 | 5.371 | 2 | 10 | 1 | 8 | 1 |
| 42 | 4.886 | 2 | 10 | 0.993 | 10 | 1 |
| 43 | 4.637 | 0 | 10 | 0.88 | 3 | 1 |
| 44 | 4.583 | 1 | 9 | 1 | 3 | 1 |
| 45 | 4.483 | 2 | 9 | 1 | 15 | 1 |
| 46 | 4.18 | 0 | 12 | 0.581 | 8 | 1 |
| 47 | 3.585 | 1 | 8 | 1 | 6 | 1 |
| 48 | 3.273 | 0 | 10 | 0.569 | 3 | 1 |
| 49 | 3.076 | 0 | 11 | 0.469 | 9 | 1 |
| 50 | 2.731 | 0 | 7 | 0.779 | 15 | 1 |
| 51 | 2.706 | 0 | 14 | 0.296 | 3 | 1 |
| 52 | 2.693 | 0 | 10 | 0.464 | 9 | 1 |
| 53 | 2.587 | 0 | 9 | 0.563 | 2 | 1 |
| 54 | 2.08 | 0 | 10 | 0.342 | 3 | 1 |
| 55 | 2.054 | 0 | 9 | 0.4 | 7 | 1 |
| 56 | 1.995 | 0 | 7 | 0.549 | 8 | 1 |
| 57 | 1.756 | 0 | 7 | 0.425 | 7 | 1 |
| 58 | 1.614 | 0 | 9 | 0.323 | 9 | 1 |
| 59 | 1.547 | 0 | 9 | 0.255 | 3 | 1 |
| 60 | 1.431 | 0 | 6 | 0.51 | 2 | 1 |
| 61 | 1.421 | 0 | 10 | 0.205 | 4 | 1 |
| 62 | 1.375 | 0 | 13 | 0.155 | 9 | 1 |
| 63 | 0.822 | 0 | 9 | 0.133 | 13 | 1 |
| 64 | 0.786 | 0 | 6 | 0.216 | 15 | 1 |
| 65 | 0.617 | 0 | 8 | 0.092 | 1 | 1 |
| 66 | 0.532 | 0 | 4 | 0.2 | 3 | 1 |
| 67 | 0.523 | 0 | 6 | 0.121 | 3 | 1 |
| 68 | 0.47 | 0 | 6 | 0.1 | 5 | 1 |
| 69 | 0.455 | 0 | 7 | 0.079 | 3 | 1 |
| 70 | 0.396 | 0 | 5 | 0.111 | 5 | 1 |
| 71 | 0.384 | 0 | 5 | 0.111 | 3 | 1 |
| 72 | 0.264 | 0 | 5 | 0.056 | 1 | 1 |
| 73 | 0.264 | 0 | 4 | 0.059 | 3 | 1 |
| 74 | 0.204 | 0 | 4 | 0.039 | 3 | 1 |
| 75 | 0.147 | 0 | 3 | 0.028 | 1 | 1 |
| 76 | 0.145 | 0 | 0 | 0.024 | 1 | 1 |
| 77 | 0.114 | 0 | 2 | 0.032 | 10 | 1 |
| 78 | 0.113 | 0 | 2 | 0.034 | 5 | 1 |
| 79 | 0.093 | 0 | 0 | 0.02 | 2 | 1 |
| 80 | 0.077 | 0 | 0 | 0.017 | 6 | 1 |
| 81 | 0.076 | 0 | 0 | 0.019 | 1 | 1 |
| 82 | 0.076 | 0 | 0 | 0.01 | 1 | 1 |
| 83 | 0.076 | 0 | 0 | 0.015 | 2 | 1 |
| 84 | 0.074 | 0 | 0 | 0.016 | 3 | 1 |
| 85 | 0.072 | 0 | 0 | 0.017 | 2 | 1 |
| 86 | 0.071 | 0 | 0 | 0.013 | 2 | 1 |
| 87 | 0.067 | 0 | 0 | 0.016 | 6 | 1 |
| 88 | 0.058 | 0 | 0 | 0.021 | 1 | 1 |
| 89 | 0.055 | 0 | 0 | 0.014 | 1 | 1 |
| 90 | 0.048 | 0 | 0 | 0.017 | 1 | 1 |
| 91 | 0.04 | 0 | 0 | 0.013 | 1 | 1 |
| 92 | 0.039 | 0 | 0 | 0.007 | 1 | 1 |
| 93 | 0.035 | 0 | 0 | 0.01 | 12 | 1 |
| 94 | 0.033 | 0 | 0 | 0.007 | 1 | 1 |
| 95 | 0.03 | 0 | 0 | 0.008 | 1 | 1 |
| 96 | 0.03 | 0 | 0 | 0.008 | 3 | 1 |
| 97 | 0.029 | 0 | 0 | 0.007 | 5 | 1 |
| 98 | 0.026 | 0 | 0 | 0.005 | 4 | 1 |
| 99 | 0.025 | 0 | 0 | 0.006 | 1 | 1 |
| 100 | 0.024 | 0 | 0 | 0.008 | 4 | 1 |
| 101 | 0.023 | 0 | 0 | 0.005 | 2 | 1 |
| 102 | 0.021 | 0 | 0 | 0.004 | 1 | 1 |
| 103 | 0.019 | 0 | 0 | 0.004 | 5 | 1 |
| 104 | 0.019 | 0 | 0 | 0.007 | 7 | 1 |
| 105 | 0.018 | 0 | 0 | 0.004 | 1 | 1 |
| 106 | 0.017 | 0 | 0 | 0.004 | 14 | 1 |
| 107 | 0.016 | 0 | 0 | 0.002 | 1 | 1 |
| 108 | 0.015 | 0 | 0 | 0.003 | 1 | 1 |
| 109 | 0.014 | 0 | 0 | 0.003 | 3 | 1 |
| 110 | 0.012 | 0 | 0 | 0.003 | 1 | 1 |
| 111 | 0.012 | 0 | 0 | 0.003 | 9 | 1 |
| 112 | 0.011 | 0 | 0 | 0.004 | 2 | 1 |
| 113 | 0.01 | 0 | 0 | 0.002 | 4 | 1 |
| 114 | 0.009 | 0 | 0 | 0.004 | 1 | 1 |
| 115 | 0.009 | 0 | 0 | 0.002 | 10 | 1 |
| 116 | 0.008 | 0 | 0 | 0.003 | 3 | 1 |
| 117 | 0.007 | 0 | 0 | 0.002 | 1 | 1 |
| 118 | 0.007 | 0 | 0 | 0.002 | 10 | 1 |
| 119 | 0.007 | 0 | 0 | 0.001 | 4 | 1 |
| 120 | 0.007 | 0 | 0 | 0.001 | 4 | 1 |
| 121 | 0.006 | 0 | 0 | 0.001 | 1 | 1 |
| 122 | 0.004 | 0 | 0 | 0.001 | 1 | 1 |
| 123 | 0.004 | 0 | 0 | 0.001 | 1 | 1 |
| 124 | 0.004 | 0 | 0 | 0.001 | 3 | 1 |
| 125 | 0.003 | 0 | 0 | 0.001 | 1 | 1 |
| 126 | 0.003 | 0 | 0 | 0.001 | 1 | 1 |
| 127 | 0.003 | 0 | 0 | 0.002 | 1 | 1 |
| 128 | 0.003 | 0 | 0 | 0.001 | 15 | 1 |
| 129 | 0.003 | 0 | 0 | 0.001 | 3 | 1 |
| 130 | 0.003 | 0 | 0 | 0.002 | 3 | 1 |
| 131 | 0.003 | 0 | 0 | 0.001 | 3 | 1 |
| 132 | 0.002 | 0 | 0 | 0.001 | 1 | 1 |
| 133 | 0.002 | 0 | 0 | 0.001 | 1 | 1 |
| 134 | 0.002 | 0 | 0 | 0.001 | 1 | 1 |
| 135 | 0.002 | 0 | 0 | 0.001 | 1 | 1 |
| 136 | 0.002 | 0 | 0 | 0.001 | 4 | 1 |
| 137 | 0.002 | 0 | 0 | 0.001 | 2 | 1 |
| 138 | 0.002 | 0 | 0 | 0.001 | 4 | 1 |
| 139 | 0.001 | 0 | 0 | 0.001 | 1 | 1 |
| 140 | 0.001 | 0 | 0 | 0.001 | 1 | 1 |
| 141 | 0.001 | 0 | 0 | 0.001 | 4 | 1 |
| 142 | 0.001 | 0 | 0 | 0.001 | 6 | 1 |
| 143 | 0.001 | 0 | 0 | 0.001 | 14 | 1 |
| 144 | 0.001 | 0 | 0 | 0.001 | 4 | 1 |
| 145 | 0.001 | 0 | 0 | 0.001 | 3 | 1 |
| 146 | 0 | 0 | 0 | 0 | 1 | 1 |
| 147 | 0 | 0 | 0 | 0 | 1 | 1 |
| 148 | 0 | 0 | 0 | 0 | 1 | 1 |
| 149 | 0 | 0 | 0 | 0 | 1 | 1 |
| 150 | 0 | 0 | 0 | 0 | 1 | 1 |
| 151 | 0 | 0 | 0 | 0 | 1 | 1 |
| 152 | 0 | 0 | 0 | 0 | 1 | 1 |
| 153 | 0 | 0 | 0 | 0 | 1 | 1 |
| 154 | 0 | 0 | 0 | 0 | 1 | 1 |
| 155 | 0 | 0 | 0 | 0 | 1 | 1 |
| 156 | 0 | 0 | 0 | 0 | 1 | 1 |
| 157 | 0 | 0 | 0 | 0 | 1 | 1 |
| 158 | 0 | 0 | 0 | 0 | 1 | 1 |
| 159 | 0 | 0 | 0 | 0 | 1 | 1 |
| 160 | 0 | 0 | 0 | 0 | 1 | 1 |
| 161 | 0 | 0 | 0 | 0 | 1 | 1 |
| 162 | 0 | 0 | 0 | 0 | 1 | 1 |
| 163 | 0 | 0 | 0 | 0 | 1 | 1 |
| 164 | 0 | 0 | 0 | 0 | 1 | 1 |
| 165 | 0 | 0 | 0 | 0 | 1 | 1 |
| 166 | 0 | 0 | 0 | 0 | 1 | 1 |
| 167 | 0 | 0 | 0 | 0 | 1 | 1 |
| 168 | 0 | 0 | 0 | 0 | 1 | 1 |
| 169 | 0 | 0 | 0 | 0 | 1 | 1 |
| 170 | 0 | 0 | 0 | 0 | 1 | 1 |
| 171 | 0 | 0 | 0 | 0 | 1 | 1 |
| 172 | 0 | 0 | 0 | 0 | 1 | 1 |
| 173 | 0 | 0 | 0 | 0 | 1 | 1 |
| 174 | 0 | 0 | 0 | 0 | 1 | 1 |
| 175 | 0 | 0 | 0 | 0 | 1 | 1 |
| 176 | 0 | 0 | 0 | 0 | 1 | 1 |
| 177 | 0 | 0 | 0 | 0 | 1 | 1 |
| 178 | 0 | 0 | 0 | 0 | 1 | 1 |
| 179 | 0 | 0 | 0 | 0 | 1 | 1 |
| 180 | 0 | 0 | 0 | 0 | 1 | 1 |
| 181 | 0 | 0 | 0 | 0 | 1 | 1 |
| 182 | 0 | 0 | 0 | 0 | 1 | 1 |
| 183 | 0 | 0 | 0 | 0 | 1 | 1 |
| 184 | 0 | 0 | 0 | 0 | 1 | 1 |
| 185 | 0 | 0 | 0 | 0 | 1 | 1 |
| 186 | 0 | 0 | 0 | 0 | 1 | 1 |
| 187 | 0 | 0 | 0 | 0 | 1 | 1 |
| 188 | 0 | 0 | 0 | 0 | 1 | 1 |
| 189 | 0 | 0 | 0 | 0 | 1 | 1 |
| 190 | 0 | 0 | 0 | 0 | 1 | 1 |
| 191 | 0 | 0 | 0 | 0 | 1 | 1 |
| 192 | 0 | 0 | 0 | 0 | 1 | 1 |
| 193 | 0 | 0 | 0 | 0 | 1 | 1 |
| 194 | 0 | 0 | 0 | 0 | 1 | 1 |
| 195 | 0 | 0 | 0 | 0 | 1 | 1 |
| 196 | 0 | 0 | 0 | 0 | 1 | 1 |
| 197 | 0 | 0 | 0 | 0 | 1 | 1 |
| 198 | 0 | 0 | 0 | 0 | 1 | 1 |
| 199 | 0 | 0 | 0 | 0 | 1 | 1 |
| 200 | 0 | 0 | 0 | 0 | 1 | 1 |
| 201 | 0 | 0 | 0 | 0 | 1 | 1 |
| 202 | 0 | 0 | 0 | 0 | 1 | 1 |
| 203 | 0 | 0 | 0 | 0 | 1 | 1 |
| 204 | 0 | 0 | 0 | 0 | 1 | 1 |
| 205 | 0 | 0 | 0 | 0 | 1 | 1 |
| 206 | 0 | 0 | 0 | 0 | 1 | 1 |
| 207 | 0 | 0 | 0 | 0 | 1 | 1 |
| 208 | 0 | 0 | 0 | 0 | 1 | 1 |
| 209 | 0 | 0 | 0 | 0 | 1 | 1 |
| 210 | 0 | 0 | 0 | 0 | 1 | 1 |
| 211 | 0 | 0 | 0 | 0 | 1 | 1 |
| 212 | 0 | 0 | 0 | 0 | 1 | 1 |
| 213 | 0 | 0 | 0 | 0 | 1 | 1 |
| 214 | 0 | 0 | 0 | 0 | 1 | 1 |
| 215 | 0 | 0 | 0 | 0 | 1 | 1 |
| 216 | 0 | 0 | 0 | 0 | 1 | 1 |
| 217 | 0 | 0 | 0 | 0 | 1 | 1 |
| 218 | 0 | 0 | 0 | 0 | 1 | 1 |
| 219 | 0 | 0 | 0 | 0 | 1 | 1 |
| 220 | 0 | 0 | 0 | 0 | 1 | 1 |
| 221 | 0 | 0 | 0 | 0 | 1 | 1 |
| 222 | 0 | 0 | 0 | 0 | 1 | 1 |
| 223 | 0 | 0 | 0 | 0 | 1 | 1 |
| 224 | 0 | 0 | 0 | 0 | 1 | 1 |
| 225 | 0 | 0 | 0 | 0 | 1 | 1 |
| 226 | 0 | 0 | 0 | 0 | 1 | 1 |
| 227 | 0 | 0 | 0 | 0 | 1 | 1 |
| 228 | 0 | 0 | 0 | 0 | 1 | 1 |
| 229 | 0 | 0 | 0 | 0 | 1 | 1 |
| 230 | 0 | 0 | 0 | 0 | 1 | 1 |
| 231 | 0 | 0 | 0 | 0 | 1 | 1 |
| 232 | 0 | 0 | 0 | 0 | 1 | 1 |
| 233 | 0 | 0 | 0 | 0 | 1 | 1 |
| 234 | 0 | 0 | 0 | 0 | 1 | 1 |
| 235 | 0 | 0 | 0 | 0 | 1 | 1 |
| 236 | 0 | 0 | 0 | 0 | 1 | 1 |
| 237 | 0 | 0 | 0 | 0 | 1 | 1 |
| 238 | 0 | 0 | 0 | 0 | 1 | 1 |
| 239 | 0 | 0 | 0 | 0 | 1 | 1 |
| 240 | 0 | 0 | 0 | 0 | 1 | 1 |
| 241 | 0 | 0 | 0 | 0 | 1 | 1 |
| 242 | 0 | 0 | 0 | 0 | 1 | 1 |
| 243 | 0 | 0 | 0 | 0 | 1 | 1 |
| 244 | 0 | 0 | 0 | 0 | 1 | 1 |
| 245 | 0 | 0 | 0 | 0 | 1 | 1 |
| 246 | 0 | 0 | 0 | 0 | 1 | 1 |
| 247 | 0 | 0 | 0 | 0 | 1 | 1 |
| 248 | 0 | 0 | 0 | 0 | 1 | 1 |
| 249 | 0 | 0 | 0 | 0 | 1 | 1 |
| 250 | 0 | 0 | 0 | 0 | 1 | 1 |
| 251 | 0 | 0 | 0 | 0 | 1 | 1 |
| 252 | 0 | 0 | 0 | 0 | 1 | 1 |
| 253 | 0 | 0 | 0 | 0 | 1 | 1 |
| 254 | 0 | 0 | 0 | 0 | 1 | 1 |
| 255 | 0 | 0 | 0 | 0 | 1 | 1 |
| 256 | 0 | 0 | 0 | 0 | 1 | 1 |
| 257 | 0 | 0 | 0 | 0 | 1 | 1 |
| 258 | 0 | 0 | 0 | 0 | 1 | 1 |
| 259 | 0 | 0 | 0 | 0 | 1 | 1 |
| 260 | 0 | 0 | 0 | 0 | 1 | 1 |
| 261 | 0 | 0 | 0 | 0 | 1 | 1 |
| 262 | 0 | 0 | 0 | 0 | 1 | 1 |
| 263 | 0 | 0 | 0 | 0 | 1 | 1 |
| 264 | 0 | 0 | 0 | 0 | 1 | 1 |
| 265 | 0 | 0 | 0 | 0 | 1 | 1 |
| 266 | 0 | 0 | 0 | 0 | 1 | 1 |
| 267 | 0 | 0 | 0 | 0 | 1 | 1 |
| 268 | 0 | 0 | 0 | 0 | 1 | 1 |
| 269 | 0 | 0 | 0 | 0 | 1 | 1 |
| 270 | 0 | 0 | 0 | 0 | 1 | 1 |
| 271 | 0 | 0 | 0 | 0 | 1 | 1 |
| 272 | 0 | 0 | 0 | 0 | 1 | 1 |
| 273 | 0 | 0 | 0 | 0 | 1 | 1 |
| 274 | 0 | 0 | 0 | 0 | 1 | 1 |
| 275 | 0 | 0 | 0 | 0 | 1 | 1 |
| 276 | 0 | 0 | 0 | 0 | 1 | 1 |
| 277 | 0 | 0 | 0 | 0 | 1 | 1 |
| 278 | 0 | 0 | 0 | 0 | 1 | 1 |
| 279 | 0 | 0 | 0 | 0 | 1 | 1 |
| 280 | 0 | 0 | 0 | 0 | 1 | 1 |
| 281 | 0 | 0 | 0 | 0 | 1 | 1 |
| 282 | 0 | 0 | 0 | 0 | 1 | 1 |
| 283 | 0 | 0 | 0 | 0 | 1 | 1 |
| 284 | 0 | 0 | 0 | 0 | 1 | 1 |
| 285 | 0 | 0 | 0 | 0 | 1 | 1 |
| 286 | 0 | 0 | 0 | 0 | 1 | 1 |
| 287 | 0 | 0 | 0 | 0 | 1 | 1 |
| 288 | 0 | 0 | 0 | 0 | 1 | 1 |
| 289 | 0 | 0 | 0 | 0 | 1 | 1 |
| 290 | 0 | 0 | 0 | 0 | 1 | 1 |
| 291 | 0 | 0 | 0 | 0 | 1 | 1 |
| 292 | 0 | 0 | 0 | 0 | 1 | 1 |
| 293 | 0 | 0 | 0 | 0 | 1 | 1 |
| 294 | 0 | 0 | 0 | 0 | 1 | 1 |
| 295 | 0 | 0 | 0 | 0 | 1 | 1 |
| 296 | 0 | 0 | 0 | 0 | 1 | 1 |
| 297 | 0 | 0 | 0 | 0 | 1 | 1 |
| 298 | 0 | 0 | 0 | 0 | 1 | 1 |
| 299 | 0 | 0 | 0 | 0 | 1 | 1 |
| 300 | 0 | 0 | 0 | 0 | 1 | 1 |
| 301 | 0 | 0 | 0 | 0 | 1 | 1 |
| 302 | 0 | 0 | 0 | 0 | 1 | 1 |
| 303 | 0 | 0 | 0 | 0 | 1 | 1 |
| 304 | 0 | 0 | 0 | 0 | 1 | 1 |
| 305 | 0 | 0 | 0 | 0 | 1 | 1 |
| 306 | 0 | 0 | 0 | 0 | 1 | 1 |
| 307 | 0 | 0 | 0 | 0 | 1 | 1 |
| 308 | 0 | 0 | 0 | 0 | 1 | 1 |
| 309 | 0 | 0 | 0 | 0 | 1 | 1 |
| 310 | 0 | 0 | 0 | 0 | 1 | 1 |
| 311 | 0 | 0 | 0 | 0 | 1 | 1 |
| 312 | 0 | 0 | 0 | 0 | 1 | 1 |
| 313 | 0 | 0 | 0 | 0 | 1 | 1 |
| 314 | 0 | 0 | 0 | 0 | 1 | 1 |
| 315 | 0 | 0 | 0 | 0 | 1 | 1 |
| 316 | 0 | 0 | 0 | 0 | 1 | 1 |
| 317 | 0 | 0 | 0 | 0 | 1 | 1 |
| 318 | 0 | 0 | 0 | 0 | 1 | 1 |
| 319 | 0 | 0 | 0 | 0 | 1 | 1 |
| 320 | 0 | 0 | 0 | 0 | 1 | 1 |
| 321 | 0 | 0 | 0 | 0 | 1 | 1 |
| 322 | 0 | 0 | 0 | 0 | 1 | 1 |
| 323 | 0 | 0 | 0 | 0 | 1 | 1 |
| 324 | 0 | 0 | 0 | 0 | 1 | 1 |
| 325 | 0 | 0 | 0 | 0 | 1 | 1 |
| 326 | 0 | 0 | 0 | 0 | 1 | 1 |
| 327 | 0 | 0 | 0 | 0 | 1 | 1 |
| 328 | 0 | 0 | 0 | 0 | 1 | 1 |
| 329 | 0 | 0 | 0 | 0 | 1 | 1 |
| 330 | 0 | 0 | 0 | 0 | 1 | 1 |
| 331 | 0 | 0 | 0 | 0 | 1 | 1 |
| 332 | 0 | 0 | 0 | 0 | 1 | 1 |
| 333 | 0 | 0 | 0 | 0 | 1 | 1 |
| 334 | 0 | 0 | 0 | 0 | 1 | 1 |
| 335 | 0 | 0 | 0 | 0 | 1 | 1 |
| 336 | 0 | 0 | 0 | 0 | 7 | 1 |
| 337 | 0 | 0 | 0 | 0 | 1 | 1 |
| 338 | 0 | 0 | 0 | 0 | 1 | 1 |
| 339 | 0 | 0 | 0 | 0 | 1 | 1 |
| 340 | 0 | 0 | 0 | 0 | 1 | 1 |
| 341 | 0 | 0 | 0 | 0 | 1 | 1 |
| 342 | 0 | 0 | 0 | 0 | 1 | 1 |
| 343 | 0 | 0 | 0 | 0 | 1 | 1 |
| 344 | 0 | 0 | 0 | 0 | 1 | 1 |
| 345 | 0 | 0 | 0 | 0 | 1 | 1 |
| 346 | 0 | 0 | 0 | 0 | 1 | 1 |
| 347 | 0 | 0 | 0 | 0 | 1 | 1 |
| 348 | 0 | 0 | 0 | 0 | 1 | 1 |
| 349 | 0 | 0 | 0 | 0 | 1 | 1 |
| 350 | 0 | 0 | 0 | 0 | 1 | 1 |
| 351 | 0 | 0 | 0 | 0 | 1 | 1 |
| 352 | 0 | 0 | 0 | 0 | 1 | 1 |
| 353 | 0 | 0 | 0 | 0 | 1 | 1 |
| 354 | 0 | 0 | 0 | 0 | 1 | 1 |
| 355 | 0 | 0 | 0 | 0 | 1 | 1 |
| 356 | 0 | 0 | 0 | 0 | 1 | 1 |
| 357 | 0 | 0 | 0 | 0 | 1 | 1 |
| 358 | 0 | 0 | 0 | 0 | 9 | 1 |
| 359 | 0 | 0 | 0 | 0 | 3 | 1 |
| 360 | 0 | 0 | 0 | 0 | 9 | 1 |
| 361 | 0 | 0 | 0 | 0 | 3 | 1 |
| 362 | 0 | 0 | 0 | 0 | 10 | 1 |
| 363 | 0 | 0 | 0 | 0 | 3 | 1 |
| 364 | 0 | 0 | 0 | 0 | 4 | 1 |
| 365 | 0 | 0 | 0 | 0 | 9 | 1 |
| 366 | 0 | 0 | 0 | 0 | 9 | 1 |
| 367 | 0 | 0 | 0 | 0 | 4 | 1 |
| 368 | 0 | 0 | 0 | 0 | 3 | 1 |
| 369 | 0 | 0 | 0 | 0 | 2 | 1 |
| 370 | 0 | 0 | 0 | 0 | 3 | 1 |
| 371 | 0 | 0 | 0 | 0 | 3 | 1 |
| 372 | 0 | 0 | 0 | 0 | 12 | 1 |
| 373 | 0 | 0 | 0 | 0 | 2 | 1 |
| 374 | 0 | 0 | 0 | 0 | 9 | 1 |
| 375 | 0 | 0 | 0 | 0 | 3 | 1 |
| 376 | 0 | 0 | 0 | 0 | 6 | 1 |
| 377 | 0 | 0 | 0 | 0 | 10 | 1 |
| 378 | 0 | 0 | 0 | 0 | 8 | 1 |
| 379 | 0 | 0 | 0 | 0 | 2 | 1 |
| 380 | 0 | 0 | 0 | 0 | 10 | 1 |
| 381 | 0 | 0 | 0 | 0 | 3 | 1 |
| 382 | 0 | 0 | 0 | 0 | 9 | 1 |
| 383 | 0 | 0 | 0 | 0 | 2 | 1 |
| 384 | 0 | 0 | 0 | 0 | 2 | 1 |
| 385 | 0 | 0 | 0 | 0 | 3 | 1 |
| 386 | 0 | 0 | 0 | 0 | 9 | 1 |
| 387 | 0 | 0 | 0 | 0 | 3 | 1 |
| 388 | 0 | 0 | 0 | 0 | 2 | 1 |
| 389 | 0 | 0 | 0 | 0 | 4 | 1 |
| 390 | 0 | 0 | 0 | 0 | 2 | 1 |
| 391 | 0 | 0 | 0 | 0 | 3 | 1 |
| 392 | 0 | 0 | 0 | 0 | 10 | 1 |
| 393 | 0 | 0 | 0 | 0 | 3 | 1 |
| 394 | 0 | 0 | 0 | 0 | 13 | 1 |
| 395 | 0 | 0 | 0 | 0 | 6 | 1 |
| 396 | 0 | 0 | 0 | 0 | 2 | 1 |
| 397 | 0 | 0 | 0 | 0 | 3 | 1 |
| 398 | 0 | 0 | 0 | 0 | 10 | 1 |
| 399 | 0 | 0 | 0 | 0 | 6 | 1 |
| 400 | 0 | 0 | 0 | 0 | 2 | 1 |
| 401 | 0 | 0 | 0 | 0 | 10 | 1 |
| 402 | 0 | 0 | 0 | 0 | 2 | 1 |
| 403 | 0 | 0 | 0 | 0 | 6 | 1 |
| 404 | 0 | 0 | 0 | 0 | 4 | 1 |
| 405 | 0 | 0 | 0 | 0 | 3 | 1 |
| 406 | 0 | 0 | 0 | 0 | 5 | 1 |
| 407 | 0 | 0 | 0 | 0 | 3 | 1 |
| 408 | 0 | 0 | 0 | 0 | 10 | 1 |
| 409 | 0 | 0 | 0 | 0 | 9 | 1 |
| 410 | 0 | 0 | 0 | 0 | 9 | 1 |
| 411 | 0 | 0 | 0 | 0 | 3 | 1 |
| 412 | 0 | 0 | 0 | 0 | 9 | 1 |
| 413 | 0 | 0 | 0 | 0 | 7 | 1 |
| 414 | 0 | 0 | 0 | 0 | 2 | 1 |
| 415 | 0 | 0 | 0 | 0 | 2 | 1 |
| 416 | 0 | 0 | 0 | 0 | 4 | 1 |
| 417 | 0 | 0 | 0 | 0 | 10 | 1 |
| 418 | 0 | 0 | 0 | 0 | 3 | 1 |
| 419 | 0 | 0 | 0 | 0 | 2 | 1 |
| 420 | 0 | 0 | 0 | 0 | 2 | 1 |
| 421 | 0 | 0 | 0 | 0 | 3 | 1 |
| 422 | 0 | 0 | 0 | 0 | 3 | 1 |
| 423 | 0 | 0 | 0 | 0 | 9 | 1 |
| 424 | 0 | 0 | 0 | 0 | 12 | 1 |
| 425 | 0 | 0 | 0 | 0 | 2 | 1 |
| 426 | 0 | 0 | 0 | 0 | 9 | 1 |
| 427 | 0 | 0 | 0 | 0 | 3 | 1 |
| 428 | 0 | 0 | 0 | 0 | 2 | 1 |
| 429 | 0 | 0 | 0 | 0 | 3 | 1 |
| 430 | 0 | 0 | 0 | 0 | 10 | 1 |
| 431 | 0 | 0 | 0 | 0 | 2 | 1 |
| 432 | 0 | 0 | 0 | 0 | 10 | 1 |
| 433 | 0 | 0 | 0 | 0 | 14 | 1 |
| 434 | 0 | 0 | 0 | 0 | 14 | 1 |
| 435 | 0 | 0 | 0 | 0 | 6 | 1 |
| 436 | 0 | 0 | 0 | 0 | 3 | 1 |
| 437 | 0 | 0 | 0 | 0 | 2 | 1 |
| 438 | 0 | 0 | 0 | 0 | 10 | 1 |
| 439 | 0 | 0 | 0 | 0 | 3 | 1 |
| 440 | 0 | 0 | 0 | 0 | 14 | 1 |
| 441 | 0 | 0 | 0 | 0 | 10 | 1 |
| 442 | 0 | 0 | 0 | 0 | 4 | 1 |
| 443 | 0 | 0 | 0 | 0 | 9 | 1 |
| 444 | 0 | 0 | 0 | 0 | 2 | 1 |
| 445 | 0 | 0 | 0 | 0 | 4 | 1 |
| 446 | 0 | 0 | 0 | 0 | 3 | 1 |
| 447 | 0 | 0 | 0 | 0 | 5 | 1 |
| 448 | 0 | 0 | 0 | 0 | 6 | 1 |
| 449 | 0 | 0 | 0 | 0 | 3 | 1 |
| 450 | 0 | 0 | 0 | 0 | 10 | 1 |
| 451 | 0 | 0 | 0 | 0 | 2 | 1 |
| 452 | 0 | 0 | 0 | 0 | 2 | 1 |
| 453 | 0 | 0 | 0 | 0 | 9 | 1 |
| 454 | 0 | 0 | 0 | 0 | 7 | 1 |
| 455 | 0 | 0 | 0 | 0 | 3 | 1 |
| 456 | 0 | 0 | 0 | 0 | 9 | 1 |
| 457 | 0 | 0 | 0 | 0 | 6 | 1 |
| 458 | 0 | 0 | 0 | 0 | 6 | 1 |
| 459 | 0 | 0 | 0 | 0 | 6 | 1 |
| 460 | 0 | 0 | 0 | 0 | 5 | 1 |
| 461 | 0 | 0 | 0 | 0 | 10 | 1 |
| 462 | 0 | 0 | 0 | 0 | 2 | 1 |
| 463 | 0 | 0 | 0 | 0 | 3 | 1 |
| 464 | 0 | 0 | 0 | 0 | 7 | 1 |
| 465 | 0 | 0 | 0 | 0 | 6 | 1 |
| 466 | 0 | 0 | 0 | 0 | 5 | 1 |
| 467 | 0 | 0 | 0 | 0 | 8 | 1 |
| 468 | 0 | 0 | 0 | 0 | 9 | 1 |
| 469 | 0 | 0 | 0 | 0 | 3 | 1 |
| 470 | 0 | 0 | 0 | 0 | 9 | 1 |
| 471 | 0 | 0 | 0 | 0 | 6 | 1 |
| 472 | 0 | 0 | 0 | 0 | 2 | 1 |
| 473 | 0 | 0 | 0 | 0 | 4 | 1 |
| 474 | 0 | 0 | 0 | 0 | 13 | 1 |
| 475 | 0 | 0 | 0 | 0 | 3 | 1 |
| 476 | 0 | 0 | 0 | 0 | 3 | 1 |
| 477 | 0 | 0 | 0 | 0 | 10 | 1 |
| 478 | 0 | 0 | 0 | 0 | 9 | 1 |
| 479 | 0 | 0 | 0 | 0 | 14 | 1 |
| 480 | 0 | 0 | 0 | 0 | 13 | 1 |
| 481 | 0 | 0 | 0 | 0 | 4 | 1 |
| 482 | 0 | 0 | 0 | 0 | 3 | 1 |
| 483 | 0 | 0 | 0 | 0 | 13 | 1 |
| 484 | 0 | 0 | 0 | 0 | 10 | 1 |
| 485 | 0 | 0 | 0 | 0 | 4 | 1 |
| 486 | 0 | 0 | 0 | 0 | 10 | 1 |
| 487 | 0 | 0 | 0 | 0 | 3 | 1 |
| 488 | 0 | 0 | 0 | 0 | 9 | 1 |
| 489 | 0 | 0 | 0 | 0 | 4 | 1 |
| 490 | 0 | 0 | 0 | 0 | 2 | 1 |
| 491 | 0 | 0 | 0 | 0 | 2 | 1 |
| 492 | 0 | 0 | 0 | 0 | 2 | 1 |
| 493 | 0 | 0 | 0 | 0 | 2 | 1 |
| 494 | 0 | 0 | 0 | 0 | 3 | 1 |
| 495 | 0 | 0 | 0 | 0 | 3 | 1 |
| 496 | 0 | 0 | 0 | 0 | 2 | 1 |
| 497 | 0 | 0 | 0 | 0 | 12 | 1 |
| 498 | 0 | 0 | 0 | 0 | 3 | 1 |
| 499 | 0 | 0 | 0 | 0 | 5 | 1 |
| 500 | 0 | 0 | 0 | 0 | 7 | 1 |
| 501 | 0 | 0 | 0 | 0 | 5 | 1 |
| 502 | 0 | 0 | 0 | 0 | 3 | 1 |
| 503 | 0 | 0 | 0 | 0 | 2 | 1 |
| 504 | 0 | 0 | 0 | 0 | 4 | 1 |
| 505 | 0 | 0 | 0 | 0 | 3 | 1 |
| 506 | 0 | 0 | 0 | 0 | 10 | 1 |
| 507 | 0 | 0 | 0 | 0 | 3 | 1 |
| 508 | 0 | 0 | 0 | 0 | 2 | 1 |
| 509 | 0 | 0 | 0 | 0 | 10 | 1 |
| 510 | 0 | 0 | 0 | 0 | 2 | 1 |
| 511 | 0 | 0 | 0 | 0 | 6 | 1 |
| 512 | 0 | 0 | 0 | 0 | 4 | 1 |
| 513 | 0 | 0 | 0 | 0 | 2 | 1 |
| 514 | 0 | 0 | 0 | 0 | 12 | 1 |
| 515 | 0 | 0 | 0 | 0 | 2 | 1 |
| 516 | 0 | 0 | 0 | 0 | 2 | 1 |
| 517 | 0 | 0 | 0 | 0 | 6 | 1 |
| 518 | 0 | 0 | 0 | 0 | 3 | 1 |
| 519 | 0 | 0 | 0 | 0 | 9 | 1 |
| 520 | 0 | 0 | 0 | 0 | 14 | 1 |
| 521 | 0 | 0 | 0 | 0 | 3 | 1 |
| 522 | 0 | 0 | 0 | 0 | 10 | 1 |
| 523 | 0 | 0 | 0 | 0 | 4 | 1 |
| 524 | 0 | 0 | 0 | 0 | 10 | 1 |
| 525 | 0 | 0 | 0 | 0 | 2 | 1 |
| 526 | 0 | 0 | 0 | 0 | 2 | 1 |
| 527 | 0 | 0 | 0 | 0 | 3 | 1 |
| 528 | 0 | 0 | 0 | 0 | 5 | 1 |
| 529 | 0 | 0 | 0 | 0 | 4 | 1 |
| 530 | 0 | 0 | 0 | 0 | 15 | 1 |
| 531 | 0 | 0 | 0 | 0 | 12 | 1 |
| 532 | 0 | 0 | 0 | 0 | 9 | 1 |
| 533 | 0 | 0 | 0 | 0 | 10 | 1 |
| 534 | 0 | 0 | 0 | 0 | 2 | 1 |
| 535 | 0 | 0 | 0 | 0 | 2 | 1 |
| 536 | 0 | 0 | 0 | 0 | 5 | 1 |
| 537 | 0 | 0 | 0 | 0 | 6 | 1 |
| 538 | 0 | 0 | 0 | 0 | 3 | 1 |
| 539 | 0 | 0 | 0 | 0 | 5 | 1 |
| 540 | 0 | 0 | 0 | 0 | 4 | 1 |
| 541 | 0 | 0 | 0 | 0 | 5 | 1 |
| 542 | 0 | 0 | 0 | 0 | 10 | 1 |
| 543 | 0 | 0 | 0 | 0 | 2 | 1 |
| 544 | 0 | 0 | 0 | 0 | 2 | 1 |
| 545 | 0 | 0 | 0 | 0 | 2 | 1 |
| 546 | 0 | 0 | 0 | 0 | 4 | 1 |
| 547 | 0 | 0 | 0 | 0 | 3 | 1 |
| 548 | 0 | 0 | 0 | 0 | 2 | 1 |
| 549 | 0 | 0 | 0 | 0 | 2 | 1 |
| 550 | 0 | 0 | 0 | 0 | 9 | 1 |
| 551 | 0 | 0 | 0 | 0 | 3 | 1 |
| 552 | 0 | 0 | 0 | 0 | 15 | 1 |
| 553 | 0 | 0 | 0 | 0 | 3 | 1 |
| 554 | 0 | 0 | 0 | 0 | 3 | 1 |
| 555 | 0 | 0 | 0 | 0 | 3 | 1 |
| 556 | 0 | 0 | 0 | 0 | 9 | 1 |
| 557 | 0 | 0 | 0 | 0 | 3 | 1 |
| 558 | 0 | 0 | 0 | 0 | 3 | 1 |
| 559 | 0 | 0 | 0 | 0 | 10 | 1 |
| 560 | 0 | 0 | 0 | 0 | 10 | 1 |
| 561 | 0 | 0 | 0 | 0 | 4 | 1 |
| 562 | 0 | 0 | 0 | 0 | 3 | 1 |
| 563 | 0 | 0 | 0 | 0 | 8 | 1 |
| 564 | 0 | 0 | 0 | 0 | 9 | 1 |
| 565 | 0 | 0 | 0 | 0 | 8 | 1 |
| 566 | 0 | 0 | 0 | 0 | 9 | 1 |
| 567 | 0 | 0 | 0 | 0 | 4 | 1 |
| 568 | 0 | 0 | 0 | 0 | 4 | 1 |
| 569 | 0 | 0 | 0 | 0 | 6 | 1 |
| 570 | 0 | 0 | 0 | 0 | 5 | 1 |
| 571 | 0 | 0 | 0 | 0 | 9 | 1 |
| 572 | 0 | 0 | 0 | 0 | 9 | 1 |
| 573 | 0 | 0 | 0 | 0 | 5 | 1 |
| 574 | 0 | 0 | 0 | 0 | 8 | 1 |
| 575 | 0 | 0 | 0 | 0 | 3 | 1 |
| 576 | 0 | 0 | 0 | 0 | 5 | 1 |
| 577 | 0 | 0 | 0 | 0 | 3 | 1 |
| 578 | 0 | 0 | 0 | 0 | 6 | 1 |
| 579 | 0 | 0 | 0 | 0 | 2 | 1 |
| 580 | 0 | 0 | 0 | 0 | 3 | 1 |
| 581 | 0 | 0 | 0 | 0 | 5 | 1 |
| 582 | 0 | 0 | 0 | 0 | 10 | 1 |
| 583 | 0 | 0 | 0 | 0 | 3 | 1 |
| 584 | 0 | 0 | 0 | 0 | 3 | 1 |
| 585 | 0 | 0 | 0 | 0 | 3 | 1 |
| 586 | 0 | 0 | 0 | 0 | 4 | 1 |
| 587 | 0 | 0 | 0 | 0 | 2 | 1 |
| 588 | 0 | 0 | 0 | 0 | 9 | 1 |
| 589 | 0 | 0 | 0 | 0 | 2 | 1 |
| 590 | 0 | 0 | 0 | 0 | 9 | 1 |
| 591 | 0 | 0 | 0 | 0 | 10 | 1 |
| 592 | 0 | 0 | 0 | 0 | 9 | 1 |
| 593 | 0 | 0 | 0 | 0 | 14 | 1 |
| 594 | 0 | 0 | 0 | 0 | 3 | 1 |
| 595 | 0 | 0 | 0 | 0 | 9 | 1 |
| 596 | 0 | 0 | 0 | 0 | 10 | 1 |
| 597 | 0 | 0 | 0 | 0 | 4 | 1 |
| 598 | 0 | 0 | 0 | 0 | 3 | 1 |
| 599 | 0 | 0 | 0 | 0 | 3 | 1 |
| 600 | 0 | 0 | 0 | 0 | 2 | 1 |
| 601 | 0 | 0 | 0 | 0 | 7 | 1 |
| 602 | 0 | 0 | 0 | 0 | 10 | 1 |
| 603 | 0 | 0 | 0 | 0 | 5 | 1 |
| 604 | 0 | 0 | 0 | 0 | 4 | 1 |
| 605 | 0 | 0 | 0 | 0 | 2 | 1 |
| 606 | 0 | 0 | 0 | 0 | 6 | 1 |
| 607 | 0 | 0 | 0 | 0 | 10 | 1 |
| 608 | 0 | 0 | 0 | 0 | 2 | 1 |
| 609 | 0 | 0 | 0 | 0 | 5 | 1 |
| 610 | 0 | 0 | 0 | 0 | 9 | 1 |
| 611 | 0 | 0 | 0 | 0 | 6 | 1 |
| 612 | 0 | 0 | 0 | 0 | 13 | 1 |
| 613 | 0 | 0 | 0 | 0 | 3 | 1 |
| 614 | 0 | 0 | 0 | 0 | 3 | 1 |
| 615 | 0 | 0 | 0 | 0 | 10 | 1 |
| 616 | 0 | 0 | 0 | 0 | 10 | 1 |
| 617 | 0 | 0 | 0 | 0 | 2 | 1 |
| 618 | 0 | 0 | 0 | 0 | 7 | 1 |
| 619 | 0 | 0 | 0 | 0 | 7 | 1 |
| 620 | 0 | 0 | 0 | 0 | 3 | 1 |
| 621 | 0 | 0 | 0 | 0 | 6 | 1 |
| 622 | 0 | 0 | 0 | 0 | 3 | 1 |
| 623 | 0 | 0 | 0 | 0 | 10 | 1 |
| 624 | 0 | 0 | 0 | 0 | 7 | 1 |
| 625 | 0 | 0 | 0 | 0 | 14 | 1 |
| 626 | 0 | 0 | 0 | 0 | 2 | 1 |
| 627 | 0 | 0 | 0 | 0 | 2 | 1 |
| 628 | 0 | 0 | 0 | 0 | 9 | 1 |
| 629 | 0 | 0 | 0 | 0 | 9 | 1 |
| 630 | 0 | 0 | 0 | 0 | 9 | 1 |
| 631 | 0 | 0 | 0 | 0 | 10 | 1 |
| 632 | 0 | 0 | 0 | 0 | 2 | 1 |
| 633 | 0 | 0 | 0 | 0 | 4 | 1 |
| 634 | 0 | 0 | 0 | 0 | 3 | 1 |
| 635 | 0 | 0 | 0 | 0 | 3 | 1 |
| 636 | 0 | 0 | 0 | 0 | 14 | 1 |
| 637 | 0 | 0 | 0 | 0 | 2 | 1 |
| 638 | 0 | 0 | 0 | 0 | 4 | 1 |
| 639 | 0 | 0 | 0 | 0 | 2 | 1 |
| 640 | 0 | 0 | 0 | 0 | 3 | 1 |
| 641 | 0 | 0 | 0 | 0 | 2 | 1 |
| 642 | 0 | 0 | 0 | 0 | 3 | 1 |
| 643 | 0 | 0 | 0 | 0 | 14 | 1 |
| 644 | 0 | 0 | 0 | 0 | 4 | 1 |
| 645 | 0 | 0 | 0 | 0 | 9 | 1 |
| 646 | 0 | 0 | 0 | 0 | 3 | 1 |
| 647 | 0 | 0 | 0 | 0 | 9 | 1 |
| 648 | 0 | 0 | 0 | 0 | 13 | 1 |
| 649 | 0 | 0 | 0 | 0 | 3 | 1 |
| 650 | 0 | 0 | 0 | 0 | 13 | 1 |
| 651 | 0 | 0 | 0 | 0 | 3 | 1 |
| 652 | 0 | 0 | 0 | 0 | 10 | 1 |
| 653 | 0 | 0 | 0 | 0 | 3 | 1 |
| 654 | 0 | 0 | 0 | 0 | 2 | 1 |
| 655 | 0 | 0 | 0 | 0 | 9 | 1 |
| 656 | 0 | 0 | 0 | 0 | 3 | 1 |
| 657 | 0 | 0 | 0 | 0 | 2 | 1 |
| 658 | 0 | 0 | 0 | 0 | 3 | 1 |
| 659 | 0 | 0 | 0 | 0 | 3 | 1 |
| 660 | 0 | 0 | 0 | 0 | 4 | 1 |
| 661 | 0 | 0 | 0 | 0 | 9 | 1 |
| 662 | 0 | 0 | 0 | 0 | 3 | 1 |
| 663 | 0 | 0 | 0 | 0 | 15 | 1 |
| 664 | 0 | 0 | 0 | 0 | 4 | 1 |
| 665 | 0 | 0 | 0 | 0 | 3 | 1 |
| 666 | 0 | 0 | 0 | 0 | 5 | 1 |
| 667 | 0 | 0 | 0 | 0 | 2 | 1 |
| 668 | 0 | 0 | 0 | 0 | 10 | 1 |
| 669 | 0 | 0 | 0 | 0 | 9 | 1 |
| 670 | 0 | 0 | 0 | 0 | 3 | 1 |
| 671 | 0 | 0 | 0 | 0 | 3 | 1 |
| 672 | 0 | 0 | 0 | 0 | 4 | 1 |
| 673 | 0 | 0 | 0 | 0 | 2 | 1 |
| 674 | 0 | 0 | 0 | 0 | 3 | 1 |
| 675 | 0 | 0 | 0 | 0 | 7 | 1 |
| 676 | 0 | 0 | 0 | 0 | 2 | 1 |
| 677 | 0 | 0 | 0 | 0 | 3 | 1 |
| 678 | 0 | 0 | 0 | 0 | 10 | 1 |
| 679 | 0 | 0 | 0 | 0 | 3 | 1 |
| 680 | 0 | 0 | 0 | 0 | 13 | 1 |
| 681 | 0 | 0 | 0 | 0 | 9 | 1 |
| 682 | 0 | 0 | 0 | 0 | 2 | 1 |
| 683 | 0 | 0 | 0 | 0 | 14 | 1 |
| 684 | 0 | 0 | 0 | 0 | 4 | 1 |
| 685 | 0 | 0 | 0 | 0 | 2 | 1 |
| 686 | 0 | 0 | 0 | 0 | 2 | 1 |
| 687 | 0 | 0 | 0 | 0 | 3 | 1 |
| 688 | 0 | 0 | 0 | 0 | 10 | 1 |
| 689 | 0 | 0 | 0 | 0 | 2 | 1 |
| 690 | 0 | 0 | 0 | 0 | 4 | 1 |
| 691 | 0 | 0 | 0 | 0 | 10 | 1 |
| 692 | 0 | 0 | 0 | 0 | 4 | 1 |
| 693 | 0 | 0 | 0 | 0 | 7 | 1 |
| 694 | 0 | 0 | 0 | 0 | 4 | 1 |
| 695 | 0 | 0 | 0 | 0 | 7 | 1 |
| 696 | 0 | 0 | 0 | 0 | 9 | 1 |
| 697 | 0 | 0 | 0 | 0 | 6 | 1 |
| 698 | 0 | 0 | 0 | 0 | 5 | 1 |
| 699 | 0 | 0 | 0 | 0 | 10 | 1 |
| 700 | 0 | 0 | 0 | 0 | 10 | 1 |
| 701 | 0 | 0 | 0 | 0 | 9 | 1 |
| 702 | 0 | 0 | 0 | 0 | 2 | 1 |
| 703 | 0 | 0 | 0 | 0 | 9 | 1 |
| 704 | 0 | 0 | 0 | 0 | 6 | 1 |
| 705 | 0 | 0 | 0 | 0 | 9 | 1 |
| 706 | 0 | 0 | 0 | 0 | 2 | 1 |
| 707 | 0 | 0 | 0 | 0 | 10 | 1 |
| 708 | 0 | 0 | 0 | 0 | 4 | 1 |
| 709 | 0 | 0 | 0 | 0 | 4 | 1 |
| 710 | 0 | 0 | 0 | 0 | 4 | 1 |
| 711 | 0 | 0 | 0 | 0 | 2 | 1 |
| 712 | 0 | 0 | 0 | 0 | 10 | 1 |
| 713 | 0 | 0 | 0 | 0 | 6 | 1 |
| 714 | 0 | 0 | 0 | 0 | 5 | 1 |
| 715 | 0 | 0 | 0 | 0 | 2 | 1 |
| 716 | 0 | 0 | 0 | 0 | 2 | 1 |
| 717 | 0 | 0 | 0 | 0 | 13 | 1 |
| 718 | 0 | 0 | 0 | 0 | 5 | 1 |
| 719 | 0 | 0 | 0 | 0 | 9 | 1 |
| 720 | 0 | 0 | 0 | 0 | 9 | 1 |
| 721 | 0 | 0 | 0 | 0 | 9 | 1 |
| 722 | 0 | 0 | 0 | 0 | 6 | 1 |
| 723 | 0 | 0 | 0 | 0 | 11 | 1 |
| 724 | 0 | 0 | 0 | 0 | 7 | 1 |
| 725 | 0 | 0 | 0 | 0 | 4 | 1 |
| 726 | 0 | 0 | 0 | 0 | 2 | 1 |
| 727 | 0 | 0 | 0 | 0 | 3 | 1 |
| 728 | 0 | 0 | 0 | 0 | 2 | 1 |
| 729 | 0 | 0 | 0 | 0 | 3 | 1 |
| 730 | 0 | 0 | 0 | 0 | 12 | 1 |
| 731 | 0 | 0 | 0 | 0 | 5 | 1 |
| 732 | 0 | 0 | 0 | 0 | 14 | 1 |
| 733 | 0 | 0 | 0 | 0 | 9 | 1 |
| 734 | 0 | 0 | 0 | 0 | 4 | 1 |
| 735 | 0 | 0 | 0 | 0 | 10 | 1 |
| 736 | 0 | 0 | 0 | 0 | 2 | 1 |
| 737 | 0 | 0 | 0 | 0 | 3 | 1 |
| 738 | 0 | 0 | 0 | 0 | 14 | 1 |
| 739 | 0 | 0 | 0 | 0 | 7 | 1 |
| 740 | 0 | 0 | 0 | 0 | 3 | 1 |
| 741 | 0 | 0 | 0 | 0 | 10 | 1 |
| 742 | 0 | 0 | 0 | 0 | 14 | 1 |
| 743 | 0 | 0 | 0 | 0 | 4 | 1 |
| 744 | 0 | 0 | 0 | 0 | 2 | 1 |
| 745 | 0 | 0 | 0 | 0 | 5 | 1 |
| 746 | 0 | 0 | 0 | 0 | 3 | 1 |
| 747 | 0 | 0 | 0 | 0 | 14 | 1 |
| 748 | 0 | 0 | 0 | 0 | 3 | 1 |
| 749 | 0 | 0 | 0 | 0 | 3 | 1 |
| 750 | 0 | 0 | 0 | 0 | 10 | 1 |
| 751 | 0 | 0 | 0 | 0 | 14 | 1 |
| 752 | 0 | 0 | 0 | 0 | 12 | 1 |
| 753 | 0 | 0 | 0 | 0 | 10 | 1 |
| 754 | 0 | 0 | 0 | 0 | 3 | 1 |
| 755 | 0 | 0 | 0 | 0 | 2 | 1 |
| 756 | 0 | 0 | 0 | 0 | 3 | 1 |
| 757 | 0 | 0 | 0 | 0 | 2 | 1 |
| 758 | 0 | 0 | 0 | 0 | 9 | 1 |
| 759 | 0 | 0 | 0 | 0 | 3 | 1 |
| 760 | 0 | 0 | 0 | 0 | 6 | 1 |
| 761 | 0 | 0 | 0 | 0 | 2 | 1 |
| 762 | 0 | 0 | 0 | 0 | 9 | 1 |
| 763 | 0 | 0 | 0 | 0 | 5 | 1 |
| 764 | 0 | 0 | 0 | 0 | 15 | 1 |
| 765 | 0 | 0 | 0 | 0 | 4 | 1 |
| 766 | 0 | 0 | 0 | 0 | 9 | 1 |
| 767 | 0 | 0 | 0 | 0 | 9 | 1 |
| 768 | 0 | 0 | 0 | 0 | 2 | 1 |
| 769 | 0 | 0 | 0 | 0 | 4 | 1 |
| 770 | 0 | 0 | 0 | 0 | 9 | 1 |
| 771 | 0 | 0 | 0 | 0 | 3 | 1 |
| 772 | 0 | 0 | 0 | 0 | 9 | 1 |
| 773 | 0 | 0 | 0 | 0 | 7 | 1 |
| 774 | 0 | 0 | 0 | 0 | 3 | 1 |
| 775 | 0 | 0 | 0 | 0 | 10 | 1 |
| 776 | 0 | 0 | 0 | 0 | 2 | 1 |
| 777 | 0 | 0 | 0 | 0 | 5 | 1 |
| 778 | 0 | 0 | 0 | 0 | 4 | 1 |
| 779 | 0 | 0 | 0 | 0 | 9 | 1 |
| 780 | 0 | 0 | 0 | 0 | 9 | 1 |
| 781 | 0 | 0 | 0 | 0 | 14 | 1 |
| 782 | 0 | 0 | 0 | 0 | 10 | 1 |
| 783 | 0 | 0 | 0 | 0 | 2 | 1 |
| 784 | 0 | 0 | 0 | 0 | 3 | 1 |
| 785 | 0 | 0 | 0 | 0 | 2 | 1 |
| 786 | 0 | 0 | 0 | 0 | 2 | 1 |
| 787 | 0 | 0 | 0 | 0 | 12 | 1 |
| 788 | 0 | 0 | 0 | 0 | 3 | 1 |
| 789 | 0 | 0 | 0 | 0 | 3 | 1 |
| 790 | 0 | 0 | 0 | 0 | 4 | 1 |
| 791 | 0 | 0 | 0 | 0 | 14 | 1 |
| 792 | 0 | 0 | 0 | 0 | 2 | 1 |
| 793 | 0 | 0 | 0 | 0 | 9 | 1 |
| 794 | 0 | 0 | 0 | 0 | 9 | 1 |
| 795 | 0 | 0 | 0 | 0 | 2 | 1 |
| 796 | 0 | 0 | 0 | 0 | 3 | 1 |
| 797 | 0 | 0 | 0 | 0 | 2 | 1 |
| 798 | 0 | 0 | 0 | 0 | 10 | 1 |
| 799 | 0 | 0 | 0 | 0 | 14 | 1 |
| 800 | 0 | 0 | 0 | 0 | 14 | 1 |
| 801 | 0 | 0 | 0 | 0 | 7 | 1 |
| 802 | 0 | 0 | 0 | 0 | 7 | 1 |
| 803 | 0 | 0 | 0 | 0 | 14 | 1 |
| 804 | 0 | 0 | 0 | 0 | 3 | 1 |
| 805 | 0 | 0 | 0 | 0 | 3 | 1 |
| 806 | 0 | 0 | 0 | 0 | 2 | 1 |
| 807 | 0 | 0 | 0 | 0 | 10 | 1 |
| 808 | 0 | 0 | 0 | 0 | 4 | 1 |
| 809 | 0 | 0 | 0 | 0 | 6 | 1 |
| 810 | 0 | 0 | 0 | 0 | 4 | 1 |
| 811 | 0 | 0 | 0 | 0 | 9 | 1 |
| 812 | 0 | 0 | 0 | 0 | 10 | 1 |
| 813 | 0 | 0 | 0 | 0 | 3 | 1 |
| 814 | 0 | 0 | 0 | 0 | 10 | 1 |
| 815 | 0 | 0 | 0 | 0 | 2 | 1 |
| 816 | 0 | 0 | 0 | 0 | 2 | 1 |
| 817 | 0 | 0 | 0 | 0 | 4 | 1 |
| 818 | 0 | 0 | 0 | 0 | 5 | 1 |
| 819 | 0 | 0 | 0 | 0 | 14 | 1 |
| 820 | 0 | 0 | 0 | 0 | 4 | 1 |
| 821 | 0 | 0 | 0 | 0 | 3 | 1 |
| 822 | 0 | 0 | 0 | 0 | 9 | 1 |
| 823 | 0 | 0 | 0 | 0 | 9 | 1 |
| 824 | 0 | 0 | 0 | 0 | 4 | 1 |
| 825 | 0 | 0 | 0 | 0 | 9 | 1 |
| 826 | 0 | 0 | 0 | 0 | 2 | 1 |
| 827 | 0 | 0 | 0 | 0 | 5 | 1 |
| 828 | 0 | 0 | 0 | 0 | 2 | 1 |
| 829 | 0 | 0 | 0 | 0 | 2 | 1 |
| 830 | 0 | 0 | 0 | 0 | 10 | 1 |
| 831 | 0 | 0 | 0 | 0 | 14 | 1 |
| 832 | 0 | 0 | 0 | 0 | 5 | 1 |
| 833 | 0 | 0 | 0 | 0 | 2 | 1 |
| 834 | 0 | 0 | 0 | 0 | 2 | 1 |
| 835 | 0 | 0 | 0 | 0 | 9 | 1 |
| 836 | 0 | 0 | 0 | 0 | 5 | 1 |
| 837 | 0 | 0 | 0 | 0 | 10 | 1 |
| 838 | 0 | 0 | 0 | 0 | 10 | 1 |
| 839 | 0 | 0 | 0 | 0 | 5 | 1 |
| 840 | 0 | 0 | 0 | 0 | 3 | 1 |
| 841 | 0 | 0 | 0 | 0 | 9 | 1 |
| 842 | 0 | 0 | 0 | 0 | 6 | 1 |
| 843 | 0 | 0 | 0 | 0 | 6 | 1 |
| 844 | 0 | 0 | 0 | 0 | 9 | 1 |
| 845 | 0 | 0 | 0 | 0 | 6 | 1 |
| 846 | 0 | 0 | 0 | 0 | 2 | 1 |
| 847 | 0 | 0 | 0 | 0 | 7 | 1 |
| 848 | 0 | 0 | 0 | 0 | 11 | 1 |
| 849 | 0 | 0 | 0 | 0 | 9 | 1 |
| 850 | 0 | 0 | 0 | 0 | 6 | 1 |
| 851 | 0 | 0 | 0 | 0 | 7 | 1 |
| 852 | 0 | 0 | 0 | 0 | 2 | 1 |
| 853 | 0 | 0 | 0 | 0 | 4 | 1 |
| 854 | 0 | 0 | 0 | 0 | 2 | 1 |
| 855 | 0 | 0 | 0 | 0 | 3 | 1 |
| 856 | 0 | 0 | 0 | 0 | 10 | 1 |
| 857 | 0 | 0 | 0 | 0 | 9 | 1 |
| 858 | 0 | 0 | 0 | 0 | 14 | 1 |
| 859 | 0 | 0 | 0 | 0 | 6 | 1 |
| 860 | 0 | 0 | 0 | 0 | 3 | 1 |
| 861 | 0 | 0 | 0 | 0 | 3 | 1 |
| 862 | 0 | 0 | 0 | 0 | 14 | 1 |
| 863 | 0 | 0 | 0 | 0 | 2 | 1 |
| 864 | 0 | 0 | 0 | 0 | 8 | 1 |
| 865 | 0 | 0 | 0 | 0 | 3 | 1 |
| 866 | 0 | 0 | 0 | 0 | 10 | 1 |
| 867 | 0 | 0 | 0 | 0 | 2 | 1 |
| 868 | 0 | 0 | 0 | 0 | 6 | 1 |
| 869 | 0 | 0 | 0 | 0 | 3 | 1 |
| 870 | 0 | 0 | 0 | 0 | 3 | 1 |
| 871 | 0 | 0 | 0 | 0 | 7 | 1 |
| 872 | 0 | 0 | 0 | 0 | 4 | 1 |
| 873 | 0 | 0 | 0 | 0 | 2 | 1 |
| 874 | 0 | 0 | 0 | 0 | 4 | 1 |
| 875 | 0 | 0 | 0 | 0 | 4 | 1 |
| 876 | 0 | 0 | 0 | 0 | 9 | 1 |
| 877 | 0 | 0 | 0 | 0 | 2 | 1 |
