# Supplementary material for: Inference of Transposable Element Ancestry
Source: PLoS Genet. 2014 Aug 14;10(8):e1004482. doi: 10.1371/journal.pgen.1004482 (PMC4133154; doi:10.1371/journal.pgen.1004482)
Supplement: Table S3 — MCMC results for AluSc candidate ancestors. (DOCX) [file pgen.1004482.s006.docx]

| Replicative frequency rank | Discriminatory site sequence | Estimated replicative frequency | Percentile 2.5 of replicative frequency | Percentile 97.5 of replicative frequency | Probability sequence replicated |
| --- | --- | --- | --- | --- | --- |
| 1 | ATAGAG | 663.354 | 628 | 697 | 1 |
| 2 | GTAGAG | 137.967 | 111 | 167 | 1 |
| 3 | ATAAAG | 73.202 | 53 | 94 | 1 |
| 4 | GT-GGG | 45.537 | 33 | 61 | 1 |
| 5 | AGAGAG | 19.367 | 0 | 33 | 0.915 |
| 6 | GTAAAA | 13.131 | 0 | 23 | 0.899 |
| 7 | GTAAAG | 8.403 | 0 | 37 | 0.35 |
| 8 | GT-AAG | 6.843 | 0 | 29 | 0.346 |
| 9 | GT-GAG | 3.359 | 0 | 33 | 0.145 |
| 10 | GGAGAG | 0.778 | 0 | 14 | 0.06 |
| 11 | GT-GGA | 0.059 | 0 | 0 | 0.007 |
| 12 | CT-GGG | 0 | 0 | 0 | 0 |
| 13 | ATCGAG | 0 | 0 | 0 | 0 |
| 14 | ATGCAG | 0 | 0 | 0 | 0 |
| 15 | A-AGAG | 0 | 0 | 0 | 0 |
| 16 | AAAAAG | 0 | 0 | 0 | 0 |
| 17 | CTAAAG | 0 | 0 | 0 | 0 |
| 18 | GTGGGA | 0 | 0 | 0 | 0 |
| 19 | GTAGGA | 0 | 0 | 0 | 0 |
| 20 | GGAAAG | 0 | 0 | 0 | 0 |
| 21 | GGAGAC | 0 | 0 | 0 | 0 |
| 22 | GCAGAG | 0 | 0 | 0 | 0 |
| 23 | ATTAAG | 0 | 0 | 0 | 0 |
| 24 | AT-GGG | 0 | 0 | 0 | 0 |
| 25 | AAAGAA | 0 | 0 | 0 | 0 |
| 26 | ATTGAC | 0 | 0 | 0 | 0 |
| 27 | ACGGAG | 0 | 0 | 0 | 0 |
| 28 | ACAG-G | 0 | 0 | 0 | 0 |
| 29 | GTTAGG | 0 | 0 | 0 | 0 |
| 30 | GTAG-G | 0 | 0 | 0 | 0 |
| 31 | ATCAAG | 0 | 0 | 0 | 0 |
| 32 | GTAGGT | 0 | 0 | 0 | 0 |
| 33 | GT-GAA | 0 | 0 | 0 | 0 |
| 34 | CTAGAG | 0 | 0 | 0 | 0 |
| 35 | ACAAAG | 0 | 0 | 0 | 0 |
| 36 | GTTAGC | 0 | 0 | 0 | 0 |
| 37 | ATAACG | 0 | 0 | 0 | 0 |
| 38 | CGAGAG | 0 | 0 | 0 | 0 |
| 39 | GTAGAC | 0 | 0 | 0 | 0 |
| 40 | AT-AAG | 0 | 0 | 0 | 0 |
| 41 | ATAAAA | 0 | 0 | 0 | 0 |
| 42 | GTTAAG | 0 | 0 | 0 | 0 |
| 43 | ATAGA- | 0 | 0 | 0 | 0 |
| 44 | ACACAG | 0 | 0 | 0 | 0 |
| 45 | GTGGAG | 0 | 0 | 0 | 0 |
| 46 | ATGGAT | 0 | 0 | 0 | 0 |
| 47 | ATGTAG | 0 | 0 | 0 | 0 |
| 48 | AGATAA | 0 | 0 | 0 | 0 |
| 49 | AGAGGG | 0 | 0 | 0 | 0 |
| 50 | ATAAAT | 0 | 0 | 0 | 0 |
| 51 | GTAGAT | 0 | 0 | 0 | 0 |
| 52 | GC-GAG | 0 | 0 | 0 | 0 |
| 53 | ATAGGG | 0 | 0 | 0 | 0 |
| 54 | GT-AGA | 0 | 0 | 0 | 0 |
| 55 | GTTGGA | 0 | 0 | 0 | 0 |
| 56 | AGAGAA | 0 | 0 | 0 | 0 |
| 57 | GTTGGG | 0 | 0 | 0 | 0 |
| 58 | GCAGAT | 0 | 0 | 0 | 0 |
| 59 | GTCGAG | 0 | 0 | 0 | 0 |
| 60 | GA-GGG | 0 | 0 | 0 | 0 |
| 61 | #NAME? | 0 | 0 | 0 | 0 |
| 62 | GT-AGG | 0 | 0 | 0 | 0 |
| 63 | TTAGAG | 0 | 0 | 0 | 0 |
| 64 | CGAAAG | 0 | 0 | 0 | 0 |
| 65 | ATAGAC | 0 | 0 | 0 | 0 |
| 66 | GTTGAG | 0 | 0 | 0 | 0 |
| 67 | AT-G-T | 0 | 0 | 0 | 0 |
| 68 | TT-GGG | 0 | 0 | 0 | 0 |
| 69 | ATAG-G | 0 | 0 | 0 | 0 |
| 70 | ATAAAC | 0 | 0 | 0 | 0 |
| 71 | GTAGAA | 0 | 0 | 0 | 0 |
| 72 | ATAAGG | 0 | 0 | 0 | 0 |
| 73 | ATATAG | 0 | 0 | 0 | 0 |
| 74 | AT-GAG | 0 | 0 | 0 | 0 |
| 75 | AAAGAG | 0 | 0 | 0 | 0 |
| 76 | GTAGGG | 0 | 0 | 0 | 0 |
| 77 | GTATGG | 0 | 0 | 0 | 0 |
| 78 | ATAGGA | 0 | 0 | 0 | 0 |
| 79 | ATAGTG | 0 | 0 | 0 | 0 |
| 80 | ATGGAG | 0 | 0 | 0 | 0 |
| 81 | ACAGAG | 0 | 0 | 0 | 0 |
| 82 | ATAGAA | 0 | 0 | 0 | 0 |
| 83 | ATAGAT | 0 | 0 | 0 | 0 |
| 84 | AGAAAG | 0 | 0 | 0 | 0 |
| 85 | ATTGAG | 0 | 0 | 0 | 0 |
| 86 | GTATAG | 0 | 0 | 0 | 0 |
| 87 | ATACAG | 0 | 0 | 0 | 0 |
| 88 | ATGGGG | 0 | 0 | 0 | 0 |
